# Minimizing Movements for the Generalized Power Mean Curvature Flow

**DOI:** 10.1007/s00032-024-00410-y

**Published:** 2024-12-10

**Authors:** Giovanni Bellettini, Shokhrukh Yu. Kholmatov

**Affiliations:** 1 University of Siena, Via Roma 56, 53100 Siena, Italy; 2 International Centre for Theoretical Physics (ICTP), Strada Costiera 11, 34151 Trieste, Italy; 3 University of Vienna, Oskar-Morgenstern Platz 1, 1090 Vienna, Austria

**Keywords:** Affine curvature flow, Power mean curvature flow, Almgren–Taylor–Wang functional, Minimizing movements, 35K93, 53E10, 35D30, 49Q20

## Abstract

Motivated by a conjecture of De Giorgi, we consider the Almgren–Taylor–Wang scheme for mean curvature flow, where the volume penalization is replaced by a term of the form

$$\begin{aligned} \int _{E\Delta F}f\left( \tfrac{\textrm{d}_{F}}{\tau }\right) ~dx \end{aligned}$$

for $f$ ranging in a large class of strictly increasing continuous functions, where $E \Delta F= (E \cup F){\setminus } E \cap F$ is the symmetric difference between sets *E* and *F*, and $d_F$ is the distance function from $\partial F$. In particular, our analysis covers the case

$$\begin{aligned} f( r ) = r^\alpha , \qquad r \ge 0, \ \ \alpha >0, \end{aligned}$$

considered by De Giorgi. We show that the generalized minimizing movement scheme converges to the geometric evolution equation

$$\begin{aligned} f(v) = - \kappa \quad \text {on }\partial E(t), \end{aligned}$$

where $\{E(t)\}$ are evolving subsets of ${\mathbb {R}}^n,$ $v$ is the normal velocity of $\partial E(t),$ and $\kappa$ is the mean curvature of $\partial E(t)$. We extend our analysis to the anisotropic setting, and in the presence of a driving force. We also show that minimizing movements coincide with the smooth classical solution as long as the latter exists. Finally, we prove that in the absence of forcing, mean convexity and convexity are preserved by the weak flow.

## Introduction

Denote by $\vert \cdot \vert$ and by $\mathcal {H}^{n-1}$ the Lebesgue measure and the $(n-1)$-dimensional Hausdorff measure in ${\mathbb {R}}^n$, respectively, let $B_\rho (x)$ be the open ball centered at $x\in {\mathbb {R}}^n$ of radius $\rho >0$, and $\Delta$ be the symmetric difference between sets.

In [17] De Giorgi poses the following two conjectures,^1^ related to some results in [2, 20, 23] and, more generally, to mean curvature flow.

### Conjecture 1.1

Let $\mathrm {Conv_b}({\mathbb {R}}^n)$ be the class of all open convex bounded subsets of ${\mathbb {R}}^n,$ endowed with the metric $d({{\mathcal {K}}}_1, {{\mathcal {K}}}_2) = \vert {{\mathcal {K}}}_1 \Delta {{\mathcal {K}}}_2 \vert .$ Given $\alpha \in (0, +\infty )$ and ${{\mathcal {K}}}_0\in \mathrm {Conv_b}({\mathbb {R}}^n)$, set

$$\begin{aligned} {\mathcal {G}}({{\mathcal {K}}}_2;{{\mathcal {K}}}_1,\tau ,k) = {\left\{ \begin{array}{ll} \vert {{\mathcal {K}}}_1\Delta {{\mathcal {K}}}_0\vert &  \hbox { if}\ k\le 0,\\ \displaystyle \mathcal {H}^{n-1}(\partial {{\mathcal {K}}}_2) + \frac{1}{\tau ^ \alpha }\int _{{{\mathcal {K}}}_2\Delta {{\mathcal {K}}}_1}\textrm{d}_{{{\mathcal {K}}}_1}(x)^\alpha ~dx &  \hbox { if}\ k>0, \end{array}\right. } \end{aligned}$$

where $\textrm{d}_F(\cdot ):=\textrm{dist}(\cdot ,\partial F)$ is the distance function from the boundary $\partial F$ of *F*. Then there exists a unique minimizing movement $E(\cdot )$ in $\mathrm {Conv_b}({\mathbb {R}}^n),$ associated to ${\mathcal {G}}$ and starting from ${{\mathcal {K}}}_0$ and, in the case $\alpha = 1$, $\partial E(t)$ moves along its mean curvature.

### Conjecture 1.2

Let

1.1 $$\begin{aligned} \mathcal {S}:=\Big \{E\subset {\mathbb {R}}^n:\,\,\vert E\vert <+\infty ,\,\,x\in E\,\,\Leftrightarrow \,\,\lim \limits _{\rho \rightarrow 0^+}\rho ^{-n}\vert B_\rho (x)\setminus E\vert =0\Big \}, \end{aligned}$$

endowed with the metric $d(E_1, E_2 ) = \vert E_1\Delta E_2\vert .$ Given $\alpha \in (0, +\infty ),$ $E_0\in \mathcal {S}$ such that $\mathcal {H}^{n-1}(\partial E_0)<+\infty$ and $g\in L^1({\mathbb {R}}^n)\cap L^n({\mathbb {R}}^n),$ set

$$\begin{aligned} {\widetilde{{\mathcal {G}}}}(E_2;E_1,\tau ,k) = {\left\{ \begin{array}{ll} \vert E_1\Delta E_0\vert &  \hbox { if}\ k\le 0,\\ \displaystyle \mathcal {H}^{n-1}(\partial E_2) + \int _{E_2}g(x)~dx + \frac{1}{\tau ^\alpha } \int _{E_2\Delta E_1}\textrm{d}_{E_1}(x)^\alpha ~dx &  \hbox { if}\ k>0. \end{array}\right. } \end{aligned}$$

Then there exists a generalized minimizing movement in $\mathcal {S},$ associated to ${\widetilde{{\mathcal {G}}}}$ and starting from $E_0.$

Clearly, if $g\equiv 0$, then ${\mathcal {G}}({{\mathcal {K}}}_2; {{\mathcal {K}}}_1, \tau , k) = \widetilde{\mathcal {G}}({{\mathcal {K}}}_2; {{\mathcal {K}}}_1, \tau , k)$ for ${{\mathcal {K}}}_1, {{\mathcal {K}}}_2 \in \mathrm {Conv_b}({\mathbb {R}}^n)$.

In this paper we prove Conjecture 1.2 in a more general form (see Theorem 1.4). We also establish weaker versions of Conjecture 1.1. More specifically: (a) minimizing ${\widetilde{{\mathcal {G}}}}$ in $\mathcal {S}$ (rather than in $\mathrm {Conv_b}({\mathbb {R}}^n)$) we obtain a unique minimizing movement (Theorem 1.6); (b) minimizing $\widetilde{\mathcal {G}}$ in $\mathrm {Conv_b}({\mathbb {R}}^n)$ we obtain the existence of generalized minimizing movements (Theorem 1.7). Of course, these two results are related; in fact, for $\alpha \in (0,1],$ using the convexity of the map $x\mapsto \textrm{dist}(x,\partial {{\mathcal {K}}})$ for convex sets ${{\mathcal {K}}}$ and the methods of [11], we can show that there exists a unique minimizing movement in $\mathrm {Conv_b}({\mathbb {R}}^n)$ which is also the unique minimizing movement in $\mathcal {S}$ (Corollary 8.2). At the moment we miss the proof of the uniqueness of generalized minimizing movements for $\alpha >1.$

The gradient flow of ${\widetilde{{\mathcal {G}}}}$, in case $g\equiv 0,$ leads to the equation

1.2 $$\begin{aligned} v= -\kappa ^{1/\alpha }, \end{aligned}$$

where $v$ and $\kappa$ are the normal velocity and the mean curvature of an evolving family $t \rightarrow \partial E(t)$ of smooth closed hypersurfaces in ${\mathbb {R}}^n$; (1.2) is called $\kappa ^{1/\alpha }$ (or power of mean curvature) flow [30, 31], and is meaningful also in the case of nonconvex sets provided $\alpha \in {\mathbb {N}}$ is odd [6]. When $\alpha =1,$ the evolution equation (1.2) is the classical mean curvature flow. When $\alpha =3$ and $\partial E(t)$ are evolving curves in $\mathbb {R}^2$, (1.2) is called the *affine curvature flow* (see e.g. [1, 3, 5, 6, 14, 15, 21, 29] and references therein) because of the invariance of the flow with respect to affine transformations of coordinates; this equation has applications in image processing [3, 10]. Depending on $\alpha$, various phenomena may occur. If $\alpha <8,$ the only embedded homothetically shrinking solutions are circles, except when $\alpha =3,$ where some self-shrinking ellipses occur, while if $\alpha \ge 8,$ a new family of symmetric curves resembling either circles or polygons arise (see e.g. [1, 5, 15]). The flow (1.2) exists, for instance, in the case of bounded convex initial subsets of ${\mathbb {R}}^n$ [30, 31], with finite-time extinction towards a point. See also [19], for an asymptotic study of a time-fractional Allen-Cahn equation and its convergence to the power mean curvature flow.

A by-product of our analysis is the study of a generalization of (1.2) of the form

1.3 $$\begin{aligned} f(v) = - (\kappa _\varphi + g), \end{aligned}$$

where $\varphi$ is an even anisotropy in ${\mathbb {R}}^n,$ $\kappa _\varphi$ is the anisotropic mean curvature of $\partial E(t),$ and $f: \mathbb {R}\rightarrow \mathbb {R}$ is a strictly increasing continuous odd surjective function. Clearly, in general we cannot expect invariance of solutions to (1.3) with respect to affine transformations; we refer to (1.3) as the *generalized power mean curvature flow*.

In this paper we study minimizing movement solutions corresponding to (1.3) under quite general assumptions on $f$ and $g$. Following [2, 12, 17] we introduce an Almgren–Taylor–Wang type functional – generalizing the functional^2^ in Conjecture 1.2:

$$\begin{aligned} \mathcal {F}_{\varphi ,f,g}(E;F,\tau ):=P_\varphi (E)+ \int _{E\Delta F}f\left( \tfrac{\textrm{d}_{F}}{\tau }\right) ~dx + \int _{E}g(x)~ dx \end{aligned}$$

with domain

$$\begin{aligned} \mathcal {S}^*:= BV({\mathbb {R}}^n;\{0,1\})\cap \mathcal {S}, \end{aligned}$$

where $$\tau >0$$ and

1.4 $$\begin{aligned} P_\varphi (E):=\int _{\partial ^*E}\varphi ^o(\nu _E)~d\mathcal {H}^{n-1} \end{aligned}$$

is the $\varphi$-perimeter of *E*. Here $\partial ^*E$ and $\nu _E$ denote the reduced boundary and the generalized outer unit normal of *E*, respectively, and $\varphi ^o$ is the dual of $\varphi$. When $\varphi (\cdot ) = \vert \cdot \vert$ is the Euclidean norm, we write *P* in place of $P_\varphi$; localization of the perimeter in a Borel set $A$ is denoted by $P(\cdot , A)$. When $\varphi$ is Euclidean and $f(r)=r$ we recover the standard Almgren–Taylor–Wang functional with a driving force [12]. In what follows, if no confusion is possible,

1.5 $$\begin{aligned} \text {we~ shorthand}~ \mathcal {F}_{\varphi ,f,g}\text {~with~ the~ symbol~} {\mathcal {F}}. \end{aligned}$$

Note that when $\varphi$ is Euclidean and $g\equiv 0,$ we have, for $B_\varrho = \{\xi \in {\mathbb {R}}^n: \vert \xi \vert < \varrho \}$,

$$\begin{aligned} {\mathcal {F}}(B_r;B_{r_0},\tau ) = n\omega _n r^{n-1} + n\omega _n\int _0^r f\Big (\tfrac{s-r_0}{\tau }\Big )\,s^{n-1}ds =:n\omega _n \ell (r) \end{aligned}$$

for any $r,r_0>0.$ Since $\ell$ is differentiable, we compute

$$\begin{aligned} \ell '(r) = (n-1)r^{n-2} + r^{n-1} f\Big (\tfrac{r-r_0}{\tau }\Big ),\quad r > 0. \end{aligned}$$

Thus, $r>0$ is a critical point if and only if

1.6 $$\begin{aligned} \frac{n-1}{r} + f\Big (\tfrac{r-r_0}{\tau }\Big ) = 0\quad \Longleftrightarrow \quad \frac{r - r_0}{\tau } = -f^{-1}\Big (\tfrac{n-1}{r} \Big ). \end{aligned}$$

Using this, in Theorem 4.4 we show that balls shrink self-similarly and their radii satisfy

$$\begin{aligned} r'(t) = -f^{-1}\Big (\tfrac{n-1}{r(t)}\Big )\quad \textrm{if}~ r(t)>0, \end{aligned}$$

consistently with (1.3). See Sect. 4 for more.

The next definition is a particular case of a definition given in [17].

### Definition 1.3

(*Flat flows, GMM and MM*) A family $\{E(t)\}_{t\in [0,+\infty )}\subset \mathcal {S}^*$ is called a *generalized minimizing movement* (shortly, $\textrm{GMM}$) in $\mathcal {S}^*$, associated to ${\mathcal {F}}$ and starting from $E_0\in S^*,$ if there exist a sequence $\tau _j\rightarrow 0^+$ and a family $\{E(\tau _j,k)\}_{j,k\ge 0}$ of sets, so-called flat flows, defined as $E(\tau _j,0) = E_0$ and

$$\begin{aligned} E(\tau _j,k)\in \textrm{argmin}\, {\mathcal {F}}(\cdot ;E(\tau _j,k-1),\tau _j),\quad k,j\ge 1, \end{aligned}$$

such that, for any $t\ge 0$,

1.7 $$\begin{aligned} E(\tau _j,\left\lfloor t/\tau _j \right\rfloor ) \rightarrow E(t) \quad \text {in } L_\textrm{loc}^1(\mathbb {R}^n) \text { as } j\rightarrow +\infty \end{aligned}$$

where $\left\lfloor x \right\rfloor$ is the integer part of $x\in \mathbb {R}.$ When $E(\cdot )$ in (1.7) is independent of the sequence $(\tau _j),$ i.e., the limit holds as $\tau \rightarrow 0^+,$ then it is called a minimizing movement (shortly MM) in $\mathcal {S}^*,$ associated to ${\mathcal {F}}$ and starting from $E_0.$ The set of $\textrm{GMM}$ and $\textrm{MM}$ in $\mathcal {S}^*$ will be denoted, respectively, as $\textrm{GMM}({\mathcal {F}},\mathcal {S}^*,E_0)$ and $\textrm{MM}({\mathcal {F}},\mathcal {S}^*,E_0).$

Now, we list a number of assumptions on $f$ and $g$ needed in the sequel.

### Hypothesis

**(H)**

- $f:\mathbb {R}\rightarrow \mathbb {R}$ is a strictly increasing, continuous, surjective odd function;
- for any $\varsigma _0,\varsigma _1>0$ and any $\tau >0,$ the unique solution $(\rho _\tau ,r_\tau )$ of the system
  1.8 $$\begin{aligned} {\left\{ \begin{array}{ll} \rho f\Big (\tfrac{\rho }{\tau }\Big ) = \varsigma _0,\\ r f\Big (\tfrac{r+2\rho }{\tau }\Big ) = \varsigma _1,\\ \rho ,r>0, \end{array}\right. } \end{aligned}$$
  satisfies^3^
  1.9 $$\begin{aligned} \liminf \limits _{\tau \rightarrow 0^+}\frac{r_\tau }{\tau }\in (0,+\infty ]; \end{aligned}$$
- $$g\in L^1({\mathbb {R}}^n)\cap L^p({\mathbb {R}}^n)$$ for some $$p\in [n, +\infty ].$$

When $\alpha =1,$ we have $\rho _\tau = \sqrt{\varsigma _0}\, \tau ^{1/2}$ and $r_\tau = (\sqrt{\varsigma _0+4\varsigma _1}- \sqrt{\varsigma _0})\tau ^{1/2}.$ More generally, when $r>0$ and $f(r)=r^\alpha$ for some $\alpha >0,$ we can explicitly find $\rho _\tau$ in (1.8): $\rho _\tau =\varsigma _0^\frac{1}{1+\alpha }\tau ^{\frac{\alpha }{1+\alpha }}.$ Moreover, $r_\tau >\varsigma _1^{1/\alpha }\tau ^{\frac{\alpha }{1+\alpha }}$ and by the Hölder inequality and the explicit expression of $\rho _\tau$ we have

$$\begin{aligned} \varsigma _1^{1/\alpha }\tau = r_\tau ^{\frac{1+\alpha }{\alpha }}+2\rho r_\tau ^{1/\alpha }\le r_\tau ^{\frac{1+\alpha }{\alpha }} + \tfrac{2\alpha \epsilon }{1+\alpha }\tau + \tfrac{1}{(1+\alpha )\epsilon } r_\tau ^{\frac{1+\alpha }{\alpha }} \end{aligned}$$

and thus, choosing $\epsilon >0$ small enough we find $r_\tau \ge \varsigma _2\tau ^{\frac{\alpha }{1+\alpha }}$ for some $\varsigma _2>0.$ In particular $r_\tau$ satisfies (1.9). See also Example 2.1.

Our first result is the following

### Theorem 1.4

(Existence, time-continuity and bounds of GMM) Assume Hypothesis (H) and use the notation (1.5). Then:

(i) For any $E_0\in \mathcal {S}^*,$ $\textrm{GMM}({\mathcal {F}},\mathcal {S}^*,E_0)$ is nonempty. Moreover, there exists a continuous strictly increasing function $\omega :[0,+\infty )\rightarrow [0,+\infty )$ (depending only on $f,$ *n*,  the constants $c_{\varphi }$ and $C_{\varphi }$ in (2.1), $P_\varphi (E_0)$ and $\Vert g^-\Vert _{L^1({\mathbb {R}}^n)}$) with $\omega (0)=0$ such that for any $E(\cdot )\in \textrm{GMM}({\mathcal {F}},\mathcal {S}^*,E_0)$
  1.10 $$\begin{aligned} |E(t)\Delta E(s)| \le \omega (|t-s|),\quad s,t>0. \end{aligned}$$
  If, additionally, $|\partial E_0|=0,$ then (1.10) holds for all $s,t\ge 0.$ Furthermore, if
  $$\begin{aligned} \exists \lim _{r\rightarrow +\infty } \frac{f(r)}{r^\alpha } \in (0,+\infty ) \end{aligned}$$
  for some $\alpha >0,$ then $\omega$ can be chosen locally $\frac{\alpha }{1+\alpha }$-Hölder continuous.
(ii) If $E_0$ is bounded and
  1.11 $$\begin{aligned} g^-(x) \le f(c_{g^-}(1+|x|))\quad \text {for all } |x|>c_{g^-}\end{aligned}$$
  for some sufficiently large constant $c_{g^-}>0,$ then each $\textrm{GMM}$ is locally bounded, i.e., for any $T>0$ there exists $R_T>0$ such that for any $E(\cdot )\in \textrm{GMM}({\mathcal {F}},\mathcal {S}^*,E_0)$
  $$\begin{aligned} E(t)\subset B_{R_T}(0)\quad \hbox { for all}\ t\in [0,T]. \end{aligned}$$

Some comments are in order.

- The oddness of $f$ allows to reduce ${\mathcal {F}}$ to a prescribed mean curvature functional: when $f$ is odd, we can write
  1.12 $$\begin{aligned} \int _{E\Delta F} f\left( \tfrac{\textrm{d}_{F}}{\tau }\right) ~dx = \int _E f\Big (\tfrac{\textrm{sd}_F}{\tau }\Big )~dx - \int _Ff\Big (\tfrac{\textrm{sd}_F}{\tau }\Big )~dx \end{aligned}$$
  whenever $E\cap F$ has finite measure, and $\textrm{sd}_F$ is the signed distance from $\partial F$ *positive outside* *F*. Indeed, one can readily check that $\chi _G\textrm{sd}_G\in L^1({\mathbb {R}}^n)$ for any *G* with $|G|<+\infty .$ Thus,
  $$\begin{aligned} \int _E f\Big (\tfrac{\textrm{sd}_F }{\tau }\Big )~dx - \int _Ff\Big (\tfrac{\textrm{sd}_F }{\tau }\Big )~dx =&\int _{E\setminus F} f\Big (\tfrac{\textrm{sd}_F }{\tau }\Big )~dx - \int _{F\setminus E}f\Big (\tfrac{\textrm{sd}_F }{\tau }\Big )~dx \\ =&\int _{E\setminus F} f\Big (\tfrac{\textrm{d}_F }{\tau }\Big )~dx + \int _{F\setminus E}f\Big (\tfrac{\textrm{d}_F }{\tau }\Big )~dx. \end{aligned}$$
  Therefore, similarly to the classical Almgren–Taylor–Wang functional,
  1.13 $$\begin{aligned} {\mathcal {F}}(E;F,\tau )= P_\varphi (E)+\int _{E}f\left( \tfrac{\textrm{sd}_{F}}{\tau }\right) ~dx + \int _{E}g(x) ~dx + c _F, \end{aligned}$$
  where $c _F$ is a constant independent of *E*. In view of (1.13), the minimization problem for ${\mathcal {F}}(\cdot ; F,\tau )$ is equivalent to the minimization of the prescribed mean curvature functional $E\in \mathcal {S}^*\mapsto P_\varphi (E) + \int _E h_{f,F,g}~dx,$ with
  1.14 $$\begin{aligned} h_{f,F,g}:=f\Big (\tfrac{\textrm{sd}_F}{\tau }\Big ) + g. \end{aligned}$$
- (1a) and (1c) suffice for the well-definiteness and the $L_\textrm{loc}^1({\mathbb {R}}^n)$-lower semicontinuity of ${\mathcal {F}}$, as well as for the existence of minimizers, and in particular, of flat flows (Lemma 3.1).
- Assumption $g\in L^p({\mathbb {R}}^n)$ in (1c) is used to establish the uniform density estimate
  1.15 $$\begin{aligned} \frac{P(E,B_r(x))}{r^{n-1}} \ge \theta _1,\quad x\in \partial E,\quad r\in (0,r_\tau ], \end{aligned}$$
  for minimizers *E* of ${\mathcal {F}}(\cdot ;F,\tau ),$ where $\theta _1>0$ is independent of *E*, *F* and $\tau$ (see Sects. 3.1, 3.2 and the inequality (3.15)).
- Assumption (1.9) together with (1.15) and Lemma A.1 applied with $\ell =\tau$ and $p>0,$ imply that any flat flow $\{E(\tau ,k)\}$ satisfies
  $$\begin{aligned}  &   |E(\tau ,k)\Delta E(\tau ,k-1)| \\  &   \qquad \le Cp^\sigma \tau P_\varphi (E(\tau ,k-1)) + \frac{1}{f(p)}\int _{E(\tau ,k)\Delta E(\tau ,k-1)} f\left( \tfrac{\textrm{d}_{E(\tau ,k-1)}}{\tau }\right) ~dx \end{aligned}$$
  for all $k\ge 2$ and for some $\sigma \in \{1,n\},$ where *C* is a coefficient depending only on *n*,  $\varphi ,$ $\theta$ and the liminf of $r_\tau /\tau$ in (1.9). This estimate, with a suitable choice of *p* (see (3.23)) yields the almost uniform time-continuity of the flat flow $t\mapsto E(\tau ,\left\lfloor t/\tau \right\rfloor )$ (see (3.24)), which in turn implies the existence of $\textrm{GMM}$ and the validity of (1.10).
- If $f(r)=r^\alpha$ for $r>0$ and for some $\alpha >0,$ then $\omega$ in (1.10) can be chosen as $\omega (t)=t^{\frac{\alpha }{1+\alpha }},$ see also (3.25).
- The strict monotonicity and surjectivity of $f$ are needed for the unique solvability of system (1.8). At the moment, we do not know what happens if these assumptions are dropped.
- Without assumption (1.9) our method fails to apply for the existence of $\textrm{GMM}$ (see Remark 3.2). However, we do not know whether $\textrm{GMM}$ exists or not without this assumption.
- If $f(r) \sim r^\alpha$ as $r\rightarrow +\infty$ for some $\alpha >0,$ then $f$ satisfies (1b), whereas if $f$ has exponential growth, then (1.9) may fail (see Examples 2.1 and 2.2).

To prove the existence and uniform time continuity of $\textrm{GMM}$ in Theorem 1.4 we follow the standard Almgren–Taylor–Wang method in [2, 12, 25]. However, the presence of $f$ requires some extra care in techniques. Furthermore, unlike these papers, for the existence of $\textrm{GMM}$ we do not assume a priori the boundedness of the initial sets and also of $g$. Finally, the uniform boundedness of $\textrm{GMM}$ will be done employing the isoperimetric properties of (anisotropic) balls. This can be also done following [12, Lemma 3.9], where a time-dependent bounded $g$ is considered.

It is well-known [8, Theorem 12] that the classical mean curvature flow preserves mean convexity; our next main qualitative result is a similar preservation (Sect. 5), which to some extent generalizes [13].

### Theorem 1.5

(Mean convex evolution) Suppose that $f$ satisfies (1a), (1b) and $g\equiv 0$. Then any $E(\cdot ) \in \textrm{GMM}({\mathcal {F}},\mathcal {S}^*,E_0)$ starting from a bounded $\delta$-mean convex set $E_0\Subset \Omega$ (in some open set $\Omega$) is itself a flow of $\delta$-mean convex sets in $\Omega .$ Moreover, the maps $t\mapsto E(t)$ and $t\mapsto P_\varphi (E(t))$ are nonincreasing.

To prove this theorem we partially follow the ideas of [13]; in order to show the $\delta$-mean convexity of minimizers of ${\mathcal {F}},$ the authors of [13] used the so-called Chambolle scheme for mean curvature flow and the Anzelotti-pairings, while here we employ comparison properties for the prescribed mean curvature functional.

Classical mean curvature flow even preserves convexity [23]. Also, the $\textrm{GMM}$ starting from a convex set is unique (which positively answers to the last assertion of Conjecture 1.1 when $\alpha =1$), see [8]. The following result shows the validity of this property also in the generalized power mean curvature flow setting (see Sect. 7).

### Theorem 1.6

(Evolution of convex sets and stability) Let $\varphi$ be the Euclidean norm, $g\equiv 0$ and $f(r) = r^\alpha$ for $r>0$, with $\alpha >0$. Let ${{\mathcal {K}}}_0\in \mathrm {Conv_b}({\mathbb {R}}^n)$. Then:

(i) $\textrm{GMM}({\mathcal {F}},\mathcal {S}^*,{{\mathcal {K}}}_0)= \textrm{MM}({\mathcal {F}},\mathcal {S}^*,{{\mathcal {K}}}_0) = \{{{\mathcal {K}}}(\cdot )\}$ and ${{\mathcal {K}}}(t)$ is convex for any $t\ge 0.$ Moreover, if ${{\mathcal {K}}}_0$ is smooth, then ${{\mathcal {K}}}(\cdot )$ is the smooth convex power mean curvature evolution starting from ${{\mathcal {K}}}_0$ (see Theorem 7.1);
(ii) Let ${{\mathcal {K}}}_0\in \mathrm {Conv_b}({\mathbb {R}}^n)$ and let $({{\mathcal {K}}}_{0 h})$ be any sequence of sets such that $\partial {{\mathcal {K}}}_{0 h}\rightarrow \partial {{\mathcal {K}}}_0$ in the Kuratowski sense and $(P(\mathcal {K}_{0h}))$ is uniformly bounded. Let ${{\mathcal {K}}}_h(\cdot ) \in \textrm{GMM}({\mathcal {F}},\mathcal {S}^*,{{\mathcal {K}}}_{0 h}),$ and let $\{{{\mathcal {K}}}(\cdot )\}$ be the unique minimizing movement starting from ${{\mathcal {K}}}_0$. Then
  $$\begin{aligned} \lim \limits _{h\rightarrow +\infty }|{{\mathcal {K}}}_h(t)\Delta {{\mathcal {K}}}(t)| = 0\quad \hbox { for any}\ t\ge 0. \end{aligned}$$

Since any convex set is also mean convex, from Theorem 1.5 it follows that each ${{\mathcal {K}}}(t)$ is mean convex and $t\mapsto {{\mathcal {K}}}(t)$ is nonincreasing.

We expect similar uniqueness and stability properties for generalized curvature flow of convex sets also in the anisotropic case with more general $f$. However, we leave this problem for future investigations.

Theorem 1.6 is a weak formulation of Conjecture 1.1, where the minimization problem for ${\mathcal {F}}$ is conducted in the larger class $\mathcal {S}^*$ rather than in the class $\mathrm {Conv_b}({\mathbb {R}}^n)$. Indeed, in $\mathrm {Conv_b}({\mathbb {R}}^n)$ we cannot apply the cutting-filling with balls argument used in the proof of Theorem 1.4; rather, using minimal cutting properties of convex sets we can show that the flat flows in $\mathrm {Conv_b}({\mathbb {R}}^n)$ and their perimeter have the following monotonicity: $E(\tau ,0)\supset E(\tau ,1)\supset \ldots$ and $P(E(\tau ,0))\ge P(E(\tau ,1))\ge \ldots .$ Thus, the map $t\mapsto P(E(\tau ,\left\lfloor t/\tau \right\rfloor ))$ is bounded and nonincreasing, and therefore sequentially compact (w.r.t. $\tau$) in $L_\textrm{loc}^1(\mathbb {R}_0^+).$ Now using convexity we conclude that every limit point is indeed a $\textrm{GMM}$ (Sect. 8):

### Theorem 1.7

(GMM in the class $\mathrm {Conv_b}({\mathbb {R}}^n)$) Assume $\varphi$ is an anisotropy in ${\mathbb {R}}^n,$ $f$ satisfies Hypothesis (1a) and (1b) and $g\equiv 0.$ For any ${{\mathcal {K}}}_0\in \mathrm {Conv_b}({\mathbb {R}}^n)$, $\textrm{GMM}( {\mathcal {F}},\mathrm {Conv_b}({\mathbb {R}}^n),{{\mathcal {K}}}_0)$ is nonempty. Moreover, if $\varphi$ is the Euclidean norm and $f(r)=r^\alpha$ for $r>0$ and some $\alpha \in (0,1],$ then $\textrm{GMM}( {\mathcal {F}},\mathrm {Conv_b}({\mathbb {R}}^n),{{\mathcal {K}}}_0)$ is a singleton and concides with the unique minimizing movement in $\textrm{MM}( {\mathcal {F}},\mathcal {S}^*,{{\mathcal {K}}}_0).$

We observe that we miss the proof of uniqueness of the minimizing movement in $\mathrm {Conv_b}({\mathbb {R}}^n)$ for $\alpha >1$.

Our last result is consistency of $\textrm{GMM}$ with the smooth classical solution of (1.3).^4^ Assuming the latter exists and is stable (in the sense of Definition 6.1), following some of the ideas of [2, 24] we show:

### Theorem 1.8

(Consistency) Suppose that $f \in C^\beta (\mathbb {R})$ and $g \in C^{\beta }({\mathbb {R}}^n)$ for some $\beta \in (0,1]$. Assume that $\varphi$ is an elliptic $C^3$-anisotropy and (1.3) admits a unique smooth stable solution $\{S(t)\}_{t\in [0,T)}$. Then for every $E(\cdot ) \in \textrm{GMM}({\mathcal {F}},\mathcal {S}^*,S(0))$ we have

$$\begin{aligned} E(t) = S(t)\quad \hbox { for all}\ t\in [0,T). \end{aligned}$$

As it happens for the classical consistency proof for the Almgren–Taylor–Wang functional, the proof of this nontrivial theorem heavily relies on the stability of the flow, strong comparison principles and discrete comparison principles.

The paper is organized as follows. In Sect. 2, after setting the notation, we quickly show some examples of interesting functions $f$. In Sect. 3 we prove Theorem 1.4. Various comparison results are studied in Sect. 4. The evolution of mean convex sets and convex sets are considered in Sects. 5, 7, and 8. The consistency of GMM with smooth solutions is proven in Sect. 6. Finally, we conclude the paper with two appendices, where we establish some technical results, needed in various proofs.

Shortly after the conclusion of this paper, we became aware of the paper [16], where the author addresses a similar problem, showing existence of level set solutions to the power mean curvature flow, via the minimizing movements. That paper appears to be completely independent of the present paper.

## Notation and Examples of Functions $f$

We write $\mathbb {R}^+:=(0,+\infty ),$ $\mathbb {R}_0^+:=[0,+\infty )$ and $\mathbb {N}_0=\mathbb {N}\cup \{0\}.$ The inclusion $E\Subset F$ means that *E* is bounded, $E \subset F$ and $\textrm{dist}(\partial E,\partial F)>0;$ we also set $E^c:= {\mathbb {R}}^n{\setminus } E$. Unless otherwise stated, $B_r:=B_r(0).$ In view of the definition of $\mathcal {S}$ in (1.1), every set *E* we consider coincides with the set $E^{(1)}$ of its points of density 1. Therefore $\partial E = \overline{\partial ^* E}$ and $\textrm{d}_E(\cdot ) =\textrm{dist}(\cdot ,\partial E) = \textrm{dist}(\cdot ,\partial ^* E).$ We denote by $\overline{co}\left( E\right)$ the closed convex hull of *E*. An anisotropy $\varphi : {\mathbb {R}}^n\rightarrow [0,+\infty )$ is a positively one-homogeneous even convex function equivalent to the Euclidean norm; its dual is defined as

$$\begin{aligned} \varphi ^o(\eta ) = \sup _{\varphi (\xi )=1} \,\,\xi \cdot \eta =1, \end{aligned}$$

so that $\varphi ^o$ is an anisotropy and

2.1 $$\begin{aligned} c_\varphi |\xi | \le \varphi ^o(\xi ) \le C_\varphi |\xi |,\quad \xi \in {\mathbb {R}}^n, \end{aligned}$$

for some $$C_\varphi \ge c_\varphi >0$$.

Recall the following anisotropic isoperimetric inequality [26]:

2.2 $$\begin{aligned} P_\varphi (E) \ge c_{n, \varphi }|E|^{\frac{n-1}{n}},\quad c_{n, \varphi }:= \frac{P_\varphi ({B_\varphi })}{|{B_\varphi }|^\frac{n-1}{n}}, \end{aligned}$$

where $P_\varphi$ is defined in (1.4), $E\in \mathcal {S}$ and^5^${B_\varphi }:=\{\varphi \le 1\}$. In the Euclidean case $\varphi (\cdot ) = \vert \cdot \vert$, $c_{n,|\cdot |} = n\omega _n^{1/n}$, $B = B_{\vert \cdot \vert }$, and $\kappa _\varphi =\kappa$. Our conventions are: the mean curvature is positive for (uniformly) convex sets, and the normal velocity is increasing for expanding sets.

Let us give some examples of function $f$.

### Example 2.1

($f$* a power*) Assume that $f$ is strictly increasing, continuous and

2.3 $$\begin{aligned} c_1r^{\alpha _1} \le f(r)\le c_2r^{\alpha _2}\quad \text {for all large }r>1 \end{aligned}$$

for some $0<c_1\le c_2$ and $0<\alpha _1 \le \alpha _2 \le \alpha _1+1.$ Then for any $\tau >0$ the system (1.8) is solvable and $r_\tau$ satisfies (1.9). Indeed, solvability of (1.8) follows from the continuity and the monotonicity of *f*. In particular, by the inverse monotone function theorem both maps $\tau \mapsto \rho _\tau$ and $\tau \mapsto r_\tau$ are continuous and increasing. Moreover, clearly, $\rho _\tau ,r_\tau \rightarrow 0$ as $\tau \rightarrow 0^+.$ Now by (2.3) and the equality $\rho _\tau f(\rho _\tau /\tau ) = C_0$

2.4 $$\begin{aligned} c_1' \tau ^{\frac{\alpha _2}{1+\alpha _2}} \le \rho _\tau < c_2' \tau ^{\frac{\alpha _1}{1+\alpha _1}} \end{aligned}$$

for all small $\tau >0$ and for some $c_2'\ge c_1'>0$ independent of $\tau .$ Similarly, from the equality $r_\tau f(\frac{r_\tau +2\rho _\tau }{\tau }) = C_1$ and estimates (2.3) and (2.4) we get

$$\begin{aligned} c_1'' \tau \le r_\tau ^{1/\alpha _2} (r_\tau + 2c_2' \tau ^{\frac{\alpha _1}{1+\alpha _1}}) = r_\tau ^{1+1/\alpha _2} + c_2' r_\tau ^{1/\alpha _2} \tau ^{\frac{\alpha _1}{1+\alpha _1}} \end{aligned}$$

for all small $\tau >0$ and for some $c_1''>0.$ This inequality implies either $\frac{\tau }{r_\tau ^{1+1/\alpha _2}}$ or $\frac{\tau }{r_\tau ^{1/\alpha _2}\tau ^{\frac{\alpha _1}{1+\alpha _1}}}$ is uniformly bounded. Since $\alpha _1\le \alpha _2\le 1+\alpha _1,$ either condition implies $r_\tau \ge c\tau$ for some $c>0$ and all small $\tau >0.$ Therefore, (1.9) holds.

### Example 2.2

($f$* an exponential*) Let $f(r)= e^r$ for large $r>0.$ Then $\rho _\tau$ in (1.8) satisfies

2.5 $$\begin{aligned} \rho _\tau = -\tau \ln \rho _\tau + \tau \ln \varsigma _0. \end{aligned}$$

For sufficiently small $\tau >0$, setting $\rho _\tau = \tau \ln (\tau ^{-1}) (1+u)$ in (2.5) we get

$$\begin{aligned} F(u,x,y):= u +x + [\ln (1+u) - \ln \varsigma _0]y=0,\quad x: = \tfrac{\ln \ln (\tau ^{-1})}{\ln (\tau ^{-1})},\quad y:=\tfrac{1}{\ln (\tau ^{-1})}. \end{aligned}$$

This analytic implicit function admits a unique solution $u=w(x,y),$ where $w(\cdot ,\cdot )$ is some real analytic function in a neighborhood of (0, 0), and thus, for sufficiently small $\tau >0,$

2.6 $$\begin{aligned} \rho _\tau = \tau \ln (\tau ^{-1})\Big [1+ w\Big ( \tfrac{\ln \ln (\tau ^{-1})}{\ln (\tau ^{-1})}, \tfrac{1}{\ln (\tau ^{-1})} \Big )\Big ]. \end{aligned}$$

Moreover, since $r_\tau$ satisfies the equation

$$\begin{aligned} \frac{r_\tau + 2\rho _\tau }{\tau } = \ln \varsigma _1 + \ln (r_\tau ^{-1}), \end{aligned}$$

we have

2.7 $$\begin{aligned} \frac{r_\tau }{\tau \ln (r_\tau ^{-1}) } + \frac{\rho _\tau }{\tau \ln (\rho _\tau ^{-1})}\frac{2\ln (\rho _\tau ^{-1})}{\ln (r_\tau ^{-1})} = 1 +\frac{\ln \varsigma _1}{\ln (r_\tau ^{-1})}. \end{aligned}$$

In view of (2.5), we have

$$\begin{aligned} \lim \limits _{\tau \rightarrow 0^+} \frac{\rho _\tau }{\tau \ln (\rho _\tau ^{-1})} = 1. \end{aligned}$$

Therefore, using $\frac{r_\tau }{\tau \ln (r_\tau ^{-1}) }\ge 0$ and recalling that $r_\tau \rightarrow 0^+,$ from (2.7) we get

$$\begin{aligned} \limsup _{\tau \rightarrow 0} \frac{2\ln (\rho _\tau ^{-1})}{\ln (r_\tau ^{-1})} \le 1. \end{aligned}$$

In particular, for sufficiently small $\tau >0,$

$$\begin{aligned} \frac{2\ln (\rho _\tau ^{-1})}{\ln (r_\tau ^{-1})} \le \frac{3}{2}. \end{aligned}$$

This and (2.6) imply

$$\begin{aligned} \frac{r_\tau }{\tau } \le \frac{\rho _\tau ^{4/3}}{\tau } = \tau ^{1/3}[\ln (\tau ^{-1}) (1+w)]^{4/3} \rightarrow 0 \end{aligned}$$

as $\tau \rightarrow 0^+.$ Hence, $f$ does not satisfy (1.9).

Throughout the paper we always assume $n\ge 2.$ We refer to [28] for the case $n=1$.

## Proof of Theorem 1.4

This section is devoted to the proof of Theorem 1.4. Assume as usual the convention (1.5).

### Lemma 3.1

Suppose that $f$ satisfies (1a) and that $g\in L^1({\mathbb {R}}^n)$. Then for any $F\in \mathcal {S}^*$ and $\tau >0$ there exists a minimizer of ${\mathcal {F}}(\cdot ;F,\tau )$ in $\mathcal {S}^*$.

As in the case of the prescribed mean curvature functional, the assumption $g\in L^1({\mathbb {R}}^n)$ can be relaxed to $g^-\in L^1({\mathbb {R}}^n)$.

### Proof

Let us study the minimum problem

$$\begin{aligned} \inf _{E\in \mathcal {S}^*}\,\,\, {\mathcal {F}}(E;F,\tau ). \end{aligned}$$

Let $(E_i)\subset \mathcal {S}^*$ be a minimizing sequence. We may assume

$$\begin{aligned} {\mathcal {F}}(E_i;F,\tau )\le {\mathcal {F}}(F;F,\tau )\quad \hbox { for all}\ i\ge 1. \end{aligned}$$

Then

$$\begin{aligned} P_\varphi (E_i) + \int _{E_i\Delta F} f\left( \tfrac{\textrm{d}_{F}}{\tau }\right) ~dx \le {\mathcal {F}}(F;F,\tau ) + \int _{{\mathbb {R}}^n} |g| ~dx:=C. \end{aligned}$$

In particular, by the nonnegativity of $f$ in $\mathbb {R}_0^+,$ the sequence $(P_\varphi (E_i))$ is bounded, and hence, by compactness, there exists $E\in BV_\textrm{loc}({\mathbb {R}}^n;\{0,1\})$ such that, up to a not relabelled subsequence, $E_i \rightarrow E$ in $L^1_\textrm{loc}({\mathbb {R}}^n)$ as $i\rightarrow +\infty .$ By the $L_\textrm{loc}^1$-lower semicontinuity of the anisotropic perimeter,

$$\begin{aligned} P_\varphi (E) \le \liminf _{i\rightarrow +\infty } P_\varphi (E_i)\le C<+\infty . \end{aligned}$$

Moreover, by the $L_\textrm{loc}^1$-convergence and the isoperimetric inequality (2.2), for any bounded open set $U\subset {\mathbb {R}}^n$

$$\begin{aligned} |U\cap E| = \lim \limits _{i\rightarrow +\infty } |U\cap E_i| \le \liminf \limits _{i\rightarrow +\infty } |E_i| \le (c_{n, \varphi }^{\frac{n}{n-1}})^{-1} \,\liminf \limits _{i\rightarrow +\infty } P_\varphi (E_i)^{\frac{n}{n-1}} \le \tfrac{C^{\frac{n}{n-1}}}{c_{n, \varphi }^{\frac{n}{n-1}}}. \end{aligned}$$

Thus, letting $U\nearrow {\mathbb {R}}^n$ we get $|E|<+\infty$ and hence, $E\in \mathcal {S}^*.$ By the $L_\textrm{loc}^1$-lower semicontinuity of ${\mathcal {F}}(\cdot ;F,\tau ),$ *E* is a minimizer. $\square$

### $L^\infty$-bound for $\textrm{d}_F$

Assume Hypothesis (H). For $F\in \mathcal {S}^*$ and $\tau >0,$ let $F_\tau$ be a minimizer of ${\mathcal {F}}(\cdot ;F,\tau ).$ In (3.10), on the basis of (3.6), we establish an upper bound for

$$\begin{aligned} \sup _{F_\tau \Delta F}\,\textrm{d}_F, \end{aligned}$$

which is necessary to get the density estimates in Sect. 3.2 centered at points of $\partial F_\tau$.

Take any $x\in \overline{F_\tau }\setminus F$ and set

$$\begin{aligned} r_x:=\textrm{d}_F(x)>0, \qquad B_r:=B_r(x). \end{aligned}$$

We observe that $B_r\cap F=\emptyset$ for all $r\in (0,r_x),$ so that $F\setminus F_\tau = F\setminus (F_\tau \setminus B_r).$ Then, by the minimality of $F_\tau ,$

3.1 $$\begin{aligned}  &   0\le {\mathcal {F}}(F_\tau \setminus B_r; F,\tau ) - {\mathcal {F}}(F_\tau ; F, \tau ) \nonumber \\  &   \quad = P_\varphi (F_\tau \setminus B_r) - P_\varphi (F_\tau ) -\int _{F_\tau \cap B_r} f\left( \tfrac{\textrm{d}_{F}}{\tau }\right) dy - \int _{F_\tau \cap B_r} gdy. \end{aligned}$$

Using the properties of the reduced boundary [26, Theorem 16.3], (2.1) and the Euclidean isoperimetric inequality, for a.e. $r\in (0,r_x)$ we get

3.2 $$\begin{aligned} \begin{aligned} P_\varphi (F_\tau \setminus B_r) - P_\varphi (F_\tau ) =&\,\int _{F_\tau \cap \partial B_r} \varphi ^o(-\nu _{B_r})d\mathcal {H}^{n-1} - P_\varphi (F_\tau ,B_r) \\ =&\, \int _{F_\tau \cap \partial B_r} \Big (\varphi ^o(-\nu _{B_r}) + \varphi ^o(\nu _{B_r}) \Big ) d\mathcal {H}^{n-1} - P_\varphi (F_\tau \cap B_r) \\ \le&\, 2C_{\varphi }\mathcal {H}^{n-1}(F_\tau \cap \partial B_r) - c_{\varphi }n\omega _n^{1/n}|F_\tau \cap B_r|^{\frac{n-1}{n}}. \end{aligned}\nonumber \\ \end{aligned}$$

Since $\textrm{d}_F \ge r_x-r$ in $B_r,$ by the increasing monotonicity of $f$ we obtain

$$\begin{aligned} -\int _{F_\tau \cap B_r} f\left( \tfrac{\textrm{d}_{F}}{\tau }\right) dy \le -f\Big (\frac{r_x-r}{\tau }\Big )|F_\tau \cap B_r|. \end{aligned}$$

Finally, if we also assume $r<\gamma _g,$ with $\gamma _g>0$ as in (A.3), then

3.3 $$\begin{aligned} \int _{F_\tau \cap B_r} gdy \le \frac{c_{\varphi }n\omega _n^{1/n}}{4}|F_\tau \cap B_r|^{\frac{n-1}{n}}. \end{aligned}$$

Inserting (3.2)–(3.3) in (3.1) we get

3.4 $$\begin{aligned} 2C_{\varphi }\mathcal {H}^{n-1}(F_\tau \cap \partial B_r) \ge f\Big (\frac{r_x-r}{\tau }\Big )|F_\tau \cap B_r| + \frac{3c_{\varphi }n\omega _n^{1/n}}{4}|F_\tau \cap B_r|^{\frac{n-1}{n}} \end{aligned}$$

for a.e. $r\in (0, r_x\wedge \gamma _g).$ Since $r_x>r$ and $f>0$ in $\mathbb {R}^+,$ this inequality implies

$$\begin{aligned} \mathcal {H}^{n-1}(F_\tau \cap \partial B_r) \ge \frac{3c_{\varphi }n\omega _n^{1/n}}{8C_{\varphi }}|F_\tau \cap B_r|^{\frac{n-1}{n}}, \end{aligned}$$

or, after integration,

3.5 $$\begin{aligned} |F_\tau \cap B_r| \ge \Big (\tfrac{3c_{\varphi }}{8C_{\varphi }}\Big )^n \omega _nr^n,\quad r\in (0, r_x\wedge \gamma _g]. \end{aligned}$$

Plugging this volume density estimate in (3.4) and using the positivity of the last term, we get

$$\begin{aligned} f\Big (\frac{r_x-r}{\tau }\Big )\,\Big (\tfrac{3c_{\varphi }}{8C_{\varphi }}\Big )^n \omega _nr^n \le 2C_{\varphi }\mathcal {H}^{n-1}(F_\tau \cap \partial B_r) \le 2 C_{\varphi }n\omega _n r^{n-1}, \end{aligned}$$

and hence, by the continuity of $f,$

3.6 $$\begin{aligned} rf\Big (\frac{r_x-r}{\tau }\Big ) \le C_1:= 2C_{\varphi }n \Big (\tfrac{8 C_{\varphi }}{3c_{\varphi }}\Big )^n,\quad r\in [0,r_x\wedge \gamma _g]. \end{aligned}$$

Now, assume $x\in \overline{F}\setminus F_\tau$ and set again $r_x:=\textrm{d}_F(x)>0.$ In this case for a.e. $r\in (0,r_x\wedge \gamma _g),$ we use the properties of the reduced boundary, (2.1) and the Euclidean isoperimetric inequality to get

$$\begin{aligned} P_\varphi (F_\tau \cup B_r) - P_\varphi (F_\tau ) =&\, 2\int _{F_\tau ^c\cap \partial B_r} \Big (\varphi ^o(\nu _{B_r}) + \varphi ^o(-\nu _{B_r}) \Big ) d\mathcal {H}^{n-1} - P_{\varphi }(F_\tau \cap B_r) \\ \le&\, 2C_{\varphi }\mathcal {H}^{n-1}(F_\tau ^c\cap \partial B_r) - c_{\varphi }n\omega _n^{1/n}|F_\tau ^c\cap B_r|^{\frac{n-1}{n}}. \end{aligned}$$

Moreover, since $\textrm{d}_F \ge r_x-r$ in $B_r$ (recall that $B_r\subset F$ by the choice of $r_x$), by the monotonicity of $f$

$$\begin{aligned} -\int _{F_\tau ^c\cap B_r} f\left( \tfrac{\textrm{d}_{F}}{\tau }\right) dy \le -f\Big (\frac{r_x-r}{\tau }\Big )|F_\tau ^c\cap B_r|. \end{aligned}$$

Finally, using $r<\gamma _g$ and the Morrey-type extimate (A.3) we find

$$\begin{aligned} \int _{F_\tau ^c\cap B_r} gdy \le \frac{c_{\varphi }n\omega _n^{1/n}}{4}|F_\tau ^c\cap B_r|^{\frac{n-1}{n}}. \end{aligned}$$

Inserting these estimates in

$$\begin{aligned}  &   0\le {\mathcal {F}}(F_\tau \cup B_r; F,\tau )- {\mathcal {F}}(F_\tau ;f,\tau ) \\  &   \quad = P_\varphi (F_\tau \cup B_r) - P_\varphi (F) - \int _{F_\tau ^c\cap B_r}f\left( \tfrac{\textrm{d}_{F}}{\tau }\right) ~dy + \int _{F_\tau ^c\cap B_r} g~dy, \end{aligned}$$

we get

$$\begin{aligned} 2C_{\varphi }\mathcal {H}^{n-1}(F_\tau ^c\cap \partial B_r) \ge f\Big (\frac{r_x-r}{\tau }\Big )|F_\tau ^c\cap B_r| + \frac{3c_{\varphi }n\omega _n^{1/n}}{4}|F_\tau ^c\cap B_r|^{\frac{n-1}{n}}, \end{aligned}$$

and hence, repeating the same aguments above, we find the same inequality as (3.6). Thus, for any $x\in \overline{F_\tau \Delta F}$ inverting (3.6), we have

3.7 $$\begin{aligned} r_x \le r + \tau f^{-1}\Big (\tfrac{C_1}{r}\Big ),\qquad r_x:=\textrm{d}_F(x)>0, \quad r\in (0, r_x\wedge \gamma _g). \end{aligned}$$

For any $\tau >0$ let $\rho _\tau :=\rho _\tau (C_1)>0$ be the unique number (compare (1b)) such that

3.8 $$\begin{aligned} \rho _\tau f\Big (\frac{\rho _\tau }{\tau }\Big ) = C_1. \end{aligned}$$

Clearly, $\tau \mapsto \rho _\tau$ is continuous in $\mathbb {R}^+$ and $\rho _\tau \rightarrow 0^+$ as $\tau \rightarrow 0^+.$ In particular

3.9 $$\begin{aligned} \exists \tau _0 = \tau _0(f,g) > 0: \rho _\tau \le \gamma _g\quad \hbox { for all}\ \tau \in (0,\tau _0). \end{aligned}$$

Let us estimate $r_x$ with a multiple of $\rho _\tau .$ For $\tau \in (0,\tau _0),$ if $r_x\le \rho _\tau ,$ we are done. If $\rho _\tau <r_x,$ by the choice of $\tau _0,$ we can apply (3.7) with $r=\rho _\tau$ and the equality (3.8) to get

$$\begin{aligned} r_x \le \rho _\tau + \tau f^{-1}\Big (\tfrac{C_1}{\rho _\tau }\Big ) = 2\rho _\tau . \end{aligned}$$

Thus we finally get the desired $L^\infty$-bound

3.10 $$\begin{aligned} \sup _{x\in \overline{F_\tau \Delta F}}\,\textrm{d}_F(x) \le 2\rho _\tau ,\quad \tau \in (0,\tau _0). \end{aligned}$$

This estimate will be used also in Theorem 4.3.

### Density Estimates

Assume Hypothesis (H) and for $F\in \mathcal {S}^*,$ $\tau _0>0$ (defined in (3.9)) and $\tau \in (0,\tau _0)$ let $F_\tau$ be a minimizer of ${\mathcal {F}}(\cdot ;F,\tau ).$ Let $\gamma _g>0$ be as in (A.3). We establish uniform density estimates for minimizers $F_\tau$ in balls $B_r(x),$ centered at

$$\begin{aligned} x\in \partial F_\tau \end{aligned}$$

(see (3.13), (3.14) and (3.15)). Basically, we use the arguments of Sect. 3.1 and the function $\tau \rightarrow \rho _\tau$ in (3.8), but some extra care is needed because now

$$\begin{aligned} B_r=B_r(x) \end{aligned}$$

may intersect the boundary of *F* and we need to estimate the differences

$$\begin{aligned}  &   \int _{(F_\tau \setminus B_r)\Delta F} f\left( \tfrac{\textrm{d}_{F}}{\tau }\right) dy - \int _{F_\tau \Delta F}f\left( \tfrac{\textrm{d}_{F}}{\tau }\right) dy \\  &   \quad = -\int _{B_r\cap (F_\tau \setminus F)} f\left( \tfrac{\textrm{d}_{F}}{\tau }\right) dy + \int _{B_r\cap F_\tau \cap F} f\left( \tfrac{\textrm{d}_{F}}{\tau }\right) dy \le \int _{B_r\cap F_\tau \cap F} f\left( \tfrac{\textrm{d}_{F}}{\tau }\right) dy \end{aligned}$$

and

$$\begin{aligned}  &   \int _{(F_\tau \cup B_r)\Delta F}f\left( \tfrac{\textrm{d}_{F}}{\tau }\right) dy - \int _{F_\tau \Delta F}f\left( \tfrac{\textrm{d}_{F}}{\tau }\right) dy \\  &   \quad = -\int _{B_r\cap (F_\tau ^c\setminus F^c)} f\left( \tfrac{\textrm{d}_{F}}{\tau }\right) dy + \int _{B_r\cap F_\tau ^c\cap F^c} f\left( \tfrac{\textrm{d}_{F}}{\tau }\right) dy \le \int _{B_r\cap F_\tau ^c\cap F^c} f\left( \tfrac{\textrm{d}_{F}}{\tau }\right) dy. \end{aligned}$$

Note that if $x\in \partial F_\tau$ and $y\in B_r\cap F_\tau \cap F$ (resp. $y\in B_r\cap F_\tau ^c\cap F^c$), by the 1-lipschitzianity of $\textrm{d}_F$ and (3.10)

$$\begin{aligned} \textrm{d}_F(y) \le \textrm{d}_F(x) + |y-x| \le 2\rho _\tau + r, \end{aligned}$$

and hence, by the monotonicity of $f$

$$\begin{aligned} \int _{B_r\cap F_\tau \cap F} f\left( \tfrac{\textrm{d}_{F}}{\tau }\right) dy \le f\Big (\tfrac{r+2\rho _\tau }{\tau }\Big ) |B_r\cap F_\tau | \end{aligned}$$

and similarly

$$\begin{aligned} \int _{B_r\cap F_\tau ^c\cap F^c} f\left( \tfrac{\textrm{d}_{F}}{\tau }\right) dy \le f\Big (\tfrac{r+2\rho _\tau }{\tau }\Big ) |B_r\cap F_\tau ^c|. \end{aligned}$$

For any $\tau \in (0,\tau _0)$ let $r_\tau >0$ be the unique number (compare (1b)) satisfying

3.11 $$\begin{aligned} r_\tau f\Big (\tfrac{r_\tau + 2\rho _\tau }{\tau }\Big ) = \frac{c_{\varphi }n}{2}. \end{aligned}$$

By the continuity of $f,$ $\tau \mapsto r_\tau$ is continuous and $r_\tau \rightarrow 0$ as $\tau \rightarrow 0^+.$

Possibly decreasing $\tau _0$ in (3.9), we assume

3.12 $$\begin{aligned} r_\tau <\gamma _g\qquad \mathrm{for~ all}~ \tau \in (0,\tau _0). \end{aligned}$$

Now, by the increasing monotonicity of $r\mapsto rf((r+2\rho _\tau )/\tau )$ and the definition of $r_\tau$, for any $r\in (0,r_\tau ]$ we have

$$\begin{aligned} f\Big (\tfrac{r+2\rho _\tau }{\tau }\Big ) |B_r\cap F_\tau | = \omega _n^{1/n} r f\Big (\tfrac{r+2\rho _\tau }{\tau }\Big )|B_r\cap F_\tau |^{\frac{n-1}{n}} \le \frac{c_{\varphi }n\omega _n^{1/n}}{2}|B_r\cap F_\tau |^{\frac{n-1}{n}} \end{aligned}$$

and

$$\begin{aligned} f\Big (\tfrac{r+2\rho _\tau }{\tau }\Big ) |B_r\cap F_\tau ^c| = \omega _n^{1/n}rf\Big (\tfrac{r+2\rho _\tau }{\tau }\Big )|B_r\cap F_\tau ^c|^{\frac{n-1}{n}} \le \frac{c_{\varphi }n\omega _n^{1/n}}{2}|B_r\cap F_\tau ^c|^{\frac{n-1}{n}}. \end{aligned}$$

Recalling the inequality $r_\tau <\gamma _g$ and the estimates (3.2) and (3.3), for a.e. $r\in (0,r_\tau ]$ we have

$$\begin{aligned}  &   0\le {\mathcal {F}}(F_\tau \setminus B_r;F,\tau ) - {\mathcal {F}}(F_\tau ;F,\tau ) \le 2C_{\varphi }\mathcal {H}^{n-1}(F_\tau \cap B_r) \\  &   \quad - c_{\varphi }n\omega _n^{1/n}|F_\tau \cap B_r|^{\frac{n-1}{n}} + \frac{c_{\varphi }n\omega _n^{1/n}}{2}|F_\tau \cap B_r|^{\frac{n-1}{n}} + \frac{c_{\varphi }n\omega _n^{1/n}}{2}|F_\tau \cap B_r|^{\frac{n-1}{n}}. \end{aligned}$$

This implies, similarly to (3.5),

3.13 $$\begin{aligned} |F_\tau \cap B_r|\ge \Big (\tfrac{3c_{\varphi }}{8C_{\varphi }}\Big )^n\omega _n r^n,\quad r\in (0,r_\tau ]. \end{aligned}$$

Analogously,

3.14 $$\begin{aligned} |F_\tau ^c\cap B_r|\ge \Big (\tfrac{3c_{\varphi }}{8C_{\varphi }}\Big )^n\omega _n r^n,\quad r\in (0,r_\tau ]. \end{aligned}$$

From (3.13), (3.14), and the relative isoperimetric inequality in balls, we obtain

3.15 $$\begin{aligned} P(F_\tau ,B_r) \ge \theta _1r^{n-1},\quad \tau \in (0,\tau _0), \quad r\in (0,r_\tau ), \end{aligned}$$

with $$\theta _1:= 2^{\frac{n-1}{n}}\omega _{n-1} \Big (\tfrac{c_{\varphi }}{8C_{\varphi }}\Big )^{n-1}$$.

### Existence of GMM

As already mentioned in the introduction, the proof runs along a well-established path due to Almgren–Taylor–Wang [2] and Luckhaus-Sturzenhecker [25]; however, here various nontrivial technical modifications are required mainly due to the presence of $f$ (and of $g$).

Let us assume Hypothesis (H). Fix $E_0\in \mathcal {S}^*,$ and $\tau \in (0,\tau _0)$ (recall that $\tau _0$, given in (3.9) and (3.12)), and using Lemma 3.1 define the flat flows $\{E(\tau ,k)\}_{k\ge 0}$ inductively as follows: $E(\tau ,0)= E_0$ and

$$\begin{aligned} E(\tau ,k) \in \textrm{argmin}\, {\mathcal {F}}(\cdot ; E(\tau ,k-1),\tau ),\quad k\ge 1. \end{aligned}$$

From the inequality

$$\begin{aligned} {\mathcal {F}}(E(\tau ,k);E(\tau ,k-1),\tau ) \le {\mathcal {F}}(E(\tau ,k-1);E(\tau ,k-1),\tau ),\quad k\ge 1, \end{aligned}$$

we get the standard estimate

3.16 $$\begin{aligned} \int _{E(\tau ,k)\Delta E(\tau ,k-1)} f\left( \tfrac{\textrm{d}_{E(\tau ,k-1)}}{\tau }\right) ~dx \le {{\mathfrak {p}}}_{k-1} - {{\mathfrak {p}}}_k,\quad k\ge 1, \end{aligned}$$

where

$$\begin{aligned} {{\mathfrak {p}}}_k:= P_\varphi (E(\tau ,k)) + \int _{E(\tau ,k)} g~dx,\quad k\ge 0, \end{aligned}$$

and hence the sequence $({{\mathfrak {p}}}_k)$ is nonincreasing. In particular,

$$\begin{aligned} P_\varphi (E(\tau ,k)) = {{\mathfrak {p}}}_k - \int _{E(\tau ,k)} g~dx \le {{\mathfrak {p}}}_0 + \int _{E(\tau ,k)} |g| ~dx \end{aligned}$$

and therefore

3.17 $$\begin{aligned} P_\varphi (E(\tau ,k)) \le {{\mathfrak {p}}}_0+\int _{{\mathbb {R}}^n} |g| ~dx:=C_2\quad \hbox { for all}\ k\ge 0. \end{aligned}$$

Consider the family $\{E(\tau ,\left\lfloor t/\tau \right\rfloor )\}_{t\ge 0}.$ In view of (3.17), compactness in $BV_\textrm{loc}({\mathbb {R}}^n;\{0,1\})$ and a diagonal argument, we can find a sequence $\tau _j\rightarrow 0^+$ such that for every rational $t\ge 0$ there exists a set $E(t)\in BV_\textrm{loc}({\mathbb {R}}^n;\{0,1\})$ such that

3.18 $$\begin{aligned} E(\tau _j,\left\lfloor t/\tau _j \right\rfloor ) \rightarrow E(t)\quad \text {in }L_\textrm{loc}^1({\mathbb {R}}^n)\hbox { as} j\rightarrow +\infty . \end{aligned}$$

In view of (3.17) and the isoperimetric inequality (2.2), the measure of $E(\tau _j,\left\lfloor t/\tau _j \right\rfloor )$ is bounded uniformly in *j* and *t*,  and therefore |*E*(*t*)| is bounded for each rational $t\ge 0$, in particular $E(t)\in \mathcal {S}^*.$

Now we will prove that the convergence (3.18) holds for any $t\ge 0$ (without passing to a further subsequence). To this aim, let us establish a sort of uniform continuity of flat flows $\{E(\tau ,k)\}$.

In view of (3.15), each $E(\tau ,k)$ with $k\ge 1$ satisfies a uniform lower perimeter density estimate for radii $r\in (0,r_\tau ]$. Thus, we can apply Lemma A.1 with

3.19 $$\begin{aligned} r_0=r_\tau \end{aligned}$$

and $\vartheta =\theta _1$, to estimate the measure $\vert E(\tau ,k-1)\Delta E(\tau ,k)\vert .$ In view of the expression of the “distance”-term in ${\mathcal {F}},$ we apply that lemma with

3.20 $$\begin{aligned} \ell =\tau \end{aligned}$$

and some $p>0$ to be chosen later (see (3.23)).

#### Remark 3.2

(*Necessity of assumption *(1.9)) Assumption (1.9) implies

$$\begin{aligned} \limsup _{\tau \rightarrow 0^+} \frac{\tau }{r_\tau } \in [0,+\infty ). \end{aligned}$$

If this limsup is infinite (for instance, in the case $f(r)=e^r$ for large *r*,  see Example 2.2), an application of Lemma A.1 would give a large coefficient $\frac{\tau ^{n-1}}{r_\tau ^{n-1}}$ in the first inequality in estimate (A.2), which seems hard to handle. To avoid such an issue, we assume the validity of (1.9), which is used only here.

For any $p>0$ and small $\tau >0$ we have, by (3.19), (3.20), and (A.2),

$$\begin{aligned} |E(\tau ,k)\Delta E(\tau ,k-1)| \le C_3 p^\sigma \tau P_\varphi (E(\tau ,k-1)) + \frac{1}{f(p)}\int _{E(\tau ,k)\Delta E(\tau ,k-1)} f\left( \tfrac{\textrm{d}_{E(\tau ,k-1)}}{\tau }\right) ~dx \end{aligned}$$

for all $k\ge 2,$ where $C_3>0$ and $\sigma \in \{1,n\}.$ By the uniform perimeter bound (3.17) and inequality (3.16) we can estimate further

$$\begin{aligned} |E(\tau ,k)\Delta E(\tau ,k-1)| \le C_4 p^\sigma \tau + \frac{{{\mathfrak {p}}}_{k-1} - {{\mathfrak {p}}}_k}{f(p)}, \end{aligned}$$

where $C_4:=C_2C_3.$ Summing these inequalities in $k=m_1+1,\ldots ,m_2$ for $1\le m_1<m_2$ we obtain

3.21 $$\begin{aligned} |E(\tau ,m_2)\Delta E(\tau ,m_1)| \le C_4 p^\sigma \tau (m_2-m_1) + \frac{{{\mathfrak {p}}}_{m_1} - {{\mathfrak {p}}}_{m_2}}{f(p)}. \end{aligned}$$

Since

$$\begin{aligned} {{\mathfrak {p}}}_{m_1} - {{\mathfrak {p}}}_{m_2} \le&{{\mathfrak {p}}}_0 - {{\mathfrak {p}}}_{m_2} \le {{\mathfrak {p}}}_0 + \int _{E(\tau ,m_2)} g^-(x)~dx \le C_2, \end{aligned}$$

(3.21) estimates further as

3.22 $$\begin{aligned} |E(\tau ,m_1) \Delta E(\tau ,m_2)| \le C_4 p^\sigma (m_2-m_1)\tau + \frac{C_2}{f(p)}. \end{aligned}$$

Now we come back to our chosen flat flows $\{E(\tau _j,\left\lfloor t/\tau _j \right\rfloor )\}_{t\ge 0}.$ Fix any $t_2>t_1>0$ and, for $\tau _0$ as in (3.9), (3.12), let $\tau _j\in (0,\tau _0)$ be so small that $\min \{t_1,t_2-t_1\}>10\tau _j.$ Now we choose *p*. Let $u:\mathbb {R}_0^+\rightarrow \mathbb {R}_0^+$ be any continuous increasing function^6^ with $u(0)=0$ and

$$\begin{aligned} \limsup \limits _{s\rightarrow 0^+} \frac{s}{u(s)} =0. \end{aligned}$$

We set

3.23 $$\begin{aligned} p: = {\left\{ \begin{array}{ll} \frac{1}{u(t_2-t_1)^{1/n}} &  \text {if } \liminf \limits _{j\rightarrow +\infty }\frac{r_{\tau _j}}{\tau _j} \in (0, +\infty ), \\ \frac{1}{u(t_2-t_1)} &  \text {if }\liminf \limits _{j\rightarrow +\infty }\frac{r_{\tau _j}}{\tau _j}=+\infty . \end{array}\right. } \end{aligned}$$

Applying (3.22) with such a *p*,  $m_1=\left\lfloor t_1/\tau _j \right\rfloor ,$ $m_2=\left\lfloor t_2/\tau _j \right\rfloor$, using $\tau _j(m_2-m_1) \le t_2-t_1+\tau _j$ and recalling $p^\sigma =1/u(t_2-t_1),$ we get

3.24 $$\begin{aligned} |E(\tau _j,\left\lfloor t_2/\tau _j \right\rfloor ) \Delta E(\tau _j,\left\lfloor t_1/\tau _j \right\rfloor )| \le \omega (t_2-t_1) + \frac{C_4\tau _j}{u(t_2-t_1)}, \end{aligned}$$

where, for $$s>0$$,

3.25 $$\begin{aligned} \omega (s)= {\left\{ \begin{array}{ll} \frac{C_4s}{u(s)} + \frac{C_2}{f(u(s)^{-1/n})} &  \text {if } \liminf \limits _{j\rightarrow +\infty }\frac{r_{\tau _j}}{\tau _j} \in (0, +\infty ), \\ \frac{C_4s}{u(s)} + \frac{C_2}{f(u(s)^{-1})} &  \text {if } \liminf \limits _{j\rightarrow +\infty }\frac{r_{\tau _j}}{\tau _j}=+\infty . \end{array}\right. } \end{aligned}$$

Now if both $t_1$ and $t_2$ are rational, then letting $j\rightarrow +\infty$ in (3.24) and recalling (3.18), we deduce

3.26 $$\begin{aligned} |E(t_2)\Delta E(t_1)| \le \omega (t_2-t_1). \end{aligned}$$

By assumptions on *u* and $f,$ $\omega (s)\rightarrow 0$ as $s\rightarrow 0^+.$ Thus, $\omega$ provides a modulus of continuity, which is for the moment defined only for rational times. Since

$$\begin{aligned} \sup _{t\ge 0,\,\text {rational}}\,\,\, P_\varphi (E(t))\le C_2, \end{aligned}$$

by standard arguments, we can uniquely extend $E(\cdot )$ for all $t\ge 0$ still satisfying the uniform continuity (3.26) for all $0<t_1<t_2.$

We claim that $E(\cdot )$ is a GMM, i.e., the convergence (3.18) holds for all $t\ge 0$. Indeed, consider an irrational *t*,  and take any rational ${\bar{t}}>t.$ Then for any open set $U\subset {\mathbb {R}}^n,$

$$\begin{aligned}  &   |U\cap (E(\tau _j,\left\lfloor t/\tau _j \right\rfloor ) \Delta E(t))| \le |U\cap (E(\tau _j,\left\lfloor t/\tau _j \right\rfloor ) \Delta U\cap (E(\tau _j,\left\lfloor {\bar{t}}/\tau _j \right\rfloor ))| \\  &   \quad + |U\cap (E(\tau _j,\left\lfloor {\bar{t}}/\tau _j \right\rfloor ) \Delta E(\bar{t}))| + |E({\bar{t}})\Delta E(t)|=: a_j+b_j+c. \end{aligned}$$

Since ${\bar{t}}$ is rational, by (3.18)

$$\begin{aligned} \limsup _{j\rightarrow +\infty } b_j = 0. \end{aligned}$$

Moreover, by (3.24) and (3.26)

$$\begin{aligned} \limsup _{j\rightarrow +\infty } a_j \le \omega ({\bar{t}}- t)\quad \text {and}\quad c\le \omega ({\bar{t}}-t). \end{aligned}$$

Therefore,

$$\begin{aligned} \limsup _{j\rightarrow +\infty }\,|U\cap (E(\tau _j,\left\lfloor t/\tau _j \right\rfloor ) \Delta E(t))| \le 2 \omega ({\bar{t}}-t). \end{aligned}$$

Now, letting ${\bar{t}}\searrow t$ and recalling that $\omega ({\bar{t}}-t)\rightarrow 0$, we deduce the validity of (3.18) for all times $t\ge 0.$ Whence, by definition, $E(\cdot )$ is a GMM.

#### Remark 3.3

In the proof of the existence we started with an arbitrary sequence and constructed a subsequence for which the $L_\textrm{loc}^1$-convergence (3.18) holds.

Finally, let us prove that every $\{E(t)\}_{t\ge 0}\in \textrm{GMM}({\mathcal {F}},\mathcal {S}^*,E_0)$ starting from $E_0$ is uniformly continuous. Let $\{E(t)\}$ be defined as an $L_\textrm{loc}^1$-limit of flat flows $\{E(\tau _j,\left\lfloor t/\tau _j \right\rfloor )\}$ for some $\tau _j\rightarrow 0.$ Then these flat flows necessarily satisfy (3.24) and hence, after passing to the limit, we see that $E(\cdot )$ also satisfies (3.26).

### Uniform Continuity of GMM up to $t=0$

Let us assume that $|\partial F| = 0$ and let $F_\tau$ be a minimizer of ${\mathcal {F}}(\cdot ;F,\tau ).$ By minimality we get

3.27 $$\begin{aligned} P_\varphi (F_\tau ) + \int _{F_\tau \Delta F_0}f\left( \tfrac{\textrm{d}_{F}}{\tau }\right) ~dx + \int _{F_\tau } g~dx \le P_\varphi (F) + \int _{F} g~dx, \end{aligned}$$

and hence, recalling $g\in L^1({\mathbb {R}}^n),$ the family $\{P_\varphi (F_\tau )\}$ is uniformly bounded with respect to $\tau$. By compactness, for any sequence $\tau _k\searrow 0,$ $F_{\tau _k}\overset{L_\textrm{loc}^1}{\rightarrow }\ F_0$ (up to a not relabelled subsequence) where $F_0\in \mathcal {S}^*.$ By (3.27), the assumption $|\partial F| = 0,$ and Fatou’s lemma, $|F_0\Delta F|=0,$ and hence, $F_0=F.$ Thus,

3.28 $$\begin{aligned} F_\tau \rightarrow F \end{aligned}$$

in $L_\textrm{loc}^1({\mathbb {R}}^n)$ as $\tau \rightarrow 0.$ Of course, using (3.27) one can also show

$$\begin{aligned} \lim \limits _{\tau \rightarrow 0} P_\varphi (F_\tau ) = P_\varphi (F)\quad \text {and}\quad \lim \limits _{\tau \rightarrow 0} \int _{F_\tau \Delta F_0}f\left( \tfrac{\textrm{d}_{F}}{\tau }\right) dx = 0. \end{aligned}$$

Now, to prove the uniform continuity of GMM for $s,t\ge 0$ we proceed as in the standard Almgren–Taylor–Wang case: applying (3.24) with $t_2=t>0$ and $t_1=\tau _j,$ as well as (3.28) for any open set $U\subset {\mathbb {R}}^n$, we find

$$\begin{aligned}  &   |U\cap (E(\tau _j,\left\lfloor t/\tau _j \right\rfloor ) \Delta E_0)| \le |E(\tau _j,\left\lfloor t/\tau _j \right\rfloor ) \Delta E(\tau _j,1)| +\\  &   |U\cap (E(\tau _j,1)\Delta E_0)| \le \omega (t- \tau _j) + o(1) \end{aligned}$$

as $j\rightarrow +\infty .$ This implies $|U\cap (E(t)\Delta E_0)| \le \omega (t)$, and hence, letting $U\rightarrow {\mathbb {R}}^n$ we get $|E(t)\Delta E_0| \le \omega (t).$

### Hölder Continuity of GMM

Suppose

3.29 $$\begin{aligned} \lim _{r\rightarrow +\infty }\,\frac{f(r)}{r^\alpha } = C_5 \in (0,+\infty ) \end{aligned}$$

for some $\alpha \in (0,+\infty ).$ Then using (3.8) and recalling $\rho _\tau /\tau \rightarrow +\infty$ as $\tau \rightarrow 0^+$ (see (2.4)) we get

$$\begin{aligned} \limsup _{\tau \rightarrow 0^+} \frac{C_1\tau ^\alpha }{\rho _\tau ^{1+\alpha }} = \limsup _{\tau \rightarrow 0^+}\frac{f(\rho _\tau /\tau )}{(\rho _\tau /\tau )^\alpha } = C_5, \end{aligned}$$

and hence,

$$\begin{aligned} \liminf \limits _{\tau \rightarrow 0^+}\frac{\rho _\tau }{\tau ^{\frac{\alpha }{1+\alpha }}} = \frac{C_1^{\frac{1}{1+ \alpha }}}{C_5}. \end{aligned}$$

Similarly, as $\frac{r_\tau + 2\rho _\tau }{\tau }\rightarrow +\infty$ (see Example 2.1), by (3.11) we obtain

$$\begin{aligned} C_5 = \limsup _{\tau \rightarrow 0^+}\,\frac{f((r_\tau + 2\rho _\tau )/\tau )}{[(r_\tau + 2\rho _\tau )/\tau ]^\alpha } = \limsup _{\tau \rightarrow 0^+}\,\frac{c_{\varphi }n}{2r_\tau [(r_\tau + 2\rho _\tau )/\tau ]^\alpha }. \end{aligned}$$

Thus,

$$\begin{aligned} \Big (\tfrac{2C_5}{c_{\varphi }n}\Big )^{1/\alpha } = \liminf _{\tau \rightarrow 0} \Big (\frac{r_\tau ^{\frac{\alpha +1}{\alpha }}}{\tau } + \frac{2r_\tau ^{\frac{1}{\alpha }}\rho _\tau }{\tau }\Big ), \end{aligned}$$

and therefore

$$\begin{aligned} C_6:=\liminf \limits _{\tau \rightarrow 0^+}\frac{r_\tau }{\tau ^{\frac{ \alpha }{1+\alpha }}}<+\infty . \end{aligned}$$

This implies

$$\begin{aligned} \liminf \limits _{\tau \rightarrow 0^+}\frac{r_\tau }{\tau }=+\infty . \end{aligned}$$

Now choosing $u(s) = s^\frac{1}{1+\alpha }$, in (3.22) we can represent the function $\omega$ in (3.26) as

3.30 $$\begin{aligned} \omega (s) = C_4s^{\frac{\alpha }{1+\alpha }} + \frac{C_2}{f(s^{-\frac{1}{1+\alpha }})}. \end{aligned}$$

In view of (3.29) and the strict monotonicity of $f,$ there exists $s_0>0$ such that

$$\begin{aligned} f(s^{-\frac{1}{1+\alpha }}) \ge \frac{C_5}{2}\,s^{-\frac{\alpha }{1+\alpha }}\quad \hbox { for all}\ s\in (0,s_0). \end{aligned}$$

Thus, for such *s*,  from (3.30) we conclude

$$\begin{aligned} \omega (s) \le \Big (C_4 + \frac{2C_2}{C_5}\Big )s^{\frac{\alpha }{1+\alpha }}. \end{aligned}$$

In view of (3.26) this implies the local $\frac{\alpha }{1+\alpha }$-continuity of GMM.

### Boundedness of $\textrm{GMM}$

The next lemma shows that boundedness of an initial set implies boundedness of minimizers of ${\mathcal {F}}$.

#### Lemma 3.4

Suppose Hypothesis (H). Then, for any bounded $F\in \mathcal {S}^*$ and any $\tau >0$, every minimizer of ${\mathcal {F}}(\cdot ;F,\tau )$ is bounded.

#### Proof

Let *E* be a minimizer of ${\mathcal {F}}(\cdot ;F,\tau )$ and let $\gamma _g>0$ be given by (A.3). Writing $B_r:=B_r(0),$ let $r_0>0$ be such that $F\subset B_{r_0}$ and $|E{\setminus } B_r|<\omega _n \gamma _g^n$ for all $r>r_0$ (the last inequality is possible since $|E|<+\infty$). For any $r>r_0,$ by the minimality of *E* and the inequality $F{\setminus } E = F{\setminus } [E\cap B_r]$, we have

3.31 $$\begin{aligned}  &   0\le {\mathcal {F}}(E\cap B_r;F,\tau ) - {\mathcal {F}}(E;F,\tau ) \nonumber \\  &   \quad = P_\varphi (E\cap B_r) - P_\varphi (E) - \int _{E\setminus B_r} f\left( \tfrac{\textrm{d}_{F}}{\tau }\right) dx - \int _{E\setminus B_r} gdx. \end{aligned}$$

By the standard properties of the reduced boundary, (2.1) and the Euclidean isoperimetric inequality, we find

$$\begin{aligned}  &   P_\varphi (E\cap B_r) - P_\varphi (E) = \int _{E\cap \partial B_r} \varphi ^o(\nu _{B_r}) d\mathcal {H}^{n-1} - P_\varphi (E,B_r^c)\\  &   \quad = \int _{E\cap \partial B_r} \Big [\varphi ^o(\nu _{B_r})+\varphi ^o(-\nu _{B_r})\Big ] d\mathcal {H}^{n-1}- P_\varphi (E\setminus B_r) \le 2C_{\varphi }\mathcal {H}^{n-1}(E\cap \partial B_r) \\  &   \quad - c_{\varphi }n\omega _n^{1/n} |E\setminus B_r| \end{aligned}$$

for a.e. $r>r_0.$ Moreover, since $\textrm{d}_F \ge r-r_0$ in $E{\setminus } B_r$ and $f$ is increasing,

$$\begin{aligned} f\left( \tfrac{\textrm{d}_{F}}{\tau }\right) \ge f\Big (\tfrac{r-r_0}{\tau }\Big )\quad \text {a.e. in }E\setminus B_r \end{aligned}$$

and therefore

$$\begin{aligned} \int _{E\setminus B_r} f\left( \tfrac{\textrm{d}_{F}}{\tau }\right) dx \ge f\Big (\tfrac{r-r_0}{\tau }\Big ) |E\setminus B_r|. \end{aligned}$$

By the choice of $r_0$ and (A.3)

$$\begin{aligned} - \int _{E\setminus B_r} gdx \le \tfrac{c_{\varphi }n\omega _n^{1/n}}{4}|E\setminus B_r|^{\frac{n-1}{n}}. \end{aligned}$$

Inserting the above estimates in (3.31) we find

$$\begin{aligned} 2 C_{\varphi }\mathcal {H}^{n-1}(E\cap B_r) + \tfrac{c_{\varphi }n\omega _n^{1/n}}{4}|E\setminus B_r|^{\frac{n-1}{n}} \ge c_{\varphi }n\omega _n^{1/n}|E\setminus B_r|^{\frac{n-1}{n}} + f\Big (\tfrac{r-r_0}{\tau }\Big ) |E\setminus B_r|. \end{aligned}$$

In particular,

$$\begin{aligned} \mathcal {H}^{n-1}(E\cap B_r^c) \ge \tfrac{3c_{\varphi }n\omega _n^{1/n}}{8C_{\varphi }} |E\cap B_r^c|^{\frac{n-1}{n}}\quad \text {for a.e. }r>r_0. \end{aligned}$$

If $|E\cap B_r^c|>0$ for all $r>0,$ then integrating this differential inequality in $(r_0,r)$ we get

$$\begin{aligned} \tfrac{3c_{\varphi }}{8C_{\varphi }}\,(r-r_0) \le |E\cap B_{r_0}^c|^{1/n} - |E\cap B_{r}^c|^{1/n}. \end{aligned}$$

However, letting $r\rightarrow +\infty$ and using $|E|<+\infty ,$ we obtain $|E\cap B_{r_0}^c|=+\infty ,$ a contradiction. Thus, $|E\cap B_r^c|=0$ for some $r>r_0,$ i.e., $E\subset B_r.$ $\square$

From this lemma, we deduce that each flat flow starting from a bounded set is bounded. However, when passing to the limit as $\tau _j\rightarrow 0,$ we may loose this boundedness, and therefore we need this lemma in some stronger form and for this we need stronger assumptions on $g.$ In what follows we write, for $\varrho >0$ and $x \in {\mathbb {R}}^n$,

$$\begin{aligned} W_\varrho (x) = \{\xi \in {\mathbb {R}}^n: \varphi (\xi -x) < \varrho \}, \qquad W_\varrho = W_\varrho (0). \end{aligned}$$

#### Lemma 3.5

(**Growth of **$g^-$ **controlled by** $f$) Assume Hypothesis (H) and that $g$ satisfies (1.11). Then there exist constants $C_6,C_7>0$ depending only on $c_{g^-},c_{\varphi },C_{\varphi }$ and $\gamma _g$ (see (A.3)) such that the following holds. For any $F\in \mathcal {S}^*$ and $\tau >0$ let $F_\tau$ be a minimizer of ${\mathcal {F}}(\cdot ;F,\tau ).$ Suppose that $F\subset W_{r_0}$ for some $r_0>C_6.$ Then $F_\tau \subset W_{r_\tau }$ for any $\tau < \frac{1}{2C_7},$ where $r_\tau = (1+C_7\tau )r_0+ C_7\tau .$

#### Proof

By (2.1), (1.11) and the strict monotonicity of $f$,

3.32 $$\begin{aligned} g^-(x) \le f\Big ( c_{g^-}+ \tfrac{c_{g^-}}{c_{\varphi }}\varphi (x)\Big ),\quad \varphi (x)>\tfrac{c_{g^-}}{c_{\varphi }}. \end{aligned}$$

Let $F\subset W_{r_0}$ for some $r_0>C_6:=c_{g^-}/c_{\varphi };$ by Lemma 3.4 a minimizer $F_\tau$ is bounded, and let $W_{r_\tau }$ be the smallest Wulff shape containing $F_\tau .$ Thus, $\partial F_\tau \cap \partial W_{r_\tau } \ne \emptyset .$ We may assume $r_\tau >r_0.$

Fix a small $\epsilon \in (0, r_\tau -r_0)$ and consider the difference

3.33 $$\begin{aligned}  &   0\le {\mathcal {F}}(F_\tau \cap W_{r_\tau -\epsilon }; F,\tau ) - {\mathcal {F}}(F_\tau ;F,\tau ) = P_\varphi (F_\tau \cap W_{r_\tau -\epsilon }) - P_\varphi (F_\tau )\nonumber \\  &   \qquad - \int _{F_\tau \setminus W_{r_\tau }} f\left( \tfrac{\textrm{d}_{F}}{,\tau }\right) dx -\int _{F_\tau \setminus W_{r_\tau -\epsilon }} g\,dx. \end{aligned}$$

By (2.1), $\textrm{d}_{F}(x) \ge \frac{1}{C_{\varphi }}\textrm{d}_{F}^\varphi (x) \ge \frac{r_\tau -\epsilon -r_0}{C_{\varphi }}$ in $F_\tau {\setminus } W_{r_\tau - \epsilon },$ where

$$\begin{aligned} \textrm{d}_{F}^\varphi (x):=\inf \{\varphi (x-y):\,\,y\in \partial ^* F\} \end{aligned}$$

is the anisotropic distance. Thus, by the monotonicity of $f,$

$$\begin{aligned} -\int _{F_\tau \setminus W_{r_\tau }} f\left( \tfrac{\textrm{d}_{F}}{\tau }\right) dx \le -f\Big (\frac{r_\tau -\epsilon -r_0}{C_{\varphi }\tau }\Big )\,|F_\tau \setminus W_{r_\tau -\epsilon }|. \end{aligned}$$

In view of (3.32), using $r_0<r_\tau - \epsilon <\varphi (\cdot )\le r_\tau$ in $E{\setminus } W_{r_\tau -\epsilon }$ and assumption (3.32) we have

$$\begin{aligned}  &   - \int _{F_\tau \setminus W_{r_\tau - \epsilon }} gdx \le \int _{F_\tau \setminus W_{r_\tau -\epsilon }} g^- dx \\  &   \quad \le \int _{F_\tau \setminus W_{r_\tau -\epsilon }} f\Big (c_{g^-}+ \tfrac{c_{g^-}}{c_{\varphi }}\varphi (x)\Big )dx \le f\Big (c_{g^-}+ \tfrac{c_{g^-}}{c_{\varphi }}r_\tau \Big )|E\setminus W_{r_\tau - \epsilon }|. \end{aligned}$$

Finally, by the convexity of $W_{r_\tau -\epsilon },$

$$\begin{aligned} P_\varphi (F_\tau \cap W_{r_\tau -\epsilon }) - P_\varphi (F_\tau ) \le 0. \end{aligned}$$

Inserting these estimates in (3.33) we get

$$\begin{aligned} 0\le -f\Big (\frac{r_\tau -\epsilon -r_0}{C_{\varphi }\tau }\Big ) + f\Big (c_{g^-}+ \tfrac{c_{g^-}}{c_{\varphi }}r_\tau \Big ), \end{aligned}$$

thus, by the strict monotonicity of $f$ and the arbitrariness of $\epsilon$ we obtain

$$\begin{aligned} \frac{r_\tau - r_0}{C_{\varphi }\tau }\le c_{g^-}+ \frac{c_{g^-}}{c_{\varphi }}r_\tau . \end{aligned}$$

Now, assuming $\frac{c_{g^-}C_{\varphi }}{c_{\varphi }}\tau <\frac{1}{2}$ we can write

$$\begin{aligned} r_\tau \le \frac{r_0 + c_{g^-}C_{\varphi }\tau }{1 - \frac{c_{g^-}C_{\varphi }}{c_{\varphi }}\tau } \le \Big (1 + \frac{2c_{g^-}C_{\varphi }}{c_{\varphi }}\tau \Big )r_0 + c_{g^-}C_{\varphi }\tau \Big (1 + \frac{2c_{g^-}C_{\varphi }}{c_{\varphi }}\tau \Big )\le (1+C_7\tau )r_0 + C_7\tau , \end{aligned}$$

where $$C_7:=\max \{\frac{2c_{g^-}C_{\varphi }}{c_{\varphi }}, 2c_{g^-}C_{\varphi }\}.$$ $$\square $$

Now, we prove the local uniform boundedness of GMM, as stated in (ii). Fix $\tau \in (0,\frac{1}{2C_7})$ and a bounded $E_0\in \mathcal {S}^*,$ and let $\{E(\tau ,k)\}$ be a flat flow starting from $E_0.$ We may assume $E_0\subset W_{r_0}$ for some $r_0>C_6.$ Let $\{r(\tau ,k)\}_k$ be a nondecreasing sequence such that $r(\tau ,0)=r_0,$ $E(\tau ,k)\subset W_{r(\tau ,k)}$ and for any $k\ge 1$ either $r(\tau ,k)=r(\tau ,k-1),$ or by Lemma 3.5$r(\tau ,k)\le (1+C_7\tau )r(\tau ,k-1) + C_7\tau .$ Thus, there is no loss of generality in assuming

$$\begin{aligned} r(\tau ,k)\le (1+C_7\tau )r(\tau ,k-1) + C_7\tau ,\quad k\ge 1. \end{aligned}$$

Then^7^

$$\begin{aligned} r(\tau ,k) \le (1+C_7\tau )^kr_0 + C_7\tau \,\frac{(1+C_7\tau )^k-1}{C_7\tau } < (1+C_7\tau )^k(r_0+1). \end{aligned}$$

Therefore, for any $t\ge 0$,

$$\begin{aligned} r(\tau ,\left\lfloor t/\tau \right\rfloor ) \le (1+C_7\tau )^{\left\lfloor t/\tau \right\rfloor }(r_0+1) \le (1+C_7\tau )^{t/\tau }(r_0+1) \le e^{C_7t}(r_0+1) = :R(t). \end{aligned}$$

This implies $E(\tau ,\left\lfloor t/\tau \right\rfloor )\subset W_{R(t)}$ for all $t\ge 0$ and therefore for any $E(\cdot )\in \textrm{GMM}({\mathcal {F}},\mathcal {S}^*,E_0)$ we have $E(t)\subset W_{R(t)}.$

## Rescalings of $\textrm{GMM}$ and Comparison with Balls

Let us study how minimizers are related in rescaling.

### Lemma 4.1

(Rescaling) Assume that $f$ satisfies (1a) and (1b), and $g\equiv 0.$ Suppose that $E_0\in \mathcal {S}^*$ contains the origin in its interior and $\lambda >0$. Then $\lambda E(\cdot ) \in \textrm{GMM}({\mathcal {F}}, \mathcal {S}^*,\lambda E_0)$ if and only if $E(\cdot ) \in \textrm{GMM}(\mathcal {F}_{\varphi , \lambda f, 0},\mathcal {S}^*,E_0).$

### Proof

As usual, ${\mathcal {F}}= \mathcal {F}_{\varphi ,f,0}$. Since

4.1 $$\begin{aligned}  &   {\mathcal {F}}(\lambda G;\lambda E_0,\tau ) = P_\varphi (\lambda G) + \int _{\lambda G}f\left( \tfrac{\textrm{sd}_{\lambda E_0}}{\tau }\right) ~dx \nonumber \\  &   \quad = \lambda ^{n-1}P_\varphi (G) + \lambda ^n\int _{G}f\left( \tfrac{\textrm{sd}_{E_0}}{\tau }\right) ~dx = \lambda ^{n-1}\mathcal {F}_{\varphi ,\lambda f,0} (G;E_0,\tau ), \end{aligned}$$

$\lambda G$ is a minimizer of ${\mathcal {F}}(\cdot ;\lambda E_0,\tau )$ if and only if *G* is a minimizer of $\mathcal {F}_{\varphi ,\lambda f,0} (\cdot ;E_0,\tau ).$

Let $\{\lambda E(\tau ,k)\}$ be flat flows starting from $\lambda E_0,$ associated to ${\mathcal {F}}.$ Then by (4.1), the family $\{E(\tau ,k)\}$ is a flat flow starting from $E_0,$ associated to $\mathcal {F}_{\varphi ,\lambda f,0}.$ This implies the thesis. $\square$

### Corollary 4.2

(Power case) Assume that $g\equiv 0$,

$$\begin{aligned} f(r) = r^\alpha ,\quad r\ge 0, \end{aligned}$$

for some $\alpha >0,$ and $E_0\in \mathcal {S}^*$ contains the origin in its interior. Then $E(\cdot ) \in \textrm{GMM}({\mathcal {F}},\mathcal {S}^*,E_0)$ if and only if $\lambda E(t\lambda ^{-1/\alpha }) \in \textrm{GMM}({\mathcal {F}},\mathcal {S}^*, \lambda E_0).$

### Proof

Since $\lambda f(r) = f(\lambda ^{1/\alpha } r),$ the equality (4.1) becomes

$$\begin{aligned} {\mathcal {F}}(\lambda G; \lambda E_0,\tau ) = \lambda ^{n-1} P_\varphi (G) + \lambda ^{n-1} \int _G f\left( \tfrac{\textrm{sd}_{E_0}}{\tau \lambda ^{-1/\alpha }}\right) ~dx = {\mathcal {F}}(G; E_0,\tau \lambda ^{-1/\alpha }). \end{aligned}$$

Thus $\lambda G$ is a minimizer of ${\mathcal {F}}(\cdot ;\lambda E_0,\tau )$ if and only if *G* is a minimizer of ${\mathcal {F}}(\cdot ;E_0,\tau \lambda ^{-1/\alpha }).$

Now, if the sequence $\tau _j\rightarrow 0$ and flat flows $\{\lambda E(\tau _j,k)\},$ starting from $\lambda E_0,$ associated to $\mathcal {F}_{\varphi ,f,0},$ are such that

4.2 $$\begin{aligned} \lim \limits _{j\rightarrow +\infty } \lambda E(\tau _j,\left\lfloor t/\tau _j \right\rfloor ) = \lambda E(t)\quad \hbox {for all}\,\, t \ge 0 \,\, \hbox {in} \,\, L_\textrm{loc}^1(\mathbb {R}^n) \end{aligned}$$

then $E(\tau _j,k)$ are flat flows starting from $E_0,$ associated to $\mathcal {F}_{\varphi ,f,0},$ but with the time step equal to $\tau \lambda ^{-1/\alpha }.$ Thus, by (4.2),

$$\begin{aligned} \lim \limits _{j\rightarrow +\infty }  &   E(\tau _j,\left\lfloor t/(\lambda ^{-1/\alpha }\tau _j) \right\rfloor ) = \lim \limits _{j\rightarrow +\infty } E(\tau _j,\left\lfloor t \lambda ^{1/\alpha }/\tau _j \right\rfloor ) = E(t\lambda ^{1/\alpha })\\  &   \quad \text {for all }t\ge 0\hbox { in } L_\textrm{loc}^1({\mathbb {R}}^n). \end{aligned}$$

The converse assertion is done in a similar manner. See also [8]. $\square$

### Theorem 4.3

(Comparison with balls) Suppose Hypothesis (H) and $g\in L^\infty ({\mathbb {R}}^n).$ Given $E_0\in \mathcal {S}^*$ and $\tau >0,$ let $\{E(\tau ,k)\}$ be flat flows starting from $E_0.$ Let $r_0>0$. Then there exist ${\widehat{\tau }}_1>0$ and $C_8>0$ depending only on *n*,  $\varphi ,$ $f$, $r_0$ and $\Vert g\Vert _\infty$, such that

4.3 $$\begin{aligned} W_{r_0}(x_0)\subset E_0\quad \Longrightarrow \quad W_{r_0-C_8k\tau }(x_0) \subset E(\tau ,k) \end{aligned}$$

and

4.4 $$\begin{aligned} W_{r_0}(x_0)\cap E_0= \emptyset \quad \Longrightarrow \quad W_{r_0-C_8k\tau } (x_0) \cap E(\tau ,k) =\emptyset \end{aligned}$$

for all $\tau \in (0,{\widehat{\tau }}_1)$ and $0\le k\tau \le \frac{r_0}{2C_8}.$

### Proof

Let $\tau _0$ be given by (3.9), where we recall that $\gamma _g$ is given in (A.3). We prove only (4.3), the relation (4.4) being similar. For shortness, we assume $x_0=0$ and let $W_{r_0}\subset E_0.$

*Step 1.* Since $\rho _\tau \rightarrow 0$ as $\tau \rightarrow 0^+,$ there exists $\tau _1\in (0,\tau _0)$ (depending only on $r_0$) such that $\rho _{\tau _1}<r_0/10.$ For $\tau \in (0,\tau _1)$ let $E_\tau$ be a minimizer of ${\mathcal {F}}(\cdot ;E_0,\tau ).$ By the choice of $\tau _1$ and the $L^\infty$-bound (3.10), we have $W_{4r_0/5}\subset E_\tau .$ Let

$$\begin{aligned} {\bar{r}}:=\sup \{r>0:\,\, W_r\subset E_\tau \}\ge 4r_0/5. \end{aligned}$$

We want to estimate ${\bar{r}}$ from below (see (4.9)), thus there is no loss of generality in assuming ${\bar{r}}<r_0.$ Fix $\epsilon \in (0,r_0-{\bar{r}})$ and consider the difference

4.5 $$\begin{aligned}  &   0\le {\mathcal {F}}(E_\tau \cup W_{\bar{r}+\epsilon }; E_0,\tau ) - {\mathcal {F}}(E_\tau ; E_0,\tau ) \nonumber \\  &   \quad = P_\varphi (E_\tau \cup W_{{\bar{r}}+\epsilon }) - P_\varphi (E_\tau ) + \int _{W_{\bar{r}+\epsilon }\setminus E_\tau } f\left( \tfrac{\textrm{sd}_{E_0}}{\tau }\right) ~dx + \int _{W_{{\bar{r}}+\epsilon }\setminus E_\tau } g~dx.\nonumber \\ \end{aligned}$$

Using $W_{{\bar{r}}+\epsilon } \subset W_{r_0}\subset E_0$ we find $-\textrm{sd}_{E_0} = \textrm{d}_{E_0} \ge \frac{1}{C_{\varphi }}\textrm{d}_{ E_0}^\varphi \ge \frac{r_0-{\bar{r}} - \epsilon }{C_{\varphi }}$ in $W_{{\bar{r}}+\epsilon }{\setminus } E_\tau ,$ where $d^\varphi _F$ stands for the $\varphi$-distance from the set *F*. Therefore, by the strict monotonicity and oddness of $f,$

4.6 $$\begin{aligned} -\int _{W_{{\bar{r}}+\epsilon }\setminus E_\tau } f\left( \tfrac{\textrm{sd}_{E_0}}{\tau }\right) ~dx \ge f\Big (\tfrac{r_0-\bar{r}-\epsilon }{C_{\varphi }\tau }\Big )| W_{{\bar{r}}+\epsilon }\setminus E_\tau |. \end{aligned}$$

Moreover, by the boundedness of $g,$

4.7 $$\begin{aligned} \int _{W_{{\bar{r}}+\epsilon }\setminus E_\tau } g~dx \le \Vert g\Vert _\infty |W_{{\bar{r}}+\epsilon }\setminus E_\tau |. \end{aligned}$$

Finally, using the anisotropic isoperimetric inequality (2.2) for a.e. $\epsilon >0$ we have

4.8 $$\begin{aligned} \begin{aligned} P_\varphi (E_\tau \cup W_{{\bar{r}}+\epsilon }) - P_\varphi (E_\tau ) =&P_\varphi (W_{\bar{r}+\epsilon }) - P_\varphi (E_\tau \cap W_{{\bar{r}}+\epsilon }) \\ \le&c_{n, \varphi }\Big (|W_{{\bar{r}}+\epsilon }|^{\frac{n-1}{n}} - |W_{{\bar{r}}+\epsilon }\cap E_\tau |^{\frac{n-1}{n}} \Big ) \\ =&c_{n, \varphi }|W_{{\bar{r}}+\epsilon }|^{\frac{n-1}{n}}\Big (1 - \Big |1 - \frac{|W_{{\bar{r}}+\epsilon }\setminus E_\tau |}{|W_{{\bar{r}}+\epsilon }|}\Big |^{\frac{n-1}{n}}\Big ) \\ \le&\frac{c_{n, \varphi }}{|{B_\varphi }|^{1/n}({\bar{r}}+\epsilon )} |W_{{\bar{r}}+\epsilon } \setminus E_\tau |, \end{aligned} \end{aligned}$$

where in the last inequality we used $(1-x)^\alpha \ge 1-x$ for any $x,\alpha \in (0,1)$. Inserting (4.6), (4.7), (4.8) in (4.5) and using the arbitrariness of $\epsilon$ we get

$$\begin{aligned} f\Big (\frac{r_0-{\bar{r}}}{C_{\varphi }\tau }\Big ) \le \frac{c_{n, \varphi }}{ |{B_\varphi }|^{1/n}\,{\bar{r}}} + \Vert g\Vert _\infty . \end{aligned}$$

Thus, recalling ${\bar{r}}\ge 4r_0/5$ we get $\frac{r_0-{\bar{r}}}{ C_{\varphi }\tau } \le f^{-1}\Big (\frac{5 c_{n, \varphi }}{4|{B_\varphi }|^{1/n}\,r_0} + \Vert g\Vert _\infty \Big )$, or equivalently

4.9 $$\begin{aligned} {\bar{r}} \ge r_0 - C_{\varphi }\tau f^{-1}\Big (\frac{5 c_{n, \varphi }}{4|{B_\varphi }|^{1/n}\,r_0} + \Vert g\Vert _\infty \Big ). \end{aligned}$$

Notice that this inequality holds also in case ${\bar{r}}\ge r_0.$

*Step 2.* Let $\{E(\tau ,k)\}$ be a flat flow starting from $E_0$ and let $\{F(\tau ,k)_*\}$ be a flat flow starting from $F_0:= W_{r_0}$ and consisting of the minimal minimizers (see Corollary B.3 for definition). By comparison (Theorem B.2 (c)), $F(\tau ,k)_* \subset E(\tau ,k)$ for all $k\ge 0.$

Consider the sequence $r_0=r(\tau ,0)\ge r(\tau ,1)\ge \ldots$ of radii defined as follows: we assume $W_{r(\tau ,k)}\subset F(\tau ,k)_*$ and if $r(\tau ,k-1)>r(\tau ,k),$ then $W_{r(\tau ,k)}$ is the largest Wulff shape contained in $F(\tau ,k)_*.$ Let $k_0\ge 1$ be such that $r(\tau ,k_0)\ge r_0/2$ and let $\tau _1>\tau _2>\ldots>\tau _{k_0}>0$ be given by step 1 applied with $r_0:=r(\tau ,k)$ for $k=1,\ldots ,k_0.$ Thus, for any $\tau \in (0,\tau _k)$ one has $r(\tau ,k)>4r(\tau ,k-1)/5.$ From step 1 it follows

$$\begin{aligned} r(\tau ,k) \ge r(\tau ,k-1) - C_{\varphi }\tau f^{-1}\Big ( \frac{5c_{n, \varphi }}{4|{B_\varphi }|^{1/n} r(\tau ,k-1)} + \Vert g\Vert _\infty \Big ),\quad 1\le k\le k_0. \end{aligned}$$

Now, by the choice of $k_0,$

$$\begin{aligned} r(\tau ,k) \ge r(\tau ,k-1) - C_{\varphi }\tau f^{-1}\Big ( \frac{5 c_{n, \varphi }}{2|{B_\varphi }|^{1/n} r_0} + \Vert g\Vert _\infty \Big ),\quad 1\le k\le k_0, \end{aligned}$$

and hence

$$\begin{aligned} r(\tau ,k) \ge r_0 - C_{\varphi }f^{-1}\Big ( \frac{5 c_{n, \varphi }}{2|{B_\varphi }|^{1/n} r_0} + \Vert g\Vert _\infty \Big )k\tau ,\quad 0\le k\le k_0. \end{aligned}$$

Now, if we assume

$$\begin{aligned} C_8:=C_8(n,\varphi , f, r_0, \Vert g\Vert _\infty ):= C_{\varphi }f^{-1}\Big ( \frac{5c_{n, \varphi }}{2|{B_\varphi }|^{1/n} r_0} + \Vert g\Vert _\infty \Big ) \end{aligned}$$

and $k\tau \le \frac{r_0}{2C_8},$ we get $r(\tau ,k)\ge r_0/2$ and $r(\tau ,k)\ge r_0-C_8k\tau .$ $\square$

It turns out that $\textrm{GMM}$s for generalized power mean curvature flow share several properties with $\textrm{GMM}$s in the anisotropic mean curvature flow, as we now see. Let us study $\textrm{GMM}$ starting from a Euclidean ball centered at origin.

### Theorem 4.4

(Evolution of balls) Assume that $\varphi$ is the Euclidean norm and $g\equiv 0$. Suppose also that $r\mapsto f^{-1}((n-1)/r)$ is such that the ordinary differential equation

4.10 $$\begin{aligned} {\left\{ \begin{array}{ll} r'(t) = - f^{-1}\Big (\frac{n-1}{r(t)}\Big ) &  \hbox { if}\ r(t)>0,\\ r(0) = r_0 >0 \end{array}\right. } \end{aligned}$$

admits a unique $C^1$ solution (for instance, in the case $r\mapsto f^{-1}((n-1)/r)$ is locally Lipschitz in $(0,+\infty )$). Then $\textrm{GMM}(\mathcal {F},\mathcal {S}^*,B_{r_0})$ is a singleton $\{B_{r(t)}\}_{t\ge 0},$ where $r(\cdot )$ is a nonincreasing function satisfying (4.10).

### Proof

We can write

$$\begin{aligned} {\mathcal {F}}(E;E_0,\tau ):=P(E) + \int _E f\left( \tfrac{\textrm{sd}_{E_0}}{\tau }\right) ~dx + c_{E_0}. \end{aligned}$$

*Step 1: properties of minimizers.* Fix $r_0>0$. Let $\tau _0$ and $\rho _\tau$ be as in (3.9), (3.10), so that

$$\begin{aligned} \sup _{x \in \overline{E_\tau \Delta B_{r_0}}}\,\textrm{d}_{B_{r_0}}(x)\,\le 2\rho _\tau ,\quad \tau \in (0,\tau _0), \end{aligned}$$

for any minimizer $E_\tau$ of ${\mathcal {F}}(\cdot ;B_{r_0},\tau ).$ Since $\rho _\tau \rightarrow 0^+$ as $\tau \rightarrow 0^+,$ there exists $\tau _{r_0}\in (0,\tau _0)$ such that $B_{r_0/2}\subset E_\tau \subset B_{3r_0/2}$ for all $\tau \in (0,\tau _{r_0})$.

Let us study the minimal and maximal minimizers. Owing to

$$\begin{aligned} f\Big (\frac{\textrm{sd}_{B_{r_0}}(x)}{\tau } \Big ) = f\Big (\frac{|x|-r_0}{\tau }\Big ),\quad x\in {\mathbb {R}}^n, \end{aligned}$$

the volume term of $\mathcal {F}$ is radially symmetric. Therefore, the minimal and maximal minimizers of ${\mathcal {F}}(\cdot ;B_{r_0},\tau )$ are radially symmetric, i.e., both of them are balls. By the choice of $\tau _{r_0},$ their radii are in the interval $(r_0/2,3r_0/2).$ In particular, the radii of minimal and maximal minimizers satisfy (1.6). Without loss of generality, we may assume $r_0\mapsto \tau _{r_0}$ is increasing.

*Step 2: some properties of radially symetric flat flows.* Given $r_0>0$ and $\tau >0,$ let the numbers $r(\tau ,k)\ge 0$ be defined as follows: $r(\tau ,0) = 0$ and for $k\ge 1,$ the ball $B_{r(\tau ,k)}$ is a minimizer of ${\mathcal {F}}(\cdot ; B_{r(\tau ,k-1)},\tau )$ (we can construct such a sequence as, by step 1, the minimal and maximal minimizers starting from a ball are in fact balls). We note that $k\mapsto r(\tau ,k)$ decreases and there exists (the smallest) $k_\tau >0$ such that $r(\tau ,k)=0$ for all $k>k_\tau .$ Indeed, if $r(\tau ,k)>0$ for some $k>0,$ then in view of (1.6)

4.11 $$\begin{aligned} \frac{r(\tau ,k) - r(\tau ,k-1)}{\tau } = -f^{-1}\Big (\frac{n-1}{r(\tau ,k)}\Big ). \end{aligned}$$

Hence, $r(\tau ,k)<r(\tau ,k-1).$ In case $r(\tau ,k)>0$ for all $k\ge 1,$ then summing (1.6) in $k=1,\ldots ,N$ and using $r(\tau ,k)< r_0$ and the strict monotonicity of $f^{-1}$ we find

$$\begin{aligned} \frac{r_0-r(\tau ,N)}{\tau } \ge N f^{-1}\Big (\tfrac{n-1}{r_0}\Big ). \end{aligned}$$

Since $r_0<+\infty ,$ for large *N* this inequality is not possible.

Let $\epsilon \in (0,1/4),$ Since $k\in \{0,\ldots ,k_\tau \}\mapsto r(\tau ,k)$ strictly decreases and $r(\tau ,k)=0$ for $k>k_\tau$, there exists a unique ${\bar{k}}_{\tau ,\epsilon }>0$ such that $r(\tau ,{\bar{k}}_{\tau ,\epsilon }+1)\le \epsilon r_0 < r(\tau ,\bar{k}_{\tau ,\epsilon }).$ One can readily check that both maps $\tau \mapsto k_{\tau ,\epsilon }$ and $\epsilon \mapsto k_{\tau ,\epsilon }$ are decreasing. Let

$$\begin{aligned} T_\epsilon :=\liminf \limits _{\tau \rightarrow 0^+} ~(\tau k_{\tau ,\epsilon }). \end{aligned}$$

By the monotonicity of $k_{\tau ,\epsilon },$ $\epsilon \mapsto T_\epsilon$ is nondecreasing. Let us show that it is uniformly away from 0. Indeed, since $B_{r(\tau ,k)},$ $0\le k\le k_{\tau ,\epsilon }$ are flat flows, applying (3.22) with $m_1=1$ and $m_2=k_{\tau ,\epsilon }+1$ we get

$$\begin{aligned} |B_{r(\tau ,1)}\setminus B_{r(\tau ,k_{\epsilon ,\tau }+1)}| \le C_4p^\sigma (\tau k_{\tau ,\epsilon } + \tau - \tau ) + \frac{1}{f(p)} \end{aligned}$$

for some $p>0$ and suitable $\sigma \in \{1,n\}.$ Now, letting $\tau \rightarrow 0^+$ and recalling $B_{r(\tau ,1)}\rightarrow B_{r_0},$ we get

$$\begin{aligned} |B_{r_0}\setminus B_{\epsilon r_0}| \le C_4p^\sigma T_\epsilon + \frac{1}{f(p)}. \end{aligned}$$

Now, taking *p* large enough (depending only on $r_0$) we deduce $T_{\epsilon }> C_{11}$ for some $C_{11}$ depending only on $C_4$ and $r_0.$ Let us denote

$$\begin{aligned} T_0:=\sup _{\epsilon \in (0,1/4)} T_\epsilon . \end{aligned}$$

By the definition, $T_\epsilon \le T_0.$

*Step 3:* Now assume that $\tau _j\rightarrow 0^+$ is such that

$$\begin{aligned} B_{r(\tau _j,\left\lfloor t/\tau _j \right\rfloor )} \rightarrow B_{r(t)}\quad \text {in } L^1({\mathbb {R}}^n)\hbox { as }j\rightarrow +\infty \quad \forall t\ge 0, \end{aligned}$$

or equivalently,

4.12 $$\begin{aligned} \lim \limits _{j\rightarrow +\infty } r(\tau _j,\left\lfloor t/\tau _j \right\rfloor ) = r(t),\quad t\ge 0. \end{aligned}$$

Fix any ${\bar{T}}<T_0$ and let ${\bar{\epsilon }}>0$ be such that $T_{\bar{\epsilon }}\in ({\bar{T}},T_0).$ Then for any $\tau _j<t<{\bar{T}}$ with $1\le k:=\left\lfloor t/\tau _j \right\rfloor < \left\lfloor T_{{\bar{\epsilon }}}/\tau _j \right\rfloor$ and $r(\tau _j,\left\lfloor t/\tau _j \right\rfloor )\ge {\bar{\epsilon }} r_0>0,$ applying (4.11) we find

$$\begin{aligned} \frac{r(\tau _j,\left\lfloor t/\tau _j \right\rfloor ) - r(\tau _j,\left\lfloor (t-\tau _j)/\tau _j \right\rfloor ) }{\tau _j} = -f^{-1}\Big (\frac{n-1}{r(\tau _j,\left\lfloor t/\tau _j \right\rfloor )}\Big ). \end{aligned}$$

Passing to the limit in this difference equation and using (4.12) we obtain

4.13 $$\begin{aligned} r'(t) = - f^{-1}\Big (\frac{n-1}{r(t)}\Big ),\quad t\in (0,\bar{T}). \end{aligned}$$

By the definition of $T_\epsilon$ we can show that $\lim _{\bar{T}\nearrow T_0} r({\bar{T}})=0.$ Since ${\bar{T}}<T_0$ is arbitrary, the radii *r*(*t*) of balls necessarily satisfy (4.13).

*Step 4: properties of* $\textrm{GMM}$. Consider any $E(\cdot )\in GMM({\mathcal {F}},\mathcal {S}^*,B_{r_0})$ and the flat flows $\{E(\tau _j,k)\}$ satisfying

$$\begin{aligned} \lim \limits _{j\rightarrow +\infty }|E(\tau _j,\left\lfloor t/\tau _j \right\rfloor ) \Delta E(t)|=0\quad \hbox { for all}\ t\ge 0. \end{aligned}$$

Consider also the flat flows $\{E(\tau _j,k)^*\}$ and $\{E(\tau _j,k)_*\}$ consisting of maximal and minimal minimizers of ${\mathcal {F}}$ and starting from $B_{r_0}.$ In view of step 1, they are family of balls with radii $\{r^*(\tau _j,k)\}$ and $\{r_*(\tau _j,k)\},$ respectively. Moreover, by comparison principles (see, for instance, Theorem B.2 and also the proof of Theorem B.5)

$$\begin{aligned} B_{r_*(\tau _j,k)} \subset E(\tau _j,k)\subset B_{r^*(\tau _j,k)}. \end{aligned}$$

Passing to a not relabelled subsequence $\{\tau _j\}$ if necessary, we assume that

$$\begin{aligned} B_{r_*(\tau _j,\left\lfloor t/\tau _j \right\rfloor )} \rightarrow B_{r_*(t)}\quad \text {in } L^1(\mathbb {R}^n)\hbox { for all }t\ge 0 \end{aligned}$$

and

$$\begin{aligned} B_{r^*(\tau _j,\left\lfloor t/\tau _j \right\rfloor )} \rightarrow B_{r^*(t)}\quad \text {in } L^1(\mathbb {R}^n)\hbox { for all }t\ge 0, \end{aligned}$$

so that $\{B_{r_*(t)}\},\{B_{r^*(t)}\} \in GMM({\mathcal {F}},\mathcal {S}^*,B_{r_0})$ and

4.14 $$\begin{aligned} B_{r_*(t)} \subset E(t)\subset B_{r^*(t)},\quad t\ge 0. \end{aligned}$$

In view of step 3, both $r_*(\cdot )$ and $r^*(\cdot )$ solve (4.13). However, by assumption of the theorem this ODE admits a unique positive solution $r(\cdot )$ in $(0,T_0)$ and then extended to 0 for $t\ge T_0$. Hence, $r_*(t)=r^*(t)=r(t)$ for all *t* and thus, by (4.14) $E(t)=B_{r(t)}.$ This implies $GMM({\mathcal {F}},\mathcal {S}^*,B_{r_0})$ is a singleton. $\square$

### Remark 4.5

Let $f(r)=r^\alpha$ in $(0,+\infty )$ for $\alpha >0.$ Then the ODE in (4.10) reads as

$$\begin{aligned} r' = -\frac{(n-1)^{1/\alpha }}{r^{1/\alpha }}. \end{aligned}$$

Thus, $$\textrm{GMM}(\mathcal {F},\mathcal {S}^*,B_{r_0}) = \{B_{r(t)}\},$$ where

$$\begin{aligned} r(t) = {\left\{ \begin{array}{ll} \Big (r_0^{1+\frac{1}{\alpha }} - \big (1+\tfrac{1}{\alpha }\big )(n-1)^{\frac{1}{\alpha }}t\Big )^{\frac{\alpha }{1+\alpha }} &  \text {if }t\in \Big [0,\tfrac{r_0^{1+1/\alpha }}{\big (1+1/\alpha \big )(n-1)^{1/\alpha } }\Big ),\\ 0 &  \text {otherwise.} \end{array}\right. } \end{aligned}$$

## Evolution of Mean Convex Sets: Proof of Theorem 1.5

In this section we mostly follow the ideas of [13]. Apart from some technical points due to the presence of the function $f,$ one difference here is in the proof of the $\delta$-convexity preservation of minimizers (Proposition 5.3): we apply directly the prescribed mean curvature functional in place of 1-harmonic functions and Anzellotti-type arguments as done in [13]. We assume here that $g\equiv 0,$ $\tau >0$ and $k\ge 0.$

Given an anisotropy $\varphi$ in ${\mathbb {R}}^n,$ $\delta \ge 0$ and a (nonempty) open set $\Omega \subset {\mathbb {R}}^n,$ a set $E\Subset \Omega$ is called $\delta$-*mean convex* (in $\Omega$) if

5.1 $$\begin{aligned} P_\varphi (E)-\delta |E| \le P_\varphi (F)-\delta |F|\quad \text {for any } F\Subset \Omega \hbox { with }E\subset F. \end{aligned}$$

When $\delta =0,$ we simply say *E* is mean convex. Using (5.1) with $F=E\cup B_r(x)$ for some ball $B_r(x)\Subset \Omega$, we can readily show the existence of $\vartheta >0$ depending only on *n*,  $c_{\varphi }$, $C_{\varphi }$ such that every $\delta$-mean convex set *E* satisfies

$$\begin{aligned} \frac{|B_r(x)\setminus E|}{|B_r(x)|} \ge \vartheta >0 \end{aligned}$$

for all $x\in \Omega$ and for all $0<r<\textrm{dist}(x,\partial \Omega ).$ In particular, $E=E^{(1)}$ is open. Let us recall some further properties of $\delta$-mean convex sets.

### Proposition 5.1

([13, 18]) Let $$\delta \ge 0.$$

- Suppose there exists a $\delta$-mean convex set $E\Subset \Omega$ in $\Omega .$ Then $\emptyset$ is $\delta$-mean convex in $\Omega$.
- $E\Subset \Omega$ is $\delta$-mean convex in $\Omega$ if and only if
  $$\begin{aligned} P_\varphi (E) \le P_\varphi (E\cup F) - \delta |F\setminus E|,\quad F\Subset \Omega . \end{aligned}$$
- $E\Subset \Omega$ is $\delta$-mean convex in $\Omega$ if and only if
  $$\begin{aligned} P_\varphi (E\cap F) \le P_\varphi (F) - \delta |F\setminus E|,\quad F\Subset \Omega . \end{aligned}$$
- Let $E_h\Subset \Omega ,$ $h=1,2,\ldots ,$ be $\delta$-mean convex in $\Omega$ with $E_h\rightarrow E$ in $L^1(\Omega )$ for some $E\Subset \Omega .$ Then *E* is $\delta$-mean convex.

Since we are mostly interested in bounded sets, in view of (1.12) we can write, for a constant $c _F$ independent of *E*,

$$\begin{aligned} {\mathcal {F}}(E;F,\tau ):= P_\varphi (E)+\int _Ef\left( \tfrac{\textrm{sd}_{F}}{\tau }\right) ~dx +c _F\end{aligned}$$

if *F* is bounded, see (1.13).

### Lemma 5.2

(Inclusion of minimizers) Let $F\Subset \Omega$ be an open $\delta$-mean convex set in $\Omega$ for some $\delta \ge 0.$ Suppose that $f$ satisfies (1a), (1b), and that $g\equiv 0$. Let *E* be a minimizer of ${\mathcal {F}}(\cdot ;F,\tau ).$ Then:

(i) $$\overline{E} \subset \overline{F};$$ if $$\delta >0,$$ then $$E\subset F,$$
(ii) if $$\delta >0,$$
  5.2 $$\begin{aligned} \bigcup _{|\eta |<f^{-1}(\delta )\tau } (E+\eta ) \subset \overline{F}\quad \text {and}\quad \textrm{sd}_E \ge \textrm{sd}_F + f^{-1}(\delta )\tau \,\,\,\hbox { in}\ {\mathbb {R}}^n\end{aligned}$$
  provided $$\textrm{dist}(F+\delta \tau ,\partial \Omega )>0,$$
(iii) *E* is mean convex.

### Proof

(i) By the $\delta$-mean convexity of *F* in $\Omega$

5.3 $$\begin{aligned} P_\varphi (F)\le P_\varphi (F\cup E) -\delta |E\setminus F|, \end{aligned}$$

and by the minimality of *E*

$$\begin{aligned} P_\varphi (E) +\int _Ef\left( \tfrac{\textrm{sd}_{F}}{\tau }\right) ~dx \le P_\varphi (E\cap F) +\int _{E\cap F}f\left( \tfrac{\textrm{sd}_{F}}{\tau }\right) ~dx. \end{aligned}$$

Summing these inequalities we get

5.4 $$\begin{aligned} \int _{E\setminus F} f\left( \tfrac{\textrm{sd}_{F}}{\tau }\right) ~dx + \delta |E\setminus F| \le P_\varphi (E\cup F)+ P_\varphi (E\cap F)- P_\varphi (E)-P_\varphi (F)\le 0, \end{aligned}$$

where in the last inequality we used the submodularity of $P_\varphi .$ Since $\textrm{sd}_F>0$ in ${\mathbb {R}}^n{\setminus } \overline{F}$ and $f>0$ in $\mathbb {R}^+,$ from (5.4) we deduce $|E{\setminus } \overline{F}|=0,$ i.e., $E\subset \overline{F},$ hence, $\overline{E}\subset \overline{F}.$ Note that if $\delta >0,$ then $E\subset F.$

(ii) Assume $\delta >0$ and $\eta \in B_{f^{-1}(\delta )\tau }.$ Clearly, $F+\eta$ is $\delta$-mean convex in $\Omega +\eta$ and $E+\eta$ is a minimizer of ${\mathcal {F}}(\cdot ;F+\eta ,\tau ).$ By the choice of $\tau ,$ $E+\eta \Subset \Omega$ and hence we can apply (5.3) with $E:=E+\eta .$ Moreover,

$$\begin{aligned} P_\varphi (E+\eta ) +\int _{E+\eta }f\left( \tfrac{\textrm{sd}_{F+\eta }}{\tau }\right) ~dx \le P_\varphi ([E+\eta ]\cap F) +\int _{[E+\eta ]\cap F}f\left( \tfrac{\textrm{sd}_{F+\eta }}{\tau }\right) ~dx. \end{aligned}$$

Summing this and (5.3) (applied with $E=E+\eta$) we deduce

$$\begin{aligned}  &   \int _{[E+\eta ]\setminus F}\Big (f\left( \tfrac{\textrm{sd}_{F+\eta }}{\tau }\right) + \delta \Big )~dx \le P_\varphi ([E+\eta ]\cup F)+ P_\varphi ([E+\eta ]\cap F) \\  &   \quad - P_\varphi (F) - P_\varphi (E+\eta )\le 0. \end{aligned}$$

Thus,

5.5 $$\begin{aligned} \int _{[E+\eta ]\setminus F}\Big (f\left( \tfrac{\textrm{sd}_{F+\eta }}{\tau }\right) + \delta \Big )~dx = \int _{[E+\eta ]\setminus F}\Big (f\left( \tfrac{\textrm{sd}_{F}}{\tau }\right) + \delta \Big )~dx \le 0. \end{aligned}$$

Note that if $x\in [E+\tau ]\setminus F$ then, by (a), $x-\eta \in E\subset F,$ and hence

$$\begin{aligned} \textrm{sd}_F(x - \eta ) = -\textrm{d}_F(x-\eta ) \ge - |x - (x-\eta )|= -|\eta |>-f^{-1}(\delta )\tau . \end{aligned}$$

Then by the strict monotonicity of $f,$

$$\begin{aligned} f\left( \tfrac{\textrm{sd}_{F}}{\tau }\right) + \delta >0\quad \text {on }[E+\tau ]\setminus F \end{aligned}$$

and therefore, by (5.5), $E+\eta \subset \overline{F}.$

Let us prove $\textrm{sd}_E\ge \textrm{sd}_F + f^{-1}(\delta )\tau .$ We know that $E\subset F$ and

5.6 $$\begin{aligned} \inf _{x\in \partial F,\,y\in \partial E}\,\,\, |x-y| \ge f^{-1}(\delta )\tau . \end{aligned}$$

Take $x\in F^c$ and let $y\in \partial E$ be such that $\textrm{d}_E(x) = |x-y|.$ Then there exists $z\in [x,y]\cap \partial F,$ and by the definition of $\textrm{d}_F$ and (5.6),

$$\begin{aligned} \textrm{sd}_E(x)= &   \textrm{d}_E(x) = |x-y| = |x-z| + |z-y| \ge \textrm{d}_F(x) +f^{-1}(\delta )\tau \\= &   \textrm{sd}_F(x) +f^{-1}(\delta )\tau . \end{aligned}$$

Now, assume that $x\in F\setminus E$ and let $y\in \partial F$ and $z\in \partial E$ be such that $\textrm{d}_F(x)=|x-y|$ and $\textrm{d}_E(x)=|x-z|.$ Then by (5.6),

$$\begin{aligned} \textrm{d}_E(x)+\textrm{d}_F(x) = |x-y|+|x-z|\ge |y-z| \ge f^{-1}(\delta )\tau , \end{aligned}$$

and therefore

$$\begin{aligned} \textrm{sd}_E(x) = \textrm{d}_E(x)\ge -\textrm{d}_F(x) + f^{-1}(\delta )\tau = \textrm{sd}_F(x)+f^{-1}(\delta )\tau . \end{aligned}$$

Finally, assume that $x\in E$ and let $y\in \partial F$ be such that $\textrm{d}_F(x)=|y-x|.$ Then there exists $z\in [x,y]\cap \partial E$ and hence, as above,

$$\begin{aligned} -\textrm{sd}_F(x)=\textrm{d}_F(x) = |x-z| + |z-y| \ge \textrm{d}_E(x) +f^{-1}(\delta )\tau = -\textrm{sd}_E(x) +f^{-1}(\delta )\tau . \end{aligned}$$

(iii) We claim that $P_\varphi (E)\le P_\varphi (G)$ for any $G\Subset \Omega$ with $E\subset G.$ Indeed, by the $\delta$-mean convexity of *F* and Proposition 5.1 (c), it follows

5.7 $$\begin{aligned} P_\varphi (F\cap G) \le P_\varphi (G) - \delta |G\setminus F|. \end{aligned}$$

Moreover, by the minimality of *E*, 

$$\begin{aligned} P_\varphi (E) + \int _{E}f\left( \tfrac{\textrm{sd}_{F}}{\tau }\right) ~dx \le P_\varphi (F\cap G) + \int _{F\cap G}f\left( \tfrac{\textrm{sd}_{F}}{\tau }\right) ~dx. \end{aligned}$$

Since $E\subset F\cap G$ and $f$ is odd, this inequality becomes

$$\begin{aligned} P_\varphi (E) + \int _{[F\cap G]\setminus E} f\Big (-\tfrac{\textrm{sd}_F }{\tau }\Big )~dx \le P_\varphi (F\cap G). \end{aligned}$$

The integral in this inequality is nonnegative. Therefore, by (5.7),

$$\begin{aligned} P_\varphi (E) \le P_\varphi (F\cap G) \le P_\varphi (G) - \delta |G\setminus F|\le P_\varphi (G). \end{aligned}$$

$$\square $$

The following proposition improves the last assertion of Lemma 5.2.

### Proposition 5.3

(Mean convexity of minimal and maximal minimizers) Suppose that $f$ satisfies (1a), (1b), and that $g\equiv 0$. Let $E_0\Subset \Omega$ be an open $\delta$-mean convex set in $\Omega$ for some $\delta >0.$ Then the minimal and maximal minimizers of ${\mathcal {F}}(\cdot ;E_0,\tau )$ (in the sense of Corollary B.3) are $\delta$-mean convex in $\Omega$.

### Proof

For any $s>0$ let $E_s$ be a minimizer of ${\mathcal {F}}(\cdot ;E_0,s).$

*Step 1:* For any $0< s'< s''$

$$\begin{aligned} E_{s''}\subset E_{s'}\subset E_0. \end{aligned}$$

The inclusion $E_{s''},E_{s'}\subset \overline{E_0}$ follows from Lemma 5.2 (i). To prove the first inclusion it is enough to observe that $s\mapsto \frac{\textrm{sd}_{E_0}}{s}$ is strictly decreasing in $E_0.$ Now, the inclusion follows from the comparison principle in Corollary B.4 for the prescribed mean curvature functional.

*Step 2:*

$$\begin{aligned} \lim \limits _{s\searrow 0}|E_0\setminus E_s|=0. \end{aligned}$$

Note that this assertion was already shown in (3.28) under the extra assumption $|\partial E_0|=0$. Here we do not have such a regularity. By minimality, we have

$$\begin{aligned} P_\varphi (E_{s}) + \int _{E_{s}}f\left( \tfrac{\textrm{sd}_{E_0}}{s}\right) ~dx \le P_\varphi (E_0) + \int _{E_0}f\left( \tfrac{\textrm{sd}_{E_0}}{s}\right) ~dx, \end{aligned}$$

and using $$E_{s}\subset E_0$$,

$$\begin{aligned} P_\varphi (E_{s}) + \int _{E_0\setminus E_{s}}f\left( \tfrac{\textrm{d}_{E_0}}{s}\right) ~dx \le P_\varphi (E_0). \end{aligned}$$

This, the monotonicity of $s\mapsto E_{s}$ and the openness of $E_{s}$ and $E_0$ imply $E_{s}\overset{L^1}{\rightarrow }\ E_0$ and $P_\varphi (E_{s})\rightarrow P_\varphi (E_0)$ as $s\searrow 0.$

*Step 3.*

$$\begin{aligned} \lim \limits _{s\nearrow \tau }\,|E_s\setminus E_\tau ^*|=0 \quad \text {and} \quad \lim \limits _{s\searrow \tau }\,|E_{\tau *}\setminus E_s|=0. \end{aligned}$$

We start by proving the first equality. Consider any sequence $s_i\nearrow \tau .$ Since $P_\phi (E_{s_i})\le P_\phi (E_0)$ and $E_{s_i}\subset E_0$ for any $i\ge 1,$ there exists $Q\subset E_0$ such that, up to a further not relabelled subsequence, $E_{s_i}\searrow Q$ in $L^1(\mathbb {R}^n).$ By the $L^1$-lower semicontinuity of ${\mathcal {F}}(\cdot ;E_0,\tau ),$ *Q* is its minimizer. Moreover, since $E_{s_i}\supset E_{s_{i+1}},$ we have also $E_{s_i}\supset Q$ for any $i\ge 1.$ By step 1, $E_{s_i}\supset E_{\tau }^*,$ and hence, we cannot have $|E_{\tau }^*\setminus Q|>0.$ Now, arbitrariness of $s_i$ implies $E_s\searrow E_{\tau }^*$ as $s\nearrow \tau .$

The proof of the second equality is similar.

*Step 4.* Now, we prove the $\delta$-mean convexity of the minimal and maximal minimizers.

Fix any $G\in \mathcal {S}^*$ with $G\Subset \Omega ,$ $\epsilon \in (0,\delta )$ and $0<\underline{s}<\overline{s}.$ For any $N>1$ let

$$\begin{aligned} s_i:= \underline{s} + \frac{(\overline{s} - \underline{s})i}{N}, \quad i=0,\ldots ,N. \end{aligned}$$

Possibly slightly perturbing *G* we may assume that

$$\begin{aligned} \sum _{i=0}^N \mathcal {H}^{n-1}(\partial ^*G \cap \partial ^*E_{s_i}) = 0; \end{aligned}$$

define also $G_i:=G\cap [E_{s_{i-1}}\setminus E_{s_i}]$ for all $i=1,\ldots ,N$.

Since $E_0$ is bounded and $f$ is continuous, there exists $N>1$ such that

5.8 $$\begin{aligned} f\Big (\tfrac{\textrm{d}_{E_0}(x)}{s_i}\Big ) \ge f\Big (\tfrac{\textrm{d}_{E_0}(x)}{s_{i-1}}\Big ) - \epsilon ,\quad x\in E_0,\quad i=1,\ldots ,N. \end{aligned}$$

Thus, by the minimality of $E_{s_i}$ and (5.8)

$$\begin{aligned} P_\varphi (E_{s_{i-1}}\cap G) - P_\varphi (E_{s_i}\cap G) = P_\varphi (E_{s_i}\cup G_i) - P_\varphi (E_{s_i}) \\ \ge \int _{G_i} f\left( \tfrac{\textrm{d}_{E_0}}{s_i}\right) ~dx \ge \int _{G_i} f\left( \tfrac{\textrm{d}_{E_0}}{s_{i-1}}\right) ~dx - \epsilon |G_i|. \end{aligned}$$

By (5.2)

$$\begin{aligned} f\Big (\tfrac{\textrm{d}_{E_0}(x)}{s_{i-1}}\Big ) = f\Big (\tfrac{-\textrm{sd}_{E_0}(x)}{s_{i-1}}\Big ) \ge f\Big (\tfrac{-\textrm{sd}_{E_{s_{i-1}}}(x) + f^{-1}(\delta )s_{i-1}}{s_{i-1}}\Big ) \ge \delta \quad \text {for }x\in E_{s_{i-1}}, \end{aligned}$$

and therefore

$$\begin{aligned} P_\varphi (E_{s_{i-1}}\cap G) - P_\varphi (E_{s_i}\cap G) \ge (\delta -\epsilon )|G_i|,\quad i=1,\ldots ,N. \end{aligned}$$

Summing these inequalities we get

5.9 $$\begin{aligned} P_\varphi (E_{\underline{s}}\cap G) - P_\varphi (E_{\overline{s}}\cap G) \ge (\delta -\epsilon )|G\cap [E_{\underline{s}} \setminus E_{\overline{s}}]|. \end{aligned}$$

Moreover, since $E_{\underline{s}}\subset E_0$ is mean convex and $E_0$ is $\delta$-mean convex, applying Proposition 5.1 (c) twice (first with $E_{\underline{s}}$ and $\delta =0$ and next with $E_0$ and $\delta$) we obtain

$$\begin{aligned} P_\varphi (E_{\underline{s}} \cap G) = P_\varphi (E_{\underline{s}} \cap [E_0\cap G]) \le P_\varphi (E_0\cap G) \le P_\varphi (G) - \delta |G\setminus E_0|. \end{aligned}$$

Inserting this in (5.9)

$$\begin{aligned} P_\varphi (G) - \delta |G\setminus E_0| \ge P_\varphi (E_{\overline{s}}\cap G) + (\delta -\epsilon )|G\cap [E_{\underline{s}} \setminus E_{\overline{s}}]|, \end{aligned}$$

and hence, letting $\epsilon ,\underline{s} \rightarrow 0^+$ and using step 2 we get

$$\begin{aligned} P_\varphi (G) \ge P_\varphi (E_{\overline{s}}\cap G) + \delta |G \setminus E_{\overline{s}}|. \end{aligned}$$

Finally, letting $\overline{s}\searrow \tau$ and $\overline{s}\nearrow \tau ,$ using step 3 and the $L^1$-lower semicontinuity of $P_\varphi ,$ we get

$$\begin{aligned} P_\varphi (G) \ge P_\varphi (E_{\tau *}\cap G) + \delta |G \setminus E_{\tau *}| \quad \text {and}\quad P_\varphi (G) \ge P_\varphi (E_\tau ^* \cap G) + \delta |G \setminus E_\tau ^*|. \end{aligned}$$

Thus, both $E_\tau ^*$ and $E_{\tau *}$ are $\delta$-mean convex by Proposition 5.1. $\square$

### Corollary 5.4

(Mean convexity of minimizers) Let $E_0\Subset \Omega$ be $\delta$-mean convex and $\tau >0.$ Then every minimizer $E_\tau$ of ${\mathcal {F}}(\cdot ;E_0,\tau )$ is $\delta$-mean convex.

### Proof

If $\delta =0,$ the assertion follows from Lemma 5.3 (c), so we assume $\delta >0$ and consider the minimal and maximal minimizers $E_{\tau *}\subset E_\tau \subset E_\tau ^*.$ Then, for any $G\Subset \Omega$, by the $\delta$-mean convexity of $E_\tau ^*$ (Proposition 5.3),

5.10 $$\begin{aligned} P_\varphi (G) \ge P_\varphi (E_\tau ^*\cap G) + \delta |G\setminus E_\tau ^*|. \end{aligned}$$

Moreover, possibly slightly perturbing *G* we may assume that $\mathcal {H}^{n-1}(\partial ^*G\cap \partial ^*E_\tau )= 0$ so that, by the minimality of $E_\tau \subset E_\tau ^*$,

$$\begin{aligned}  &   P_\varphi (E_\tau ^*\cap G) - P_\varphi (E_\tau \cap G) =&P_\varphi (E_\tau \cup [G\cap (E_\tau ^*\setminus E_\tau )]) - P_\varphi (E_\tau ) \\  &   \quad \ge \int _{G\cap (E_\tau ^*\setminus E_\tau )} f\left( \tfrac{\textrm{d}_{E_0}}{\tau }\right) ~dx. \end{aligned}$$

Moreover, by (5.2) it follows

$$\begin{aligned} f\left( \tfrac{\textrm{d}_{E_0}}{\tau }\right) = f\Big ( \tfrac{-\textrm{sd}_{E_0}(x) }{\tau }\Big ) \ge f\Big ( \tfrac{-\textrm{sd}_{E_\tau ^*}(x) + f^{-1}(\delta )\tau }{\tau }\Big ) \ge \delta \quad \hbox { for}\ x\in E_\tau ^*, \end{aligned}$$

and hence,

$$\begin{aligned} P_\varphi (E_\tau ^*\cap G) \ge P_\varphi (E_\tau \cap G) +\delta |G\cap (E_\tau ^*\setminus E_\tau )|. \end{aligned}$$

Adding this to (5.10) we get

$$\begin{aligned} P_\varphi (G) \ge P_\varphi (E_\tau \cap G) + \delta |G\setminus E_\tau |, \end{aligned}$$

i.e., $E_\tau$ is $\delta$-mean convex. $\square$

### Proof of Theorem 1.5

Let $\{E(\tau _j,k)\}$ be a family of flat flows starting from $E_0$ and satisfying

5.11 $$\begin{aligned} \lim \limits _{j\rightarrow +\infty }\,|E(\tau _j,\left\lfloor t/\tau _j \right\rfloor ) \Delta E(t)| = 0\quad \hbox { for any}\ t\ge 0 \end{aligned}$$

for some $E(\cdot )\in \textrm{GMM}({\mathcal {F}}, E_0).$ By Lemma 5.2 (a) it follows

$$\begin{aligned} \overline{E_0}\supset \overline{E(\tau _j,1)} \supset \overline{E(\tau _j,2)} \supset \ldots \end{aligned}$$

and by Corollary 5.4 each $E(\tau _j,k)$ is $\delta$-mean convex. Hence, $t\mapsto E(\tau _j,\left\lfloor t/\tau _j \right\rfloor )$ is a nonincreasing map of $\delta$-mean convex sets. Then by (5.11) and Proposition 5.1 (d), each *E*(*t*) is $\delta$-mean convex and the map $t\mapsto E(t)$ is nonincreasing. Therefore, by the definition of mean convexity, so is $t\mapsto P_\varphi (E(t)).$ $\square$

## Consistency with Smooth Flows: Proof of Theorem 1.8

If, for an anisotropy $\varphi$, the map $\xi \mapsto \varphi (\xi ) - \lambda |\xi |$ is also an anisotropy in ${\mathbb {R}}^n$ for some $\lambda >0,$ we say $\varphi$ is elliptic.

Suppose $\varphi$ is a $C^{3+\beta }$-elliptic anisotropy, and functions $f$ and $g$ satisfy Hypothesis (H), $f\in C^\beta (\mathbb {R})$ and $g\in C^{\beta } ({\mathbb {R}}^n) \cap L^\infty ({\mathbb {R}}^n)$, for some $\beta \in (0,1]$.

### Definition 6.1

(*Stable smooth flow*)

- A $C^1$-in time family $\{S(t)\}_{t\in [0,T^\dag )}$ of $C^2$-subsets of ${\mathbb {R}}^n$ is called a generalized smooth power mean curvature flow with driving force $g$ starting from $S_0$, if
  $$\begin{aligned} {\left\{ \begin{array}{ll} f(v_{S(t)}(x)) = - (\kappa _{S(t)}^\varphi (x) + g(x)) &  \hbox {for} \,\, t\in (0,T^\dag ) \,\, \hbox {and} \,\, x\in \partial S(t),\\ S(0)=S_0, \end{array}\right. } \end{aligned}$$
  where as usual $v_{S(t)}$ and $\kappa _{S(t)}^\varphi$ are the normal velocity and the anisotropic mean curvature of $\partial S(t)$, respectively.
- The family $\{S(t)\}_{t\in [0,T^\dag )}$ is called *stable* if for any $T\in (0,T^\dag )$ there are $\rho =\rho (T)>0$ and $\sigma = \sigma (T)>0$ such that for any $a\in [0,T)$ there exist families $L^\pm [r,s,a,t]$ for $r\in [0,\rho ],$ $s\in [0,\sigma ]$ and $t\in [a,T]$ of $C^{2+\beta }$-subsets of ${\mathbb {R}}^n$ smoothly depending^8^ on *r*, *s*, *a*, *t*, such that:
  $$L^\pm [0,0,a,t] = S(t)$$ for all $$t\in [a,T],$$
  $L^\pm [r,s,a,a] = \{x \in {\mathbb {R}}^n:\,\,\textrm{sd}_{S(a)}(x)<\pm (r+s)\}$ for all $r\in [0,\rho ]$ and $s\in [0,\sigma ],$
  for any $r\in [0,\rho ]$ and $s\in [0,\sigma ],$
    6.1 $$\begin{aligned} f(v_{L^\pm [r,s,a,t]}(x)) = - \kappa _{L^\pm [r,s,a,t]}^\varphi (x) - g(x) \pm s \quad \text { for } t\in [a,T]\hbox { and }x\in \partial L^\pm [r,s,a,t].\nonumber \\ \end{aligned}$$

Using the signed distance functions we can rewrite (6.1) as

$$\begin{aligned}  &   f\Big ( \tfrac{\partial }{\partial t} \textrm{sd}_{L^\pm [r,s,a,t]}(x) \Big ) = - \kappa _{L^\pm [r,s,a,t]}^\varphi (x) - g(x) \pm s \quad \text {for }t\in [a,T]\\  &   \quad \hbox { and }x\in \partial L^\pm [r,s,a,t], \end{aligned}$$

where $\kappa _{L^\pm [r,s,a,t]}^\varphi$ stands for the $\varphi$-mean curvature of $L^\pm [r,s,a,t]$.

### Proposition 6.2

(Properties of stable flows) Let $\{S(t)\}_{t\in [0,T^\dag )}$ be a stable flow starting from a bounded set $S_0$ and for $T\in (0,T^\dag )$ let $\rho ,\sigma ,$ $L^\pm [r,s,a,t]$ be as in Definition 6.1 (b). The following properties hold:

- Assume that $r', r'' \in [0, \rho ]$, $r'\le r''$ and $s', s'' \in [0, \sigma ]$, $s'\le s''$ with $r'+s'<r''+s''.$ Then
  $$\begin{aligned} L^-[r'',s'',a,t]\Subset L^-[r',s',a,t] \quad \text {and}\quad L^+[r',s',a,t]\Subset L^+[r'',s'',a,t] \end{aligned}$$
  for any $$t\in [a,T].$$
- For any $s\in (0,\sigma )$ there exists $\tau _2\in (0,T/2)$ such that for any $\tau \in (0,\tau _2),$ $r\in [0,\rho ]$ and^9^$t\in [a+\tau ,T]$
  $$\begin{aligned} f\Big (\tfrac{ \textrm{sd}_{L^+[r,s,a,t-\tau ]}}{\tau }\Big ) > - \kappa _{L^+[r,s,a,t]}^\varphi - g+ \frac{s}{2} \quad \text {on }\partial L^+[r,s,a,t] \end{aligned}$$
  and
  $$\begin{aligned} f\Big (\tfrac{ \textrm{sd}_{L^-[r,s,a,t-\tau ]}}{\tau }\Big ) < - \kappa _{L^-[r,s,a,t]}^\varphi - g- \frac{s}{2} \quad \text {on }\partial L^-[r,s,a,t]. \end{aligned}$$
- There exists $t^* = t^*(T, \rho , \sigma , S(\cdot )) \in (0,\rho /64)$ such that
  $$\begin{aligned}  &   L^-[\rho ,s,a,a+t'] \subset L^-[\rho /2+t',s,a,a] \quad \text {and}\\  &   L^+[\rho /2-t',s,a,a]\subset L^+[\rho ,s,a,a+t'] \end{aligned}$$
  for all $s\in [0,\sigma ],$ $a\in [0,T)$ and $t'\in [0,t^*]$ with $a+t'\le T.$
- There exists a continuous increasing function $h:\mathbb {R}_0^+\rightarrow \mathbb {R}_0^+$ with $h(0)=0$ such that for all $s\in [0,\sigma ],$ $a\in [0,T),$ $t\in [a,T]$
  $$\begin{aligned} \sup _{x\in \partial L^\pm [0,s,a,t]}\,\textrm{dist}(x,\partial L^\pm [0,0,a,t]) \le h(s). \end{aligned}$$

### Proof

By smoothness, the family $\{S(t)\}$ is uniformly bounded. Therefore, assertion (a) follows from the strong comparison principle (see e.g. [27, Chapter 2]). The remaning assertions follow from the smooth dependence of *L* on its variables, the Hölder regularity of $f$ and the continuity and boundedness of $g$. We refer to [2, Corollary 7.2] for more details in the mean curvature setting. $\square$

### Remark 6.3

As in the standard mean curvature case, using the Hamilton-type arguments [27, Chapter 2], one can show the following comparison principle: if $A_0\Subset B_0,$ and $\{A(t)\}_{t\in [0,T)}$ and $\{B(t)\}_{t\in [0,T)}$ are generalized smooth power mean curvature flows starting from $A_0$ and $B_0,$ respectively, then $A(t)\Subset B(t)$ for any $t\in [0,1).$

Let $E(\cdot )$ be any $\textrm{GMM}$ starting from the smooth bounded set $E_0=S_0$ and let a sequence $\tau _j\rightarrow 0^+$ and flat flows $E(\tau _j,k)$ be such that

6.2 $$\begin{aligned} \lim _{j\rightarrow +\infty }\,|E(\tau _j,\left\lfloor t/\tau _j \right\rfloor ) \Delta E(t)| = 0\quad \hbox { for all}\ t\ge 0. \end{aligned}$$

In order to show Theorem 1.8, it suffices to prove that for any $T\in (0,T^\dag )$

6.3 $$\begin{aligned} E(t) = S(t),\quad t\in [0,T). \end{aligned}$$

Given $T\in (0,T^\dag )$ and $a\in [0,T),$ let $\rho ,\sigma >0$ and $L^\pm [r,s,a,t]$ be as in Definition 6.1 (b), and given $s\in [0,\sigma ],$ let $\tau _2:=\tau _2(s)$ be given by Proposition 6.2 (b). Let also $t^*$ be as in Proposition 6.2 (c).

The proof of (6.3) basically follows applying inductively the following auxiliary lemma.

### Lemma 6.4

Assume that $a \in [0,T)$ and $s\in (0,\sigma )$ are such that

6.4 $$\begin{aligned} L^-[0,s,a,a] \subset E(\tau _j,k_0)\subset L^+[0,s,a,a], \end{aligned}$$

where $k_0:=\left\lfloor a/\tau _j \right\rfloor .$ Then there exists $\bar{t}\in (0,t^*]$ depending only on $t^*$ and $\rho$, such that

6.5 $$\begin{aligned} L^-[0,s,a,a+k\tau _j]\subset E(\tau _j,k_0+k)\subset L^+[0,s,a,a+k\tau _j] \end{aligned}$$

for all $j\ge 1$ with $\tau _j\in (0,\tau _2(s))$ and $k=0,1,\ldots ,\left\lfloor {\bar{t}}/\tau _j \right\rfloor$ provided that $a+k\tau _j<T.$ Moreover, if $a+{\bar{t}}<T,$ for any $s\in (0,\sigma ]$ with $h(2s)<\sigma /4$ there exists $j(s)>1$ such that

6.6 $$\begin{aligned} L^-[0,4h(2s),a+{\bar{t}},a+{\bar{t}}] \subset E(\tau _j,k_0+\bar{k}_j) \subset L^+[0,4h(2s),a+{\bar{t}},a+{\bar{t}}], \end{aligned}$$

whenever $$j>j(s)$$ and $${\bar{k}}_j:=\left\lfloor {\bar{t}}/\tau _j \right\rfloor .$$

### Proof

We closely follow the arguments of the proof of the consistency in [2]. In view of (6.4) and Proposition 6.2 (a)

6.7 $$\begin{aligned} L^-[\rho /64,s,a,a] \subset L^-[0,s,a,a] \subset E(\tau _j,k_0) \subset L^+[0,s,a,a] \subset L^+[\rho /64,s,a,a]. \nonumber \\ \end{aligned}$$

Thus, by the definition of $L^\pm [\rho ,s,a,a]$

$$\begin{aligned} {\left\{ \begin{array}{ll} B_{\rho /64}(x)\subset E(\tau _j,k_0) &  \hbox { if}\ x\in L^-[\rho /64,s,a,a],\\ B_{\rho /64}(x)\cap E(\tau _j,k_0) = \emptyset &  \text {if }x\notin L^+[\rho /64,s,a,a]. \end{array}\right. } \end{aligned}$$

Thus, applying Theorem 4.3 we find a constant $C_8>1$ depending only on *n*, $\varphi$, $f$, $\rho$ and $\Vert g\Vert _\infty$ such that

6.8 $$\begin{aligned} {\left\{ \begin{array}{ll} B_{\rho /64 - C_8 i\tau _j}(x)\subset E(\tau _j,k_0+i) &  \text {if }x\in L^-[\rho /64,s,a,a],\\ B_{\rho /64-C_8i\tau _j}(x)\cap E(\tau _j,k_0+i) = \emptyset &  \text {if } x\notin L^+[\rho /64,s,a,a] \end{array}\right. } \end{aligned}$$

for all $0\le i\tau _j \le \frac{\rho }{128C_8}.$ By (6.7) and (6.8) for such *i*

6.9 $$\begin{aligned} L^-[\rho /32-C_{8}i\tau _j,s,a,a ] \subset E(\tau _j,k_0+i) \subset L^+[\rho /32-C_{8}i\tau _j,s,a,a]. \end{aligned}$$

Now, using the inequality

$$\begin{aligned} \frac{\rho }{32} - C_{8}i\tau _j \le \frac{\rho }{2} - i\tau _j \end{aligned}$$

(recall that $C_8>1$) and the definition of $L^\pm [r,s,a,a]$ in (6.9), we find

6.10 $$\begin{aligned} L^-[\rho /2-i\tau _j,s,a,a] \subset E(\tau _j,k_0+i) \subset L^+[\rho /2-i\tau _j,s,a,a]. \end{aligned}$$

If $i\tau _j\le t^*,$ by Proposition 6.2 (c) and (6.10) we get

6.11 $$\begin{aligned} L^-[\rho ,s,a,a+i\tau _j] \subset E(\tau _j,k_0+i) \subset L^+[\rho ,s,a,a+i\tau _j]. \end{aligned}$$

Let us define

$$\begin{aligned} {\bar{t}}:=\min \Big \{t^*, \tfrac{\rho }{128C_{8}}\Big \}. \end{aligned}$$

By (6.11)

$$\begin{aligned} L^-[\rho ,s,a,a+i\tau _j ] \subset E(\tau _j,k_0+i) \subset L^+[\rho ,s,a,a+i\tau _j],\quad i=0,1,\ldots ,\left\lfloor {\bar{t}}/\tau _j \right\rfloor , \end{aligned}$$

provided $a+i\tau _j<T.$ We claim that for any $j>1$ with $\tau _j\in (0,\tau _2(s))$ there holds

6.12 $$\begin{aligned} L^-[0,s,a,a+i\tau _j] \subset E(\tau _j,k_0+i) \subset L^+[0,s,a, a+i\tau _j],\quad i=0,1,\ldots ,\left\lfloor {\bar{t}}/\tau _j \right\rfloor ,\nonumber \\ \end{aligned}$$

with $a+i\tau _j<T.$ Indeed, let

$$\begin{aligned} {\bar{r}}:= &   \inf \Big \{r\in [0,\rho ]:\,\, E(\tau _j,k_0+i) \subset L^+[r,s,a,a+i\tau _j],\\  &   i=0,\ldots ,\left\lfloor \bar{t}/\tau _j \right\rfloor ,\,a+i\tau _j<T\Big \}. \end{aligned}$$

To prove the claim we need to show that

6.13 $$\begin{aligned} {\bar{r}}=0. \end{aligned}$$

In view of (6.11) the infimum is taken over a nonempty set. By contradiction, assume that ${\bar{r}}>0.$ By the continuity of $L^+[\cdot ,s,a,a+i\tau _j]$ at $r={\bar{r}},$ there exists the smallest integer $k\le \left\lfloor {\bar{t}}/\tau _j \right\rfloor$ (clearly, $k>0$ by (6.7) and the assumption ${\bar{r}}>0$) for which

6.14 $$\begin{aligned} \partial E(\tau _j,k_0+k) \cap \partial L^+[{\bar{r}},s,a,a+k\tau _j] \ne \emptyset . \end{aligned}$$

Moreover, by the minimality of *k* and the definition of ${\bar{r}}$,

$$\begin{aligned} E(\tau _j,k_0+k-1)\subset L^+[{\bar{r}},s,a,a+(k-1)\tau _j],\quad E(\tau _j,k_0+k)\subset L^+[{\bar{r}},s,a,a+k\tau _j]. \end{aligned}$$

By Proposition 6.2 (b) (recall that $k_0=\left\lfloor a/\tau _j \right\rfloor$) it follows

$$\begin{aligned} f\Big (\tfrac{ \textrm{sd}_{L^+[\bar{r},s,a,a+k\tau _j-\tau _j]}}{\tau _j}\Big ) > - \kappa _{L[\bar{r},s,a,a+k\tau _j]}^\varphi - g+ \frac{s}{2} \quad \text {on }\partial L[{\bar{r}},s,a,a+k\tau _j]. \end{aligned}$$

Thus, applying Lemma B.1 (a) with $E:=F(\tau _j,k_0+k-1),$ $E_\tau :=F(\tau _j,k_0+k),$ $F:=L^+[\bar{r},s,a,a+(k-1)\tau _j]$ and $F_\tau :=L^+[{\bar{r}},s,a,a+k\tau _j]$ we obtain

$$\begin{aligned} \partial E(\tau _j,k_0+k) \cap \partial L^+[{\bar{r}},s,a,a+k\tau _j] = \emptyset , \end{aligned}$$

which contradicts (6.14). Thus (6.13) is proven. A similar contradiction argument based on Lemma B.1 (b) and Proposition 6.2 (b) for $s<0$ shows the validity of (6.12). This concludes the proof of (6.5).

Now, let us prove (6.6). By construction $L^-[0,2\,s,a,a]\Subset L^-[0,s,a,a]$ and $L^+[0,s,a,a]\Subset L^+[0,2\,s,a,a].$ Thus, by the comparison principle in Remark 6.3, $L^-[0,2s,a,t]\Subset L^-[0,s,a,t]$ and $L^+[0,s,a,t]\Subset L^+[0,2\,s,a,t]$ for all $t\in [a,T].$ Since $L^\pm [0,\cdot ,\cdot ,\cdot ]$ varies in a continuous manner, there is $j(s)>1$ such that, for any $j>j(s)$,

6.15 $$\begin{aligned}  &   L^-[0,2s,a,a+{\bar{t}}]\subset L^-[0,s,a,a+{\bar{k}}_j\tau _j] \nonumber \\  &   \quad \subset E(\tau _j,{\bar{k}}_j) \subset L^+[0,s,a,a+\bar{k}_j\tau _j] \subset L^+[0,2s,a,a+{\bar{t}}], \end{aligned}$$

where we recall that ${\bar{k}}_j:=\left\lfloor {\bar{t}}/\tau _j \right\rfloor$, and we used ${\bar{k}}_j\tau _j = \left\lfloor {\bar{t}}/\tau _j \right\rfloor \tau _j\nearrow \bar{t}$ as $j\rightarrow +\infty$. Let the function $h$ be given by Proposition 6.2 (d), so that

$$\begin{aligned} \max _{x\in \partial L^\pm [0,2s,a,a+{\bar{t}}]}\textrm{dist}(x,\partial L^\pm [0,0,a,a+\bar{t}]) \le h(2s). \end{aligned}$$

Since, by the definition of $L^\pm$, we have $L^\pm [0,0,a,a+{\bar{t}}] = E(a+{\bar{t}})= L^\pm [0,0,a+{\bar{t}},a+{\bar{t}}]$ and, by the definition of $h$,

$$\begin{aligned} \textrm{dist}(\partial L^\pm [0,h(2s),a+{\bar{t}},a+{\bar{t}}], \partial L^\pm [0,0,a+{\bar{t}},a+{\bar{t}}]) = h(2s), \end{aligned}$$

it follows that

$$\begin{aligned}  &   L^-[0,4h(2s),a+{\bar{t}},a+{\bar{t}}]\subset L^-[0,2s,a,a+\bar{t}] \quad \text {and}\\  &   \quad L^+[0,2s,a,a+{\bar{t}}] \subset L^+[0,4h(2s),a+{\bar{t}},a+{\bar{t}}]. \end{aligned}$$

Using this and (6.15) we get

$$\begin{aligned} L^-[0,4h(2s),a+{\bar{t}},a+{\bar{t}}] \subset E(\tau _j,k_0+\bar{k}_j) \subset L^+[0,4h(2s),a+{\bar{t}},a+{\bar{t}}]. \end{aligned}$$

$$\square $$

### Proof of Theorem 1.8

Let ${\bar{t}}$ be given by Lemma 6.4,

$$\begin{aligned} N:=\left\lfloor T/{\bar{t}} \right\rfloor + 1 \end{aligned}$$

and let $\sigma _0\in (0,\sigma /16)$ be such that the numbers

$$\begin{aligned} \sigma _l=4h(2\sigma _{l-1}),\quad l=1,\ldots ,N, \end{aligned}$$

satisfy $\sigma _l\in (0,\sigma /16).$ By the monotonicity and continuity of *h* together with $h(0)=0,$ and the finiteness of *N*,  such a choice of $\sigma _0$ is possible (indeed, it is enough to observe that if $\sigma _0\rightarrow 0,$ then all $\sigma _l\rightarrow 0$).

Fix any $s\in (0,\sigma _0)$ and let

$$\begin{aligned} a_0(s):=s,\quad a_l(s):=4h(2a_{l-1}(s)),\quad l=1,\ldots ,N. \end{aligned}$$

Note that $a_l(s)\in (0,\sigma _l).$ In particular, the numbers $j(a_l(s))$ given by the last assertion of Lemma 6.4, are well-defined. Let also

$$\begin{aligned} j_l^s:=\max \{j\ge 1:\,\, \tau _j\notin (0,\tau _2(a_l(s)))\} \end{aligned}$$

and set

$$\begin{aligned} j_s:= 1 + \max _{l=0,\ldots ,N}\,\max \{ j(a_l(s)),j_l^s\}. \end{aligned}$$

By the definition of $L^\pm ,$

$$\begin{aligned} L^-[0,s,0,0] \subset S(0)=E_0=E(\tau _j,0) \subset L^+[0,s,0,0] \end{aligned}$$

for all $j>j_s$ (basically, this is true for all *j*). Therefore, by Lemma 6.4 applied with $a=0$ (so that $k_0:=\left\lfloor a/\tau _j \right\rfloor =0$) we find

$$\begin{aligned} L^-[0,s,0,k\tau _j]\subset E(\tau _j,k) \subset L^+[0,s,0,k\tau _j],\quad k=0,1,\ldots ,{\bar{k}}_j, \end{aligned}$$

where ${\bar{k}}_j:=\left\lfloor {\bar{t}}/\tau _j \right\rfloor .$ Moreover, since $s\in (0,\sigma _0)$, by the last assertion of Lemma 6.4 and the definition of $a_l(s)$,

$$\begin{aligned} L^-[0,a_1(s),{\bar{t}},{\bar{t}}]\subset E(\tau _j,{\bar{k}}_j) \subset L^+[0,a_1(s),{\bar{t}}, {\bar{t}}] \end{aligned}$$

for all $j\ge j_s.$ Hence, we can reapply Lemma 6.4 with $s:=a_1(s),$ $a={\bar{t}}$ and $k_0={\bar{k}}_j,$ to find

$$\begin{aligned} L^-[0,a_1(s),{\bar{t}},{\bar{t}} + k\tau _j]\subset E(\tau _j,{\bar{k}}_j+k) \subset L^+[0,a_1(s),{\bar{t}},{\bar{t}}+ k\tau _j],\quad k=0,1,\ldots ,\bar{k}_j. \end{aligned}$$

In particular, since $j> j_s> j(a_1(s)),$ again by the last assertion of Lemma 6.4 we deduce

$$\begin{aligned} L^-[0,a_2(s),2{\bar{t}},2{\bar{t}}]\subset E(\tau _j,2{\bar{k}}_j) \subset L^+[0,a_2(s),2{\bar{t}},2{\bar{t}}]. \end{aligned}$$

Repeating this argument at most *N* times, for all $j> j_s$ we find

6.16 $$\begin{aligned} L^-[0,a_l(s),l{\bar{t}},l{\bar{t}} + k\tau _j]\subset E(\tau _j,l{\bar{k}}_j+k) \subset L^+[0,a_l(s),l{\bar{t}},l{\bar{t}} + k\tau _j],\quad k=0,1,\ldots ,{\bar{k}}_j \nonumber \\ \end{aligned}$$

whenever $$l=0,\ldots ,N$$ with $$l{\bar{t}} + k\tau _j < T.$$

Now, take any $t\in (0,T),$ and let $l:=\left\lfloor t/{\bar{t}} \right\rfloor$ and $k=\left\lfloor t/\tau _j \right\rfloor - l{\bar{k}}_j$, so that $l{\bar{k}}_j + k = \left\lfloor t/\tau _j \right\rfloor .$ By means of *l* and *k*,  as well as the definition of ${\bar{k}}_j$, we may represent (6.16) as

6.17 $$\begin{aligned}  &   L^-\Big [0,a_l(s),l{\bar{t}}, l{\bar{t}} + \tau _j\left\lfloor \tfrac{t}{\tau _j} \right\rfloor - l\tau _j\left\lfloor \tfrac{{\bar{t}}}{\tau _j} \right\rfloor \Big ]\nonumber \\  &   \quad \subset E\Big (\tau _j, \left\lfloor \tfrac{t}{\tau _j} \right\rfloor \Big ) \subset L^+\Big [0,a_l(s),l{\bar{t}}, l{\bar{t}} + \tau _j\left\lfloor \tfrac{t}{\tau _j} \right\rfloor - l\tau _j\left\lfloor \tfrac{\bar{t}}{\tau _j} \right\rfloor \Big ] \end{aligned}$$

for all $j>j_s.$ Since

$$\begin{aligned} \lim \limits _{j\rightarrow +\infty } \Big (l{\bar{t}} + \tau _j\left\lfloor \tfrac{t}{\tau _j} \right\rfloor - l\tau _j\left\lfloor \tfrac{\bar{t}}{\tau _j} \right\rfloor \Big ) = t, \end{aligned}$$

by the continuous dependence of $L^\pm$ on its parameters, as well as the convergence (6.2) of the flat flows, letting $j\rightarrow +\infty$ in (6.17) we obtain

6.18 $$\begin{aligned} L^-[0,a_l(s),l{\bar{t}}, t]\subset E(t) \subset L^+[0,a_l(s),l{\bar{t}}, t], \end{aligned}$$

where, due to the $L^1$-convergence, the inclusions in (6.18) hold possibly up to some negligible set. Now, we let $s\rightarrow 0^+$ and recalling that $a_l(s)\rightarrow 0$ (by the continuity of *h* and assumption $h(0)=0$), from (6.18) we deduce

$$\begin{aligned} L^-[0,0,l{\bar{t}},t]\subset E(t) \subset L^+[0,0,l{\bar{t}},t]. \end{aligned}$$

Now, recalling $L^\pm [0,0,a,t]=S(t)$ for $t\in [a,T]$ we get

$$\begin{aligned} E(t)=L^\pm [0,0,l{\bar{t}},t] = S(t). \end{aligned}$$

$$\square $$

## Evolution of Convex Sets: Proof of Theorem 1.6

In this section we prove Theorem 1.6; thus we assume $\varphi$ is Euclidean, $g\equiv 0$ and $f(r)= \textrm{sign}(r)|r|^\alpha$ for some $\alpha >0$. We need the following result, proven in [30].

### Theorem 7.1

Let $E_0\subset {\mathbb {R}}^n$ be a bounded $C^{2+\beta }$-convex set for some $\beta \in (0,1]$. Then there exist $T^*>0$ and a unique $C^1$-in time flow $\{E(t)\}_{t\in [0,T^*)}$ starting from $E_0$, such that each *E*(*t*) is $C^2,$ convex and

$$\begin{aligned} v= - \kappa ^{1/\alpha } \quad \hbox { on}\ \partial E(t) \end{aligned}$$

for all $t\in [0,T^*).$ Moreover, $\{E(t)\}$ is stable in the sense of Definition 6.1 and $|E(t)|\rightarrow 0$ as $t\nearrow T^*.$

### *Proof of Theorem 1.6*(i)

If $\textrm{Int}({{{\mathcal {K}}}_0})=\emptyset ,$ then by convexity, $|{{\mathcal {K}}}_0|=0,$ and we are done. Otherwise, since $g\equiv 0$, translating if necessary, we assume that $\textrm{Int}({{{\mathcal {K}}}_0})$ contains the origin. Suppose that $\textrm{GMM}({\mathcal {F}},\mathcal {S}^*, {{\mathcal {K}}}_0)$ contains at least two different $\textrm{GMM}$’s, say, ${{\mathcal {K}}}'(\cdot )$ and ${{\mathcal {K}}}''(\cdot )$, so that there exist $T>0$ and $\epsilon _0>0$ such that

7.1 $$\begin{aligned} |{{\mathcal {K}}}'(T)\Delta {{\mathcal {K}}}''(T)|\ge 2\epsilon _0. \end{aligned}$$

Fix any $\lambda \in (0,1)$ sufficiently close to 1 in such a way that

7.2 $$\begin{aligned} |\lambda ^{-1}{{\mathcal {K}}}'(\lambda ^{1/\alpha } T) \setminus \lambda {{\mathcal {K}}}'(\lambda ^{-1/\alpha } T)|<\epsilon _0,\quad |\lambda ^{-1}{{\mathcal {K}}}''(\lambda ^{1/\alpha } T) \setminus \lambda {{\mathcal {K}}}''(\lambda ^{-1/\alpha } T)| <\epsilon _0. \nonumber \\ \end{aligned}$$

Let us define $E_0^\lambda :=\lambda {{\mathcal {K}}}_0$ and $F_0^\lambda := \lambda ^{-1}{{\mathcal {K}}}_0$, so that $E_0^\lambda \Subset {{\mathcal {K}}}_0\Subset F_0^\lambda .$ Now, we choose smooth convex sets $P_0$ and $Q_0$ such that

$$\begin{aligned} E_0^\lambda \Subset P_0\Subset {{\mathcal {K}}}_0\Subset Q_0\Subset F_0^\lambda . \end{aligned}$$

If we define the corresponding flat flows then, by Corollary B.4,

7.3 $$\begin{aligned} E^\lambda (\tau ,k) \Subset P(\tau ,k)\Subset C(\tau ,k)\Subset Q(\tau ,k)\Subset F^\lambda (\tau ,k) \end{aligned}$$

for any $\tau >0$ and $k\ge 0$ with $|E^\lambda (\tau ,k)|>0.$

Since $P_0$ and $Q_0$ are smooth and convex, by Theorem 7.1 the corresponding smooth flows exist and disappear at a maximal time (however, GMM starting from them exists for all times). In particular, by consistency (Theorem 1.8) both $\textrm{GMM}({\mathcal {F}},\mathcal {S}^*,P_0)$ and $\textrm{GMM}({\mathcal {F}},\mathcal {S}^*,Q_0)$ are singletons, say, $\{P(\cdot )\}$ and $\{Q(\cdot )\}.$

Let $\tau _j'\searrow 0$ and $\tau _j''\searrow 0$ be sequences for which

$$\begin{aligned} |{{\mathcal {K}}}(\tau _j',\left\lfloor t/\tau _j' \right\rfloor )\Delta {{\mathcal {K}}}'(t)| \rightarrow 0 \quad \text {and}\quad |{{\mathcal {K}}}(\tau _j'',\left\lfloor t/\tau _j'' \right\rfloor )\Delta {{\mathcal {K}}}''(t)| \rightarrow 0 \end{aligned}$$

as $j\rightarrow +\infty$ for all $t\ge 0.$ In view of Corollary 4.2, as $j \rightarrow +\infty$ we have

$$\begin{aligned} |E^\lambda (\tau _j',\left\lfloor t/\tau _j' \right\rfloor )\Delta [\lambda {{\mathcal {K}}}'(\lambda ^{-1/\alpha } t)]| \rightarrow 0 \quad \text {and}\quad |F^\lambda (\tau _j',\left\lfloor t/\tau _j' \right\rfloor )\Delta [\lambda ^{-1}{{\mathcal {K}}}''(\lambda ^{-1/\alpha } t)]| \rightarrow 0 \end{aligned}$$

and

$$\begin{aligned} |E^\lambda (\tau _j'',\left\lfloor t/\tau _j'' \right\rfloor )\Delta (\lambda {{\mathcal {K}}}'(\lambda ^{-1/\alpha } t))| \rightarrow 0 \quad \text {and}\quad |F^\lambda (\tau _j'',\left\lfloor t/\tau _j'' \right\rfloor )\Delta [\lambda ^{-1}{{\mathcal {K}}}''(\lambda ^{1/\alpha } t)]| \rightarrow 0. \end{aligned}$$

Now, applying (7.3) with $\tau _j'$ we deduce

7.4 $$\begin{aligned} \lambda {{\mathcal {K}}}'(\lambda ^{-1/\alpha } t) \subset P(t) \subset {{\mathcal {K}}}'(t) \subset Q(t)\subset \lambda ^{-1}{{\mathcal {K}}}'(\lambda ^{1/\alpha } t)\quad \text {for all } t\in [0,T], \end{aligned}$$

and appyling it with $\tau _j''$ we deduce

7.5 $$\begin{aligned} \lambda {{\mathcal {K}}}''(\lambda ^{-1/\alpha } t) \subset P(t) \subset {{\mathcal {K}}}''(t)\subset Q(t)\subset \lambda ^{-1}{{\mathcal {K}}}''(\lambda ^{1/\alpha } t) \quad \text {for all }t\in [0,T]. \end{aligned}$$

By (7.2)

$$\begin{aligned} |Q(T)\setminus P(T)| \le |[ \lambda ^{-1}C'(\lambda ^{1/\alpha } T)]\setminus [\lambda C'(\lambda ^{-1/\alpha } T)]| \le \epsilon _0. \end{aligned}$$

However, in view of (7.4) and (7.5) as well as of (7.1),

$$\begin{aligned} 2\epsilon _0\le |{{\mathcal {K}}}''(T)\Delta {{\mathcal {K}}}'(T)| \le |Q(T)\setminus P(T)| \le \epsilon _0, \end{aligned}$$

a contradiction. $$\square $$

### *Proof of Theorem 1.6*(ii)

By the Kuratowski convergence of $(\partial {{\mathcal {K}}}_{0 h})$ and comparison principles, given $\lambda \in (0,1),$ as in the proof of (i),

$$\begin{aligned} \lambda {{\mathcal {K}}}(t\lambda ^{-1/\alpha }) \subset {{\mathcal {K}}}_h(t)\subset \lambda ^{-1}{{\mathcal {K}}}(t\lambda ^{1/\alpha })\quad \text {for all } t\ge 0, \end{aligned}$$

provided $h\in {\mathbb {N}}$ is large enough depending only on $\lambda .$ Since the sequence $(P({{\mathcal {K}}}_h(t)))$ is bounded (by the supremum of $P({{\mathcal {K}}}_{0 h})$), up to a not relabelled subsequence, ${{\mathcal {K}}}_{h}(t) \rightarrow {{\mathcal {K}}}'(t)$ in $L^1({\mathbb {R}}^n)$ as $i\rightarrow +\infty .$ Then

$$\begin{aligned} \lambda {{\mathcal {K}}}(t\lambda ^{-1/\alpha }) \subset {{\mathcal {K}}}'(t)\subset \lambda ^{-1}{{\mathcal {K}}}(t\lambda ^{1/\alpha })\quad \text {for all } t\ge 0. \end{aligned}$$

Now, letting $\lambda \rightarrow 1$, we get ${{\mathcal {K}}}'(t)={{\mathcal {K}}}(t),$ i.e., the limit of $({{\mathcal {K}}}_h)$ is independent of the subsequence. Thus, the thesis follows. $\square$

## Minimizing Movements in the Class $\mathrm {Conv_b}({\mathbb {R}}^n)$: Proof of Theorem 1.7

In this section we suppose $g\equiv 0$. Motivated by Conjecture 1.2, we study $\textrm{GMM}$ in the class $\mathrm {Conv_b}({\mathbb {R}}^n)$. Notice that, due to the convexity constraint, the first variation of ${\mathcal {F}}$ is nonlocal, and thus, in general we cannot write a pointwise Euler-Lagrange equation; therefore, the nature of $\textrm{GMM}({\mathcal {F}},\mathrm {Conv_b}({\mathbb {R}}^n), {{\mathcal {K}}}_0)$ seems not clear. Moreover, to prove Theorem 1.7 we cannot apply the techniques used in the proof of Theorem 1.4, based on cutting and filling with balls (because they lead to the loss of convexity).

### Proof of Theorem 1.7

Let ${{\mathcal {K}}}_0\in \mathrm {Conv_b}({\mathbb {R}}^n).$ Without loss of generality we may assume that the interior of ${{\mathcal {K}}}_0$ is nonempty. By the $L_\textrm{loc}^1$-closedness of $\mathrm {Conv_b}({\mathbb {R}}^n)$ and the $L_\textrm{loc}^1$-lower semicontinuity of ${\mathcal {F}}(\cdot ;{{\mathcal {K}}},\tau )$ for any ${{\mathcal {K}}}\in \mathrm {Conv_b}({\mathbb {R}}^n),$ there exists a minimizer of ${\mathcal {F}}(\cdot ;{{\mathcal {K}}},\tau )$ in $\mathrm {Conv_b}({\mathbb {R}}^n).$ By truncation, we can readily show that every minimizer ${{\mathcal {K}}}_\tau$ satisfies ${{\mathcal {K}}}_\tau \subset {{\mathcal {K}}}.$ Now, we define flat flows $\{{{\mathcal {K}}}(\tau ,k)\};$ clearly,

8.1 $$\begin{aligned} {{\mathcal {K}}}_0={{\mathcal {K}}}(\tau ,0)\supset {{\mathcal {K}}}(\tau ,1)\supset \cdots \quad \text {and}\quad P_\varphi ({{\mathcal {K}}}_0)=P_\varphi ({{\mathcal {K}}}(\tau ,0))\ge P_\varphi ({{\mathcal {K}}}(\tau ,1)) \ge \cdots .\nonumber \\ \end{aligned}$$

For any $\tau >0,$ define

$$\begin{aligned} h_\tau (t):=P_\varphi ({{\mathcal {K}}}(\tau ,\left\lfloor t/\tau \right\rfloor )),\quad t\ge 0. \end{aligned}$$

By (8.1), $\{h_\tau \}$ are nonincreasing nonnegative functions satisfying $h_\tau (0)=P({{\mathcal {K}}}_0)$. Thus, Helly’s selection theorem implies the existence of a sequence $\tau _j\rightarrow 0^+$ and a nonincreasing function $h_0:\mathbb {R}_0^+\rightarrow \mathbb {R}_0^+$ such that $h_{\tau _j}\rightarrow h_0$ in $\mathbb {R}_0^+.$ Since $h_0$ is monotone, its discontinuity set $J\subset \mathbb {R}_0^+$ is at most countable. Let $Q\subset \mathbb {R}_0^+\setminus J$ be any countable dense set in $\mathbb {R}_0^+$. By compactness in *BV*, passing to a further not relabelled subsequence $(\tau _j)$ and using a diagonal argument, for any $s\in Q\cup J$ we can define a set ${{\mathcal {K}}}(s)\subset {\mathbb {R}}^n$ such that

$$\begin{aligned} {{\mathcal {K}}}(\tau _j,\left\lfloor s/\tau _j \right\rfloor ) \rightarrow {{\mathcal {K}}}(s)\quad \text {in } L^1({\mathbb {R}}^n)\hbox { as }j\rightarrow +\infty . \end{aligned}$$

Since each ${{\mathcal {K}}}(\tau _j,\left\lfloor s/\tau _j \right\rfloor )$ is convex, so is ${{\mathcal {K}}}(s)$ and hence

$$\begin{aligned} \lim \limits _{j\rightarrow +\infty }\,h_{\tau _j}(s) = \lim \limits _{j\rightarrow +\infty }\, P_\varphi ({{\mathcal {K}}}(\tau _j,\left\lfloor s/\tau _j \right\rfloor )) = P_\varphi ({{\mathcal {K}}}(s)) = h_0(s). \end{aligned}$$

Now, take any $0<s\in \mathbb {R}_0^+{\setminus } [Q\cup J]$, so that $h_0$ is continuous at *s*. By density of *Q* in $\mathbb {R}_0^+,$ we can choose sequences $Q\ni a_k\nearrow s$ and $Q\ni b_k\searrow s.$ By (8.1) the maps $k\mapsto {{\mathcal {K}}}(a_k)$ and $k\mapsto P_\varphi ({{\mathcal {K}}}(a_k))$ are nonincreasing and the maps $k\mapsto {{\mathcal {K}}}(b_k)$ and $k\mapsto P_\varphi ({{\mathcal {K}}}(b_k))$ are nondecreasing. Let

8.2 $$\begin{aligned} \bigcap _{k\ge 1} {{\mathcal {K}}}(a_k)=:{{\mathcal {K}}}(s)^*\supset {{\mathcal {K}}}(s)_*= \bigcup _{k\ge 1} {{\mathcal {K}}}(b_k). \end{aligned}$$

By the continuity of $h_0$ at *s*,  both $P_\varphi ({{\mathcal {K}}}(a_k))$ and $P_\varphi ({{\mathcal {K}}}(b_k))$ converge to $h_0(s),$ and therefore $P_\varphi ({{\mathcal {K}}}(s)^*) = P_\varphi ({{\mathcal {K}}}(s)_*).$ Thus, by the convexity of both ${{\mathcal {K}}}(s)_*$ and ${{\mathcal {K}}}(s)^*$, it follows

$$\begin{aligned} {{\mathcal {K}}}(s)_*={{\mathcal {K}}}(s)^*=:{{\mathcal {K}}}(s) \end{aligned}$$

and $|{{\mathcal {K}}}(a_k)\Delta {{\mathcal {K}}}(s)|,|{{\mathcal {K}}}(b_k)\Delta {{\mathcal {K}}}(s)|\rightarrow 0.$ Now, let us show that

8.3 $$\begin{aligned} {{\mathcal {K}}}(\tau _j,\left\lfloor s/\tau _j \right\rfloor ) \rightarrow {{\mathcal {K}}}(s)\quad \text {in } L^1({\mathbb {R}}^n)\hbox { as }j\rightarrow +\infty . \end{aligned}$$

Notice that

8.4 $$\begin{aligned} \limsup \limits _{j\rightarrow +\infty } \,\limsup _{k\rightarrow +\infty }\,(h_{\tau _j}(a_k) - h_{\tau _j}(b_k)) = 0, \end{aligned}$$

otherwise, $h_0$ cannot be continuous at *s*. Therefore, from the inclusion

$$\begin{aligned} {{\mathcal {K}}}(\tau _j,\left\lfloor a_k/\tau _j \right\rfloor ) \supset {{\mathcal {K}}}(\tau _j,\left\lfloor s/\tau _j \right\rfloor ) \supset {{\mathcal {K}}}(\tau _j,\left\lfloor b_k/\tau _j \right\rfloor ), \end{aligned}$$

we obtain

$$\begin{aligned} \bigcap _{k\ge 1} {{\mathcal {K}}}(\tau _j,\left\lfloor a_k/\tau _j \right\rfloor ) \supset {{\mathcal {K}}}(\tau _j,\left\lfloor s/\tau _j \right\rfloor ) \supset \bigcup _{k\ge 1} {{\mathcal {K}}}(\tau _j,\left\lfloor b_k/\tau _j \right\rfloor ). \end{aligned}$$

In view of (8.4) and the convexity of these sets, for any $\epsilon >0$ there exists $k_\epsilon >0$ such that

8.5 $$\begin{aligned} |{{\mathcal {K}}}(\tau _j,\left\lfloor a_k/\tau _j \right\rfloor ) \Delta {{\mathcal {K}}}(\tau _j,\left\lfloor s/\tau _j \right\rfloor )| = |{{\mathcal {K}}}(\tau _j,\left\lfloor a_k/\tau _j \right\rfloor ) \setminus {{\mathcal {K}}}(\tau _j,\left\lfloor s/\tau _j \right\rfloor )|<\epsilon \end{aligned}$$

provided $k>k_\epsilon .$ Moreover, in view of (8.2) and the equality $K(s)_*=K(s)^*,$ we may also assume that

8.6 $$\begin{aligned} |{{\mathcal {K}}}(a_k)\Delta {{\mathcal {K}}}(s)|<\epsilon . \end{aligned}$$

For such *k* consider the estimate

$$\begin{aligned}  &   |{{\mathcal {K}}}(\tau _j,\left\lfloor s/\tau _j \right\rfloor ) \Delta {{\mathcal {K}}}(s)| \\  &   \quad \le |{{\mathcal {K}}}(\tau _j,\left\lfloor s/\tau _j \right\rfloor ) \Delta {{\mathcal {K}}}(\tau _j,\left\lfloor a_k/\tau _j \right\rfloor )| +|{{\mathcal {K}}}(\tau _j,\left\lfloor a_k/\tau _j \right\rfloor )\Delta {{\mathcal {K}}}(a_k)| + |{{\mathcal {K}}}(a_k)\Delta {{\mathcal {K}}}(s)|. \end{aligned}$$

By (8.5) and (8.6) the first and third terms in this inequality do not exceed $\epsilon .$ Thus, letting $j\rightarrow +\infty ,$ we find

$$\begin{aligned} \limsup _{j\rightarrow +\infty }\,|{{\mathcal {K}}}(\tau _j,\left\lfloor s/\tau _j \right\rfloor ) \Delta {{\mathcal {K}}}(s)|\le 2\epsilon . \end{aligned}$$

Since $\epsilon >0$ is arbitrary, we get (8.3). Thus, by definition, the family $\{{{\mathcal {K}}}(s)\}_{s\ge 0}$ is a $\textrm{GMM}$. $\square$

Suppose (compare with the next proposition) that for any bounded convex set ${{\mathcal {K}}}_0,$ ${\mathcal {F}}(\cdot ;{{\mathcal {K}}}_0,\tau )$ admits a convex minimizer in $\mathcal {S}^*.$ In this case, the flat flows ${{\mathcal {K}}}(\tau ,k)$ consisting of those convex sets are also flat flows in $\mathrm {Conv_b}({\mathbb {R}}^n);$ by Theorem 1.6 we have ${{\mathcal {K}}}(\tau ,\left\lfloor t/\tau \right\rfloor )\rightarrow {{\mathcal {K}}}(t)$ as $\tau \rightarrow 0^+$ (because $\textrm{GMM}( {\mathcal {F}},\mathcal {S}^*,{{\mathcal {K}}}_0)$ is a singleton). Then clearly, ${{\mathcal {K}}}(\cdot )\in \textrm{GMM}({\mathcal {F}},\mathrm {Conv_b}({\mathbb {R}}^n),{{\mathcal {K}}}_0)$ (in fact is a minimizing movement). However, it seems not clear whether $\textrm{GMM}({\mathcal {F}}, \mathrm {Conv_b}({\mathbb {R}}^n),{{\mathcal {K}}}_0)$ contains other generalized minimizing movements.

### Proposition 8.1

Suppose that $\varphi$ is an elliptic $C^{3}$-anisotropy and $g\equiv 0$. Assume that $f$ is concave in $(0,+\infty ).$ Then for any bounded open convex set ${{\mathcal {K}}}_0\subset {\mathbb {R}}^n$ and $\tau >0$ the minimal and maximal minimizers of ${\mathcal {F}}(\cdot ;{{\mathcal {K}}}_0,\tau )$ in $\mathcal {S}^*$ are convex.

### Proof

We follow the notation of [11]. Since ${{\mathcal {K}}}_0$ is convex, $\textrm{d}_{{{\mathcal {K}}}_0}$ is concave in ${{\mathcal {K}}}_0,$ and hence, so is $u_0:=f(\textrm{d}_{{{\mathcal {K}}}_0}/\tau ).$ Consider

$$\begin{aligned} \mathscr {E}(v):= {\left\{ \begin{array}{ll} \displaystyle \int _{{{\mathcal {K}}}_0}\varphi ^o(Dv) + \int _{{{\mathcal {K}}}_0}\frac{(v + u_0)^2}{2} ~dx &  \text {if }v\in L^2({{\mathcal {K}}}_0)\cap BV({{\mathcal {K}}}_0),\\ +\infty &  \text {if }L^2({{\mathcal {K}}}_0)\setminus BV({{\mathcal {K}}}_0), \end{array}\right. } \end{aligned}$$

where $\phi ^o(Dv)$ is the $\phi ^o$-total variation of *v*. One checks that $\mathscr {E}$ admits a unique minimizer $v_0\in L^2({{\mathcal {K}}}_0)\cap BV({{\mathcal {K}}}_0).$ Since $\mathscr {E}$ is convex, by [7] there exists a vector field $z\in L^\infty ({{\mathcal {K}}}_0, {\mathbb {R}}^n)$ with $\textrm{div}z\in L^2({{\mathcal {K}}}_0)$, such that^10^

$$\begin{aligned} -\textrm{div}z + v_0 + u_0=0, \quad \varphi (z)\le 1,\quad \int _{{{\mathcal {K}}}_0} z\cdot Dv_0 = \int _{{{\mathcal {K}}}_0}\varphi ^o(Dv_0). \end{aligned}$$

Repeating the same arguments of [11, Lemma 5.1] we can show that for a.e. $s<0$ the set $\{v_0<s\}$ is a minimizer of

$$\begin{aligned} \mathscr {E}_s(F):=P_\varphi (F) - \int _F(v_0+s)~dx, \qquad F \subseteq {{\mathcal {K}}}_0,\quad s\in \mathbb {R}. \end{aligned}$$

If $v_0$ is twice continuously differentiable in ${{\mathcal {K}}}_0$ and $|\nabla v_0|>0$, then $v_0$ is a viscosity solution of the equation

$$\begin{aligned} -\textrm{div}\,\nabla \varphi ^o(Dv_0) + v_0 + u_0 = 0. \end{aligned}$$

Since $u_0$ is concave, using [4, Theorem 1] it follows that $v_0$ is convex. In case $v_0$ is not smooth enough, we can approximate the equation as in [11] and again get that $v_0$ is convex. In particular, $\mathscr {E}_s$ admits a convex minimizer $\{v_0<s\}.$ One can readily check that the sets

$$\begin{aligned} {{\mathcal {K}}}_*:=\bigcup _{s<0}\{v_0<s\}\quad \text {and}\quad {{\mathcal {K}}}^*:=\bigcap _{s>0}\{v_0\le s\} \end{aligned}$$

are the minimal and maximal (convex) minimizers of the functional $\mathscr {E}_0(\cdot )={\mathcal {F}}(\cdot ;{{\mathcal {K}}}_0,\tau )$. $\square$

As a corollary, we obtain the validity of Conjecture 1.1 under a restriction on $\alpha >0$.

### Corollary 8.2

(Conjecture 1.1 for $\alpha \in (0,1]$) Let $\varphi$ be the Euclidean norm, $f(r)=r^\alpha$ for $r>0$,

$$\begin{aligned} \alpha \in (0,1], \end{aligned}$$

and $g\equiv 0$. Then for any bounded convex ${{\mathcal {K}}}_0\subset {\mathbb {R}}^n,$ $\textrm{GMM}({\mathcal {F}},\mathrm {Conv_b}({\mathbb {R}}^n),{{\mathcal {K}}}_0)$ is a singleton and coincides with the unique minimizing movement in $\textrm{GMM}({\mathcal {F}},\mathcal {S}^*,{{\mathcal {K}}}_0).$

### Proof

Let ${{\mathcal {K}}}(\tau ,k)_*$ and ${{\mathcal {K}}}(\tau ,k)^*$ be flat flows in $\mathcal {S}^*$ consisting of the minimal and maximal minimizers of ${\mathcal {F}}$ (in $\mathcal {S}^*$), starting from ${{\mathcal {K}}}_0.$ By Theorem 1.6 we have

$$\begin{aligned} \lim \limits _{\tau \rightarrow 0^+}\,{{\mathcal {K}}}(\tau ,\left\lfloor t/\tau \right\rfloor )_* = {{\mathcal {K}}}(t) = \lim \limits _{\tau \rightarrow 0^+}\,{{\mathcal {K}}}(\tau ,\left\lfloor t/\tau \right\rfloor )^* \quad \hbox {in} \,\, L^1(\mathbb {R}^n) \,\,\hbox {for all} \,\,t \ge 0, \end{aligned}$$

where $\{{{\mathcal {K}}}(t)\} = \textrm{MM}({\mathcal {F}},\mathcal {S}^*,{{\mathcal {K}}}_0).$ Since by assumption $\alpha \in (0,1]$, the function $f$ is concave. By Proposition 8.1 both ${{\mathcal {K}}}(\tau ,k)_*$ and ${{\mathcal {K}}}(\tau ,k)^*$ are convex. Note that they are the minimal and maximal minimizers of ${\mathcal {F}}$ also in $\mathrm {Conv_b}({\mathbb {R}}^n).$ Therefore, $\textrm{GMM}({\mathcal {F}},\mathrm {Conv_b}({\mathbb {R}}^n),{{\mathcal {K}}}_0) = \textrm{MM}({\mathcal {F}},\mathrm {Conv_b}({\mathbb {R}}^n),{{\mathcal {K}}}_0) = \{{{\mathcal {K}}}(\cdot )\}$.$\square$

## Data Availability

There is no data associated to the paper.

## Appendix A. Volume-distance inequality

In this appendix we establish a couple of technical results needed in various proofs. In particular, the next result is crucial, and is an easy modification of the volume-distance inequality of Almgren–Taylor–Wang [2] (see also [25]).

### Lemma A.1

(Volume-distance inequality) Let $r_0>0$, and $F\in \mathcal {S}^*$ satisfy

A.1 $$\begin{aligned} P(F,B_r(x)) \ge \vartheta r^{n-1},\quad x\in \partial F,\,\,\,r\in (0,r_0], \end{aligned}$$

for some $\vartheta >0.$ Then for any measurable $E\subset {\mathbb {R}}^n$ and any $p,\ell >0$ one has

A.2 $$\begin{aligned} |E\Delta F| \le {\left\{ \begin{array}{ll} \displaystyle \frac{c p^n\ell ^n }{r_0^{n-1}}\,P_\varphi (F) + \frac{1}{f(p)} \int _{E\Delta F}f\left( \tfrac{\textrm{d}_{F}}{\ell }\right) ~dx &  \text {if }\ell>r_0, p\ell>r_0,\\ \displaystyle cp^n\ell \,P_\varphi (F) + \frac{1}{f(p)} \int _{E\Delta F}f\left( \tfrac{\textrm{d}_{F}}{\ell }\right) ~dx &  \text {if }\ell \in (0,r_0]\hbox { and }p\ell >r_0,\\ \displaystyle c p\ell \,P_\varphi (F) + \frac{1}{f(p)} \int _{E\Delta F}f\left( \tfrac{\textrm{d}_{F}}{\ell }\right) ~dx &  \text {if }p\ell \in (0,r_0], \end{array}\right. }\nonumber \\ \end{aligned}$$

where $$c:=\frac{10^n\omega _n}{c_{\varphi }\vartheta }.$$

### Proof

Define

$$\begin{aligned} {\mathcal {X}}:=\{x\in E\Delta F:\,\,\textrm{d}_F(x)\ge p\ell \},\quad {\mathcal {Y}}:=\{x\in E\Delta F:\,\,\textrm{d}_F(x)<p\ell \}. \end{aligned}$$

Since $f$ is strictly increasing, we have

$$\begin{aligned} {\mathcal {X}}= \{x\in E\Delta F:\,\, f(\textrm{d}_F(x)/\ell ) \ge f(p)\}, \end{aligned}$$

and hence, by the Chebyshev inequality

$$\begin{aligned} |{\mathcal {X}}| \le \frac{1}{f(p)}\int _P f\left( \tfrac{\textrm{d}_{F}}{\ell }\right) ~dx \le \frac{1}{f(p)} \int _{E\Delta F}f\left( \tfrac{\textrm{d}_{F}}{\ell }\right) ~dx. \end{aligned}$$

On the other hand, we cover ${\mathcal {Y}}$ with balls $B_{2p\ell }$ of radius $2p\ell$ centered at points of $\partial F.$ By the Vitali covering lemma we can take a countable subfamily $\{B_{10p\ell }'\}$ still covering ${\mathcal {Y}}$ with a pairwise disjoint family $\{B_{p\ell }'\}$.

If $\ell > r_0$ and $p\ell >r_0,$ then by the disjointness of $\{B_{r_0}'\}$ and the estimate (A.1) (applied with $r=r_0$)

$$\begin{aligned} |{\mathcal {Y}}| \le \sum _{B_{10p\ell }'} \omega _n(10p\ell )^n = \tfrac{10^n\omega _n p^n\ell ^n }{\vartheta r_0^{n-1}} \sum _{B_{r_0}'} \vartheta r_0^{n-1} \le \tfrac{10^n\omega _n p^n\ell ^n }{\vartheta r_0^{n-1}} \sum _{B_{r_0}'} P(F, B_{r_0}')\le \tfrac{10^n\omega _n p^n\ell ^n }{\vartheta r_0^{n-1}} \, P(F) \end{aligned}$$

On the other hand, if $\ell \le r_0<p\ell ,$ by disjointness of $\{B_\ell '\}$ and by (A.1) (applied with $r=\ell$)

$$\begin{aligned} |{\mathcal {Y}}| \le \sum _{B_{10p\ell }'} \omega _n(10p\ell )^n = \tfrac{10^n\omega _n p^n\ell }{\vartheta } \sum _{B_{p\ell }'} \vartheta \ell ^{n-1} \le \tfrac{10^n\omega _np \ell }{\vartheta } \sum _{B_{p\ell }'} P(F, B_{\ell }') \le \tfrac{10^n\omega _n p\ell }{\vartheta }\,P(F). \end{aligned}$$

Finally, if $p\ell \le r_0$ using the disjointness of $\{B_{p\ell }'\}$ and the estimate (A.1) (applied with $r=p\ell$)

$$\begin{aligned} |{\mathcal {Y}}| \le \sum _{B_{10p\ell }'} \omega _n(10p\ell )^n = \tfrac{10^n\omega _n p\ell }{\vartheta } \sum _{B_{p\ell }'} \vartheta p^{n-1}\ell ^{n-1} \le \tfrac{10^n\omega _n p\ell }{\vartheta } \sum _{B_{p\ell }'} P(F, B_{p\ell }') \le \tfrac{10^n\omega _n p\ell }{\vartheta }\,P(F). \end{aligned}$$

Now, use (2.1) and $|E\Delta F| = |{\mathcal {X}}| + |{\mathcal {Y}}|$ to conclude the proof of estimate (A.2). $\square$

### Lemma A.2

(Morrey-type estimate) Suppose that $g$ satisfies (1c). Then there exists $\gamma _g>0$ such that

A.3 $$\begin{aligned} \sup _{0<|A|<\omega _n \gamma _g^n} \tfrac{1}{|A|^{\frac{n-1}{n}}} \int _{A}|g|~dx \le \tfrac{ c_{\varphi }n\omega _n^{1/n}}{4}, \end{aligned}$$

where we write $\gamma _g^n=(\gamma _g)^n$.

### Proof

If $$p=+\infty ,$$ then

$$\begin{aligned} \int _{A}|g|~dx \le \Vert g\Vert _\infty |A|^{\frac{1}{n}}|A|^{\frac{n-1}{n}} \le \tfrac{c_{\varphi }n\omega _n^{1/n}}{4}|A|^{\frac{n-1}{n}} \end{aligned}$$

provided $|A|\le \omega _n \gamma _g^n$ with $\gamma _g:=\tfrac{c_{\varphi }n}{4(1+\Vert g\Vert _\infty )}.$ If $p\in (n,+\infty ),$ then by the Hölder inequality it follows

$$\begin{aligned} \int _{A}|g|~dx \le \Vert g\Vert _{L^p({\mathbb {R}}^n)}|A|^{\frac{p-1}{p}}\le \tfrac{c_{\varphi }n\omega _n^{1/n}}{4}|A|^{\frac{n-1}{n}}, \end{aligned}$$

provided $|A|\le \omega _n \gamma _g^n$ with $\gamma _g:=\omega _n^{-1/n} \big (\tfrac{c_{\varphi }n}{4(1+\Vert g\Vert _{L^p})}\big )^{\frac{p}{p-n}}.$ Finally, if $p=n,$ then by the Hölder inequality, for any $A\subset {\mathbb {R}}^n$ it follows

$$\begin{aligned} \int _{A} |g|~dx \le \Big (\int _{A} |g|^n ~dx\Big )^{\frac{1}{n}} |A|^{\frac{n-1}{n}}. \end{aligned}$$

By the absolute continuity of the Lebesgue integral, there exists $\gamma _g>0$ such that if $|A|<\omega _n \gamma _g^n,$ then

$$\begin{aligned} \Big (\int _{A} |g|^n ~dx\Big )^{\frac{1}{n}} \le \frac{c_{\varphi }n\omega _n^{1/n}}{4}. \end{aligned}$$

$$\square $$

## Appendix B. Comparison principles

The next lemma is a generalization of [2, Lemma 7.3].

### Lemma B.1

Let $\varphi$ be a $C^3$-elliptic anisotropy, $f,g$ satisfy Hypothesis (H) and let $g$ be continuous. Let $E\in \mathcal {S}^*,$ $\tau >0,$ $k\ge 0$ and $E_\tau$ be a minimizer of ${\mathcal {F}}(\cdot ; E,\tau ).$ Let *F* and $F_\tau$ be $C^{2}$-sets.

- Let $$E\subset F,$$ $$E_\tau \subset F_\tau $$ and
  B.1 $$\begin{aligned} f\Big (\tfrac{\textrm{sd}_{F}}{\tau }\Big ) > -(\kappa _{F_\tau }^\varphi + g) \quad \hbox { on}\ \partial F_\tau . \end{aligned}$$
  Then $$\partial E_\tau \cap \partial F_\tau =\emptyset .$$
- Let $$F\subset E,$$ $$F_\tau \subset E_\tau $$ and
  $$\begin{aligned} f\Big (\tfrac{\textrm{sd}_{F}}{\tau }\Big ) < -(\kappa _{F_\tau }^\varphi + g) \quad \hbox { on}\ \partial F_\tau . \end{aligned}$$
  Then $$\partial E_\tau \cap \partial F_\tau =\emptyset .$$

### Proof

We prove only (a), assertion (b) being similar. By contradiction, assume that $x_0\in \partial E_\tau \cap \partial F_\tau .$ Since $\varphi$ is smooth and elliptic, repeating the arguments of [22, Chapter 17] we can show that singular minimal cones locally minimizing $\varphi$-perimeter cannot contain a halfspace. By the smoothness of $F_\tau ,$ $x_0\in \partial ^*E_\tau .$ Moreover, by elliptic regularity, $\partial ^*E_\tau$ is $C^2,$ and hence, computing the first variation of ${\mathcal {F}}(\cdot ; E,\tau )$ at $E_\tau$ we get

B.2 $$\begin{aligned} f\Big (\tfrac{\textrm{sd}_{E}(x_0)}{\tau }\Big ) = -(\kappa _{F_\tau }^\varphi (x_0) + g(x_0)). \end{aligned}$$

By the choice of $x_0,$ $\kappa _{E_\tau }^\varphi (x_0)\ge \kappa _{F_\tau }^\varphi (x_0)$ and by assumption $E\subset F,$ $\textrm{sd}_{E}(x_0) \ge \textrm{sd}_{F}(x_0).$ Therefore, combining (B.2) and (B.1) we get

$$\begin{aligned} f\Big (\tfrac{\textrm{sd}_{F}(x_0)}{\tau }\Big ) > -(\kappa _{F_\tau }^\varphi (x_0) + g(x_0)) \ge -(\kappa _{E_\tau }^\varphi (x_0) + g(x_0))= f\Big (\tfrac{\textrm{sd}_{E}(x_0)}{\tau }\Big ), \end{aligned}$$

a contradiction. $$\square $$

Comparison principles for (1.13) are well-established provided that the prescribed mean curvatures are comparable.

### Theorem B.2

(Comparison principles) Let $f_1,f_2,g_1,g_2$ satisfy (1a) and (1c). Let $F_1,F_2\in \mathcal {S}^*$ and $\tau >0.$ The following properties hold:

(i) If $F_1\subset F_2,$ $f_1\ge f_2$ and $g_1>g_2$ a.e. in ${\mathbb {R}}^n,$ then minimizers $F_\tau ^i$ of $\mathcal {F}_{\varphi ,f_i,g_i}(\cdot ;F_i,\tau )$ satisfy $F_\tau ^1\subset F_\tau ^2.$
(ii) If $F_1\Subset F_2,$ $f_1\ge f_2$ and $g_1\ge g_2$ a.e. in ${\mathbb {R}}^n,$ then minimizers $F_\tau ^i$ of $\mathcal {F}_{\varphi ,f_i,g_i} (\cdot ;F_i,\tau )$ satisfy $F_\tau ^1\subset F_\tau ^2.$
(iii) If $F_1\subset F_2,$ $f_1\ge f_2$ and $g_1\ge g_2$ a.e. in ${\mathbb {R}}^n,$ then there exist minimizers $F_{\tau *}^1$ of $\mathcal {F}_{\varphi ,f_1,g_1}(\cdot ;F_1,\tau )$ and $F_\tau ^{2*}$ of $\mathcal {F}_{\varphi ,f_2,g_2}(\cdot ;F_2,\tau )$ such that $F_{\tau *}^1\subset F_\tau ^2$ and $F_\tau ^1\subset F_{\tau }^{2*}$ for all minimizers $F_\tau ^i$ of $\mathcal {F}_{\varphi ,f_i,g_i}(\cdot ;F_i,\tau ).$

Indeed, assumptions of (i) and (ii) imply that $h_{\varphi ,f_1,g_1}>h_{\varphi ,f_2,g_2}$ a.e. in ${\mathbb {R}}^n,$ while (iii) implies $h_{\varphi ,f_1,g_1}\ge h_{\varphi ,f_2,g_2},$ see (1.14). Thus the proof follows from standard arguments (see e.g. [9] and references therein).

### Corollary B.3

(Minimal and maximal minimizers) Let $f$ satisfy (1a) and $g$ satisfy (1c), and let $\tau >0$. Then for any $F\in \mathcal {S}^*$ there exist minimizers $F_{\tau *},F_\tau ^*$ (called the minimal and maximal minimizer) of ${\mathcal {F}}(\cdot ;F,\tau )$ such that for every minimizer $F_\tau ,$

$$\begin{aligned} F_{\tau *} \subseteq F_\tau \subseteq F_\tau ^*. \end{aligned}$$

### Corollary B.4

Assume that $g\equiv 0,$ $F_1\Subset F_2$ with $\textrm{dist}(\partial F_1,\partial F_2)=\epsilon >0.$ Then the minimizers $F_\tau ^i$ of ${\mathcal {F}}(\cdot ;F_i,\tau )$ satisfy $F_\tau ^1\Subset F_\tau ^2$ and $\textrm{dist}(\partial F_\tau ^1,\partial F_\tau ^2)\ge \epsilon .$

Indeed, since $g \equiv 0$, ${\mathcal {F}}(E;F,\tau ) = {\mathcal {F}}(E+\xi ;F+\xi ,\tau )$ for any $\xi \in {\mathbb {R}}^n.$ By assumptions on $F_1$ and $F_2,$ for any $\xi \in B_\epsilon (0)$ we have $F_1+\xi \Subset F_2,$ and hence $F_\tau ^1 + \xi \Subset F_\tau ^2$ by Theorem B.2 (b). Thus, $\textrm{dist}(\partial F_\tau ^1,\partial F_\tau ^2)\ge \epsilon .$

As in [9] we can introduce a comparison principle for two $\textrm{GMM}$s.

### Theorem B.5

Let $f_1,f_2,g_1,g_2$ satisfy Hypothesis (H) with $f_1\ge f_2$ and $g_1\ge g_2.$ Let $F_1^0,F_2^0\in \mathcal {S}^*$ be such that $F_1^0\subset F_2^0.$ Then:

- for any $F_1(\cdot )\in \textrm{GMM}(\mathcal {F}_{\varphi ,f_1,g_1},\mathcal {S}^*,F_1^0)$ there exists $F_2^*(\cdot )\in \textrm{GMM}(\mathcal {F}_{\varphi ,f_2,g_2},\mathcal {S}^*,F_2^0)$ such that
  $$\begin{aligned} F_1(t) \subset F_2^*(t),\quad t\ge 0; \end{aligned}$$
- for any $F_2(\cdot )\in \textrm{GMM}(\mathcal {F}_{\varphi ,f_2, g_2},\mathcal {S}^*,F_2^0)$ there exists $F_{1 *}(\cdot )\in \textrm{GMM}(\mathcal {F}_{\varphi ,f_1,g_1},\mathcal {S}^*,F_1^0)$ such that
  $$\begin{aligned} F_{1 *}(t) \subset F_2(t),\quad t\ge 0. \end{aligned}$$

We sketch the proof of only (a). Let $\{F_1(\tau _j,k)\}$ be flat flows starting from $F_1^0$ such that

$$\begin{aligned} \lim \limits _{j\rightarrow +\infty } |F_1(\tau _j,\left\lfloor t/\tau _j \right\rfloor ) \Delta F_1(t)| = 0. \end{aligned}$$

Let also $F_2^*(\tau _j,k)$ be the flat flows starting from $F_2^0$ consisting of the maximal minimizers of $\mathcal {F}_{\varphi ,f_2,g_2}.$ By Theorem B.2 (d)

B.3 $$\begin{aligned} F_1(\tau _j,k)\subset F_2^*(\tau _j,k),\quad k\ge 0,\,j\ge 1. \end{aligned}$$

Now, consider the sequence $(F_2^*(\tau _j,\left\lfloor t/\tau _j \right\rfloor )).$ In the proof of Theorem 1.4 we have constructed a not relabelled subsequence and a family $F_2^*(\cdot )\in \textrm{GMM}({\mathcal {F}}_{\varphi ,f_2,g_2},\mathcal {S}^*,F_2^0)$ such that

$$\begin{aligned} \lim \limits _{j\rightarrow +\infty } |F_2^*(\tau _j,\left\lfloor t/\tau _j \right\rfloor ) \Delta F_2^*(t)| = 0 \qquad \forall t \ge 0 \end{aligned}$$

(see also Remark 3.3). Now, the inclusion $F_1(\cdot )\subset F_2^*(\cdot )$ follows from (B.3).

## Footnotes

1 We have slightly modified the notation with respect to the original conjectures.

2 We slightly abuse the notation: in contrast to the functionals ${\mathcal {G}}$ and $\widetilde{\mathcal {G}}$ in Conjectures 1.1 and 1.2, we do not highlight the dependence on *k*.

3 Note that $r_\tau$ depends on $\varsigma _0,$ $\varsigma _1$ and $\rho _\tau .$

4 Thus, in this case, $\textrm{GMM}$ is unique for short times.

5 Sometimes ${B_\varphi }$ is called Wulff shape.

6 In the case of the standard Almgren–Taylor–Wang functional one can choose $u(s)=s^{1/2}$ for any $s \ge 0$ and get the 1/2-Hölder continuity of the flow (best exponent).

7 Let $A\ge 1,$ $B\ge 0$ and $\{\alpha _k\}_{k\ge 0}\subset \mathbb {R}_0^+$ be such that $\alpha _{k+1} \le A\alpha _k +B$ for any $k\ge 0$. Then $\alpha _m \le A^m\alpha _0 + B\,\frac{A^m-1}{A-1}$ for any $m\ge 1$. Indeed

$$\begin{aligned} \alpha _m \le&A\alpha _{m-1} + B\le A(A\alpha _{m-2} + B) +B = A^2\alpha _{m-2} + B(1+A)\\ \le&\ldots \le A^m\alpha _0 + B(1+A+\ldots +A^{m-1}) = A^m\alpha _0 + B\,\frac{A^m-1}{A-1}. \end{aligned}$$

8 For instance, $(r,s,a,t)\mapsto \textrm{sd}_{L^\pm [r,s,a,t]}$ smoothly varies, see also [2, Corollary 7.2].

9 If $a+\tau >T$ the statement becomes trivial.

10 Concerning the notations $z \cdot Dv_0$ and $\varphi ^o(Dv_0)$, see [11].
